# Suitability and safety of L-5-methyltetrahydrofolate as a folate source in infant formula: A randomized-controlled trial

**DOI:** 10.1371/journal.pone.0216790

**Published:** 2019-08-19

**Authors:** Barbara Troesch, Johann Demmelmair, Martina Gimpfl, Christina Hecht, Goran Lakovic, Robert Roehle, Ljilja Sipka, Branka Trisic, Milica Vusurovic, Rotraut Schoop, Sznezana Zdjelar, Berthold Koletzko, on behalf of the MEFOLIN Study Group

**Affiliations:** 1 DSM Nutritional Products Ltd., Kaiseraugst, Switzerland; 2 LMU -Ludwig-Maximilians Universität Munich, Dr von Hauner Children’s Hospital, Munich, Germany; 3 HiPP GmbH & Co. Vertrieb KG, Pfaffenhofen, Germany; 4 Clinical Hospital Center "Dr Dragiša Mišović-Dedinje", Belgrade, Serbia; 5 Charité- Universitätsmedizin Berlin, corporate member of Freie Universität Berlin, Humboldt-Universität zu Berlin, Berlin, Germany; 6 Berlin Institute of Health, KKS Charité, Berlin, Germany; 7 HiPP Study Center, Belgrade, Serbia; University of Ghana, GHANA

## Abstract

L-5-methyltetrahydrofolate is the predominant folate form in human milk but is currently not approved as a folate source for infant and follow-on formula. We aimed to assess the suitability of L-5-methyltetrahydrofolate as a folate source for infants. Growth and tolerance in healthy term infants fed formulae containing equimolar doses of L-5-methyltetrahydrofolate (10.4 μg/ 100 ml, n = 120, intervention group) or folic acid (10.0 μg/ 100 ml, n = 120, control group) was assessed in a randomized, double-blind, parallel, controlled trial. A reference group of breastfed infants was followed. Both formulae were well accepted without differences in tolerance or occurrence of adverse events. The most common adverse events were common cold, poor weight gain or growth, rash, eczema, or dry skin and respiratory tract infection. Weight gain (the primary outcome) was equivalent in the two groups (95% CI -2.11; 1.68 g/d). In line with this, there was only a small difference in absolute body weight adjusted for birth weight and sex at visit 4 (95% CI -235; 135 g). Equivalence was also shown for gain in head circumference but not for recumbent length gain and increase in calorie intake. Given the nature of the test, this does not indicate an actual difference, and adjusted means at visit 4 were not significantly different for any of these parameters. Infants receiving formula containing L-5-methyltetrahydrofolate had lower mean plasma levels of unmetabolized folic acid (intervention: 0.73 nmol/L, control: 1.15 nmol/L, *p*<0.0001) and higher levels of red cell folate (intervention: 907.0 ±192.8 nmol/L, control: 839.4 ±142.4 nmol/L, *p* = 0.0095). We conclude that L-5-methyltetrahydrofolate is suitable for use in infant and follow-on formula, and there are no indications of untoward effects.

**Trial registration:** This trial was registered at ClinicalTrials.gov (NCT02437721).

## Introduction

The vitamin folate is essential for the synthesis of RNA and DNA, and for cell division and tissue growth [1]. An adequate folate status is crucial for the rapid growth and development during pregnancy and infancy. L-5-methyltetrahydrofolate (L-5-MTHF) is the active form of folate that provides one carbon units used for the synthesis of myelin, neurotransmitters and phospholipids, all of which are essential components for normal neurodevelopment [2]. An impaired one-carbon metabolism may limit the availability of the omega-3 long chain polyunsaturated fatty acid docosahexaenoic acid to the brain, which is also needed for normal brain development [3].

Currently, folic acid is the only folate source approved for use in infant formula and follow-on formula in the European Union [4, 5]. Folic acid is a synthetic form of folate which does not occur in nature and is rarely found in unfortified foods [2]. In breast milk, the predominant folate species is L-5-MTHF [6]. Unmetabolized folic acid is not detectable in breast milk unless there are high maternal intakes of folic acid supplements or of foods fortified with folic acid [6, 7]. Therefore, breastfed infants predominantly ingest L-5-MTHF. Short-term bioavailability of L-5-MTHF and folic acid are generally comparable [8], although only limited information is available in infants. Some evidence indicates a more efficient increase of plasma [9, 10] and red cell folate (RCF) levels by L-5-MTHF as compared to folic acid [11, 12].

Conversion of folic acid into L-5-MTHF appears to occur predominantly in the liver, while some unmetabolized folic acid appears in the systemic circulation [13]. This is probably most pertinent when intakes are high as folic acid seems to pass unchanged into the peripheral circulation at doses above 200 μg in human adults [14]. However, even the relatively low levels found in infant formula have led to a significant increase in the levels of unmetabolized folic acid in the plasma of formula fed infants [15]. It has been suggested by *in vitro* and animal studies that unmetabolized folic acid may have adverse health effects, although the evidence remains somewhat controversial [16]. Therefore, the suitability of using L-5-MTHF as an alternative folate source for infant and follow-on formula should be investigated.

In the present double blind randomized trial, we assessed growth, tolerance and indicators of safety in infants fed a formula containing L-5-MTHF compared to infants fed a standard formula with folic acid. In addition, a reference group of breastfed infants was followed. We aimed to assess the suitability and safety of L-5-MTHF as a folate source in infant formula.

## Methods

### Study design

The study was designed as a single center, randomized, double-blind, parallel-group, controlled clinical trial in apparently healthy term infants receiving infant formula containing either L-5-MTHF (intervention group) or folic acid (control group) as the folate source. Since breastfeeding is the gold standard for infant nutrition, a group of healthy term breastfed infants whose mothers intended to breastfeed for at least 4 months was included. However, as these infants could not be randomized, they were not included in the statistical analysis and only served as a reference group. A comparable number and types of (serious) adverse events in the intervention and reference group can be regarded as an indicator for safety of the intervention.

Infants were recruited from delivery until the age of 27 days. Infants of parents who independently chose not to breastfeed their healthy newborn babies for reasons not related to this study, or who decided to start full formula-feeding within the first 28 days of life, were randomized into one of the two formula groups. Infants received a Randomization Number from the random list (Random Block Size = 6) stratified according to gender (male = random numbers 1–150 and female = random numbers 151–300), starting at top of the lists, with 1, which corresponds to random number R001. The random number list obtained from the University of Munich Medical Centre were provided directly to the logistic partner responsible for blinding the study formulae (SCA Full Filement Ltd, Bruhnstrasse 15, 85053 Ingolstadt). The study team, the investigators as well as the parents and caregivers were blinded to the formula group.

Great care was taken by the study team to encourage, promote and protect breastfeeding. Formula feeding was not promoted at any time. Parents were only approached with the suggestion to enroll their infants in the formula arms of the study after they had independently decided to use formula feeding.

At the baseline visit (BV: age 1 to 27 days), a medical examination was performed, anthropometric data (weight, length and head circumference) was obtained and an evaluation of inclusion and exclusion criteria was conducted. Blood samples were collected for folate status, blood chemistry and hematology profiles for all three groups.

At visit 1 (V1: age 28 ±3 days), visit 2 (V2: age 56 ±3 days), visit 3 (V3: age 84 ±3 days) and visit 4 (V4: age 112 ±3 days) infants were examined and anthropometric data was collected. During the three days prior to each visit, parents recorded the volume of formula as well as other foods consumed in a standardized prospective three-day food protocol and completed questionnaires on formula acceptance, stool frequency, consistency, color and smell, crying and sleeping behavior as well as occurrence of belching, posseting, vomiting and bloating.

Body weight was measured using a calibrated digital scale (SECA 336, Hamburg, Germany), recumbent length was determined using a stationary, vertical headboard, and head circumference was measured with a non-stretchable insertion tape. All weight, length and head circumference measurements were repeated and recorded twice after excluding any clearly erroneous value.

### Study population

From June 2015 to April 2017, 360 infants aged <28 days were enrolled at the Department of Neonatology, Clinical Hospital Center "Dr Dragiša Mišović-Dedinje", Belgrade, Serbia. Eligible infants had to be apparently healthy from singleton pregnancies, delivered at between ≥37 and ≤41 weeks of gestation, with a birth weight between 2500 and 4500 g.

### Study diets

Study formulae (HiPP GmbH & Co. Vertrieb KG, Pfaffenhofen, Germany) were given free of charge to the parents or caregivers in 500 g paper boxes, each containing two inner bags and labelled by random numbers. Except for L-5-MTHF in the intervention formula, the composition of the intervention and the control infant formula were identical and met the regulatory standards established by the European Commission [4] (Table 1 and S1 Table).

**Table 1:** Composition of intervention and control infant formula (excerpt, full details are shown in S1 Table).

	Unit	Per 100 g powder	Per 100 kcal^a^	Per 100 ml reconstituted formula
Energy	kJ	2134		277
	Kcal	510		66
Protein	g	9.6	1.9	1.25
Carbohydrates	g	56.1	11.1	7.3
Fat	g	27.0	5.3	3.5
Folic acid^b^	μg	78	15.2	10
L-MTHF^c^	μg	81	15.8	10.4

^a^ The nutrient contents per 100 kcal were calculated on basis of the nutrient contents as well as the energy content per 100ml ready-made formula

^b^control and

^c^intervention formula; MTHF: L-5-methyltetrahydrofolate

The current European legislation for infant and follow-on formula defines a required folic acid content of 10 to 50 μg/100 kcal and an energy content of 60 to 70 kcal/100 g reconstituted formula [4]. Accordingly, the control formula contained 10.0 μg folic acid per 100 ml reconstituted infant formula (15.2 μg folic acid per 100 kcal). As L-5-MTHF is currently not an approved folate source for infant and follow-on formula, no recommend levels of content in such products have been defined by regulatory bodies. Previous research indicates comparable bioavailability and activity for folic acid and MTHF at equimolar doses [17]. Consequently, to match the 22.7 nmol folic acid (10.0 μg/ 100 ml), 10.4 μg/ 100 ml L-5-MTHF were used in the intervention formula (conversion factor 1.04) which was added as 11.3 μg of the calcium salt of L-5-MTHF (Metafolin, Merck & Cie, Schaffhausen, Switzerland [18]). To confirm the stability of calcium L-MTHF during the storage period, samples of powdered intervention formula were taken for quantification of folate immediately after production then after 1, 2, 3, 4, 7, 10, 12, 15 and 18 months. In addition, the formula was prepared according to standard preparation instructions and the stability of folate in the prepared product was also examined. All samples were analyzed according to the AOAC 992.05 method for Folic acid (Pteroylglutamic acid) in infant formula. The stability test of L-MTHF in powered infant formula demonstrated that folate concentration was stable at room temperature for 18 months. The stability evaluation of L-MTHF in prepared product showed no loss in folate concentration during the infant formula preparation process.

In line with current recommendations [19], parents and caregivers were advised to provide formula ad libitum.

### Laboratory procedures

At BV and V4, venous blood samples were drawn to determine folate status as well as blood chemistry and hematology. All lab procedures were performed by lab staff that were blinded for all subject related data including group allocation. The blood samples used for folate status analysis were centrifuged, plasma was frozen at -80°C and sent on dry ice to the analytical laboratory (Bevital AS, Bergen, Norway). The assay was performed using LC-MS/MS and was adapted to determine folate species (L-5-MTHF, unmetabolized folic acid) and folate catabolites (formyltetrahydrofolate (fTHF), 4-alpha-hydroxy-5-methyltetrahydrofolate (hmTHF), para-aminobenzoylglutamate (pABG), and acetamidobenzoylglutamate (apABG)) in plasma as previously reported [20]. In addition, the corresponding 13C-labeled internal standards were used for all the analytes. During sample preparation, ascorbic acid was added to the plasma sample to avoid oxidation of folate. If any L-5-MTHF had been oxidized to 5-methyl-5,6-dihydrofolate before sample preparation, it would be reduced back to L-5-MTHF after the addition of ascorbic acid. The samples were deproteinized by acetonitrile. Because acetonitrile causes peak broadening on a C8-column, the supernatant was evaporated, and the analytes were re-dissolved in water. For total RCF, the microbiological assay performed used microtiter plates and a chloramphenicol-resistant strain of *Lactobacillus casei* [21].

Genomic DNA was obtained from white blood cells, which became available after the collection of EDTA blood. The blood samples were sent from the study site in Belgrade to the laboratory (DSM Nutritional Products Ltd., Kaiseraugst, Switzerland) on dry ice for analysis. Fast genomic DNA extraction was performed using the spin column procedure, which was then analyzed using the Qiamp DNA blood mini kit (Qiagen, Basel, Cat No. 51104). This method included an initial lysis step of the white blood cells with a consecutive RNase and protease treatment. The genomic DNA was transferred and washed on a Qiamp spin column. After several washing steps the purified genomic DNA was harvested in elution buffer. DNA quantity and purity were controlled using the NanoDrop instrument.

For genotyping, the Applied Biosystems TaqMan SNP genotyping assay was used according to the manufacturer’s instructions (Cat No. 4351379). The universal master-mix was mixed with 10 ng of genomic infant DNA in a volume of 20 μl and the samples were processed in the ABI 7900 instrument. The samples were measured in 96-well plates using the respective quality controls [22]. Both alleles of the two methyltetrahydrofolate reductase (MTHFR, NCBI gene reference: NM_005957.4) single nucleotide polymorphisms (SNPs) (MTHFR C677T (SNP rs1801133, Assay-ID: C_1202883_20; context sequence [VIC/FAM]: GAAAAGCTGCGTG ATGATGAAATCG**[G/A]**CTCCCGCAGACACCTTCTCCTTCAA) and MTHFR A1289C (SNP rs1801131, Assay-ID: C_850486_20; context sequence [VIC/FAM]: AAGAACGAAGACTTCAAA GACACTT**[G/T]**CTTCACTGGTCAGCTCCTCCCCCCA)) were determined.

### Power calculation

The primary objective of the study was to show equivalence for the intervention and the control group. Weight gain between V1 and V4 was used as the primary outcome. Based on the recommendation of the Scientific Committee on Food of the European Commission [23], an average daily weight gain of infants in the intervention group was considered equivalent to the average daily weight gain of infants in the control group, if it was within the boundaries of ±0.5 standard deviation (SD). Based on previous observations in Serbian infants [24], the infants’ weight gain was estimated to be ~30 g per day with a SD of ~7g. Sample size was calculated using the approach described by Julious [25] for equivalence clinical trials with normal data in the case of no treatment difference (Required power of 80%, significance level for the two one-sided test procedure 2.5%), resulting in a sample size of 84 analyzable infants per group. Based on the assumption of a drop-out rate of 30%, it was estimated that 120 infants per group were needed at V1.

### Statistical analysis

The primary (weight gain) and secondary (gain in length, head circumference and calorie intake) outcomes were analyzed for per-protocol (PP) (primary analysis) and modified intention-to-treat (mITT) populations. In the PP analysis, only data from subjects who completed all visits without any protocol deviations were included. In the mITT analysis, all subjects were considered who completed V1, thus all those for whom at least one data point was available. The major reasons for exclusion from the PP population was missing the target of ±3 days for V1 to V4 or intake of foods other than the investigational product or breast milk in the formula and the reference group, respectively, exceeding 50 ml on >50% of days captured in the 3-day diaries.

To show equivalence regarding growth parameters between the control and the intervention groups, a linear mixed model on the respective (longitudinally measured) growth parameter was used. It included treatment, age, age^2^ (only for body weight), and the age-treatment interaction as fixed effects, birth weight and sex as adjusting covariates and a random intercept and random slope (age) per subject was fit. In this model, the coefficient for the age-treatment interaction can be interpreted as the difference in mean daily gain for the respective parameter between intervention and control group. For the primary analysis following Schuirman [26] and Wellek [27], both groups were considered equivalent if the 95% CI for the difference of the mean daily weight gain was within the pre-specified equivalence margin of ±3.5 g/d. This corresponds to a two one-sided test procedure taking the cut-off level of statistical significance at 0.025. A subgroup analysis was performed by adding three-way interactions with treatment and age for sex and polymorphisms. For gain in recumbent height and head circumference as well as increase in calorie intake from V1 to 4, clinical equivalence was demonstrated if the 95% CI of the difference between intervention and control group was contained within ±0.5 SD of control group.

Results for the equivalence analysis are presented as mean daily gain of the respective parameters with 95% CI, together with the respective equivalence margins. Continuous demographic and baseline characteristics were compared using t-tests or Mann-Whitney-U-tests dependent on their distribution. Results for these are presented as mean ± SD or medians with 25^th^ and 75^th^ percentile. Categorical endpoints were compared using Chi-Square tests or Fisher’s exact test where appropriate. P-values below 0.05 were considered as statistically significant although only the primary analysis can be viewed as confirmatory.

Given the nature of the test, analysis of PP is frequently regarded as the more conservative approach in equivalence testing [28]. Therefore, we used the PP analysis as our primary analysis and the mITT for confirmatory purposes (see S3 Table, S6 Table, S7 Table, S8 Table and S9 Table).

### Data management

To capture and transfer the study data to a central database we used secuTrial (Version 5.2.0.13), a Remote Data Entry software solution of interActive Systems. A validated installation of the statistical program package SAS version 9.4 [SAS (TS1M0) SAS Institute Inc., Cary, NC, USA Copyright (c) 2002–2012] was used for statistical analysis while R version 3.2.2. [29] was used for graphical illustrations.

### Ethical considerations

The study was approved by the independent ethics committee of the University Hospital *“Dr Dragiša Mišović-Dedinje”* in Belgrade, Serbia. Written informed consent was obtained from parents or caregivers of infants who were judged to fulfil the inclusion and exclusion criteria for enrolment into this clinical study prior to their inclusion. Investigators and the study team took special precautions and care throughout their communication with the families in order to support and protect, and not to discourage breastfeeding, and not to suggest bottle feeding to parents of a breastfed baby.

The study was registered at ClinicalTrials.gov (NCT02437721).

## Results

**Fig 1:**
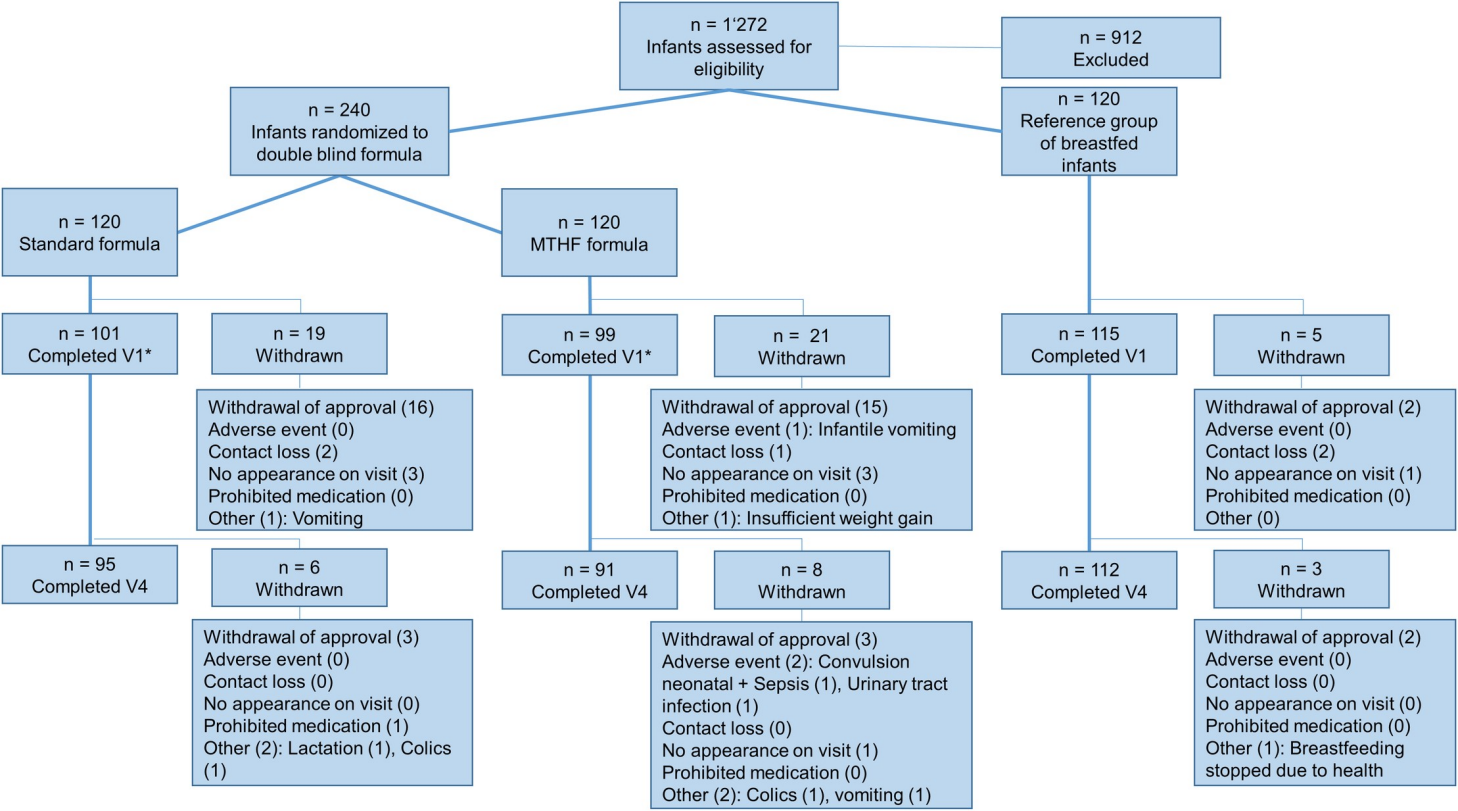
Participant flow chart; n: Number of participants; V1-4: Visits 1 to 4.

A total of 360 infants were recruited, including 240 infants who were randomly allocated either to the intervention or the control formula, as well as 120 breastfed infants who served as a reference group (Fig 1). Of these infants, 315 and 298 completed V1 and V4, respectively. No relevant difference in the number of drop-outs was detected between the intervention and the control group. Main reasons were withdrawal of consent (n = 33) and loss to follow-up (n = 9). One infant in the intervention group died during the study for reasons unrelated to the intervention (see below).

At baseline, the infants in the two formula groups were comparable with the exception of a significant difference in age and the distribution pattern of the MTHFR A1289C genotype (Table 2). No difference in age was observed at V1-4 as the timing of the subsequent visits was set based on defined ages of the infants.

**Table 2:** Baseline characteristics in the per-protocol population: Age, sex and MTHFR polymorphisms C677T (rs1801133) and A1289C (rs1801131) (data for modified intention-to-treat population in S2 Table).

Parameter	n	Intervention group	Control group	*P* value^1^	Reference group^2^
Age [d]	244	21.0 ±3.7	19.4 ±3.4	0.0056	20.0 ±3.3
Sex [% females]	244	43.7%	44.6%	0.9091	52.2%
C677T	238				
CC		33.3%	33.3%		39.8%
CT		53.6%	56.8%		42.0%
TT		13.0%	9.9%	0.8198	18.2%
A1289C	238				
AA		46.4%	44.4%		63.6%
AC		33.3%	49.4%		27.3%
CC		20.3%	6.2%	0.0168	9.1%

Data presented as mean ± SD or percentage; MTHFR: methyltetrahydrofolate reductase n: number of subjects

^1^ Intervention and control group was compared, p <0.05 was considered significant, t-test for unequal variance used for age, Chi square test used for sex and genotype

^2^The mothers decided whether they wanted to breastfeed their infants and consequently, the infants in this group were not randomized. Therefore, no formal statistical comparison is possible.

### Safety and acceptance of the tested formulae

Both formulae were well-accepted and no differences in acceptance and tolerability (S3 Table) or consistency, color and smell of stool (S1 Fig) were reported. Neither (serious) adverse events (S4 Table) nor blood chemistry and hematology results (S5 Table) gave reason for any safety concerns. All those results were within the expected range of our target study population and comparable between the intervention and the control group. The most common adverse events were common cold (28%), poor weight gain or growth (21%), rash, eczema, or dry skin (9%) and respiratory tract infection (9%) and were all similarly distributed between both formula groups.

A total of 89 adverse events and 29 serious adverse events were reported in the study, most of which were typical events expected to occur in otherwise healthy infants. Of these, 3 were judged to be related to formula or breast-milk intake. The parents of one infant in the intervention group believed their child did not tolerate the formula. After switching the infant to a commercial formula, the observed problems resolved. Overweight was reported in one infant in the control group at V4. However, based on follow-up data at the age of one year, the infant’s weight was found to be within the age-appropriate ranges. One breastfed infant in the reference group showed poor growth, which was resolved after switching to feeding a standard infant formula.

One infant from the intervention group was hospitalized due to an infection with Staphylococcus aureus, which resulted in meningitis, neonatal septicemia and eventually death. Analysis of the event led to the conclusion that there was no relationship between the intervention and the death of this infant.

### Folate status

Most markers for folate status did not differ between the intervention and control groups at BV or V4 (Table 3). However, at V4, plasma level of unmetabolized folic acid was significantly higher in the control compared to intervention group, with comparable concentrations of unmetabolized folic acid in the intervention and breastfed groups. RCF was significantly higher in the intervention compared to control subjects. This was reflected in a significant difference in the adjusted mean for RCF of 70.5 nmol/ L between intervention and control groups (least square mean, p = 0.0158). Due to a large number of samples with values below the level of quantification, a least square mean analysis could not be performed for unmetabolized folic acid.

**Table 3:** Folate status at Baseline and Visit 4 in the per-protocol population.

	n	Intervention group	Control group	*P* value^1^	Reference group^2^
L-5-MTHF [nmol/ L]					
Baseline visit	232	26.5 ±12.2	26.2 ±12.5	0.8161	26.1 ±14.8
Visit 4	236	55.3 ±18.1	52.7 ±19.0	0.6791	33.0 ±17.6
Unmetabolized Folic acid [nmol/ L]					
Baseline visit	232	0.60 (0.53, 0.81)	0.69 (0.53, 0.89)	0.0943	0.53 (0.53, 0.53)
Visit 4	124	0.73 (0.60, 1.00)	1.15 (0.92, 1.36)	<0.0001	0.74 (0.62, 0.87)
hmTHF [nmol/ L]					
Baseline visit	232	7.7 (6.3, 10.3)	8.0 (6.2, 10.8)	0.6648	7.5 (5.2, 10.8)
Visit 4	236	5.9 (4.0, 8.9)	4.8 (3.4, 7.5)	0.1666	3.2 (2.0, 5.2)
pABG [nmol/ L]					
Baseline visit	232	9.0 (5.5, 15.1)	10.0 (7.1, 15.6)	0.3960	7.6 (3.4, 11.6)
Visit 4	236	20.1 (12.8, 24.2)	17.7 (12.1, 24.6)	0.5316	12.3 (7.9, 22.1)
apABG [nmol/ L]					
Baseline visit	232	1.03 (0.84, 1.24)	1.06 (0.95, 1.34)	0.3742	1.02 (0.79, 1.24)
Visit 4	236	0.71 (0.63, 0.81)	0.71 (0.60, 0.84)	0.7471	0.59 (0.50, 0.68)
Total RCF [nmol/ L]					
Baseline visit	240	551.7 ±229.9	597.9 ±217.4	0.6265	627.3 ±256.5
Visit 4	238	907.0 ±192.8	839.4 ±142.4	0.0095	484.2 ±213.0

Data presented as mean ±SD or median (P25, P75); apABG: acetamidobenzoylglutamate; pABG: para-aminobenzoylglutamate; hmTHF: 4-alpha-hdroxy-5-methyltetrahydrofolate; L-5-MTHF: L-5-methyltetrahydrofolate; RCF: red cell folate, n: number of subjects

^1^ Intervention and control group was compared, p<0.05 was considered significant, t-test for equal variance used for L-5-MTHF, t-test for unequal variance used for RCF, Mann-Whitney-U test used for folic acid, hmTHF, pABG, apABG

^2^The mothers decided whether they wanted to breastfeed their infants and consequently, the infants in this group were not randomized. Therefore, no formal statistical comparison was performed.

### Growth

**Fig 2:**
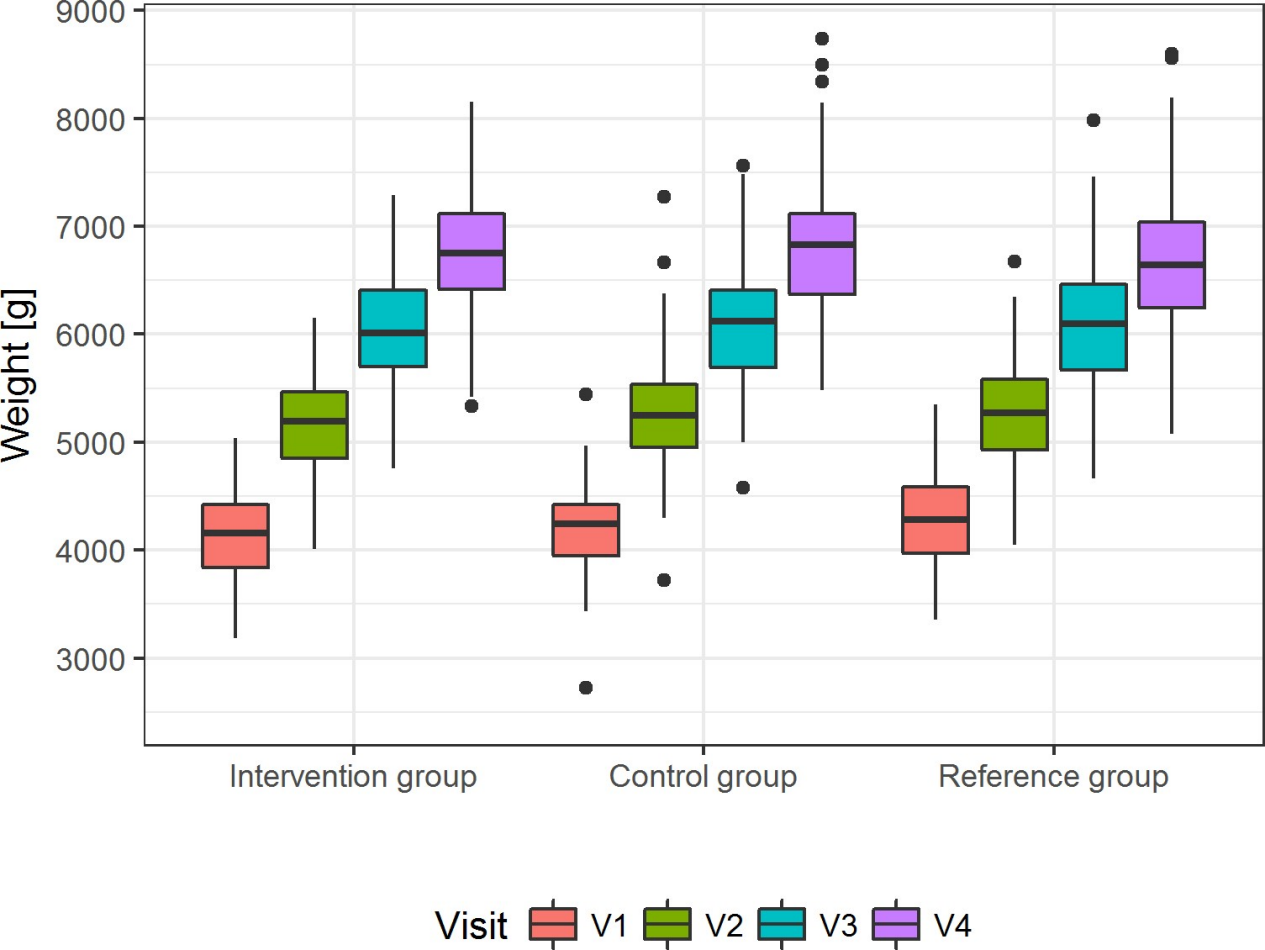
Weight gain in the three groups during the intervention period. Presented are the unadjusted median weights [g] with 25^th^ and 75^th^ percentile, maximum and minimum for visits 1 to 4 in the per-protocol population (*p* = 0.5950 for least-squares means of weight at V4, S8 Table).

The primary outcome, weight gain during the intervention period, is shown in Fig 2. The difference in mean daily weight gain of the intervention and control groups, represented by the 95% CI of the age-treatment interaction, was within the predefined interval of ±3.5 g/day and thus, equivalence was demonstrated (Table 4 and S6–S8 Tables).

**Table 4:** Values of equivalence margins and calculated 95% confidence intervals (age-treatment-interaction) for daily weight gain, length gain, gain in head circumference as well as change in daily calorie intake for the per-protocol population.

Parameter	Equivalence margin	95% CI^1^	
		Lower limit	Upper limit
Linear daily weight gain from V1 to V4 [g/d]	3.5^2^	-2.1102	1.6780
Linear daily growth from V1 to V4 [cm/d]	0.0084^3^	-0.0012	0.0092
Daily Increase in head circumference from V1 to V4 [cm/d]	0.0057^3^	-0.0033	0.0038
Change in daily calorie intake per kilogram from V1 to V4 [kcal/kg]	0.6855^3^	-1.0434	-0.1055

95% CI: 95% confidence interval

^1^ Calculated from linear mixed model additionally including sex and birth weight (S7 Table)

^2^ 0.5 x SD reported in Serbian infants by Fleddermann et al. [24]

^3^0.5 x SD in the control group

Testing adjusted mean body weight at V4 (least square means), the difference between the groups (50.09 g) was not significant (*p* = 0.5950, S8 Table), indicating that after adjustment of birth weight and sex there was no difference between formula groups at V4. Neither sub-group analysis (least square means) by sex (p = 0.4286) nor by MTHFR C677T (p = 0.6992) and A1289T (p = 0.1423) genotypes revealed group specific effects of treatment on weight gain (S9 Table).

**Fig 3:**
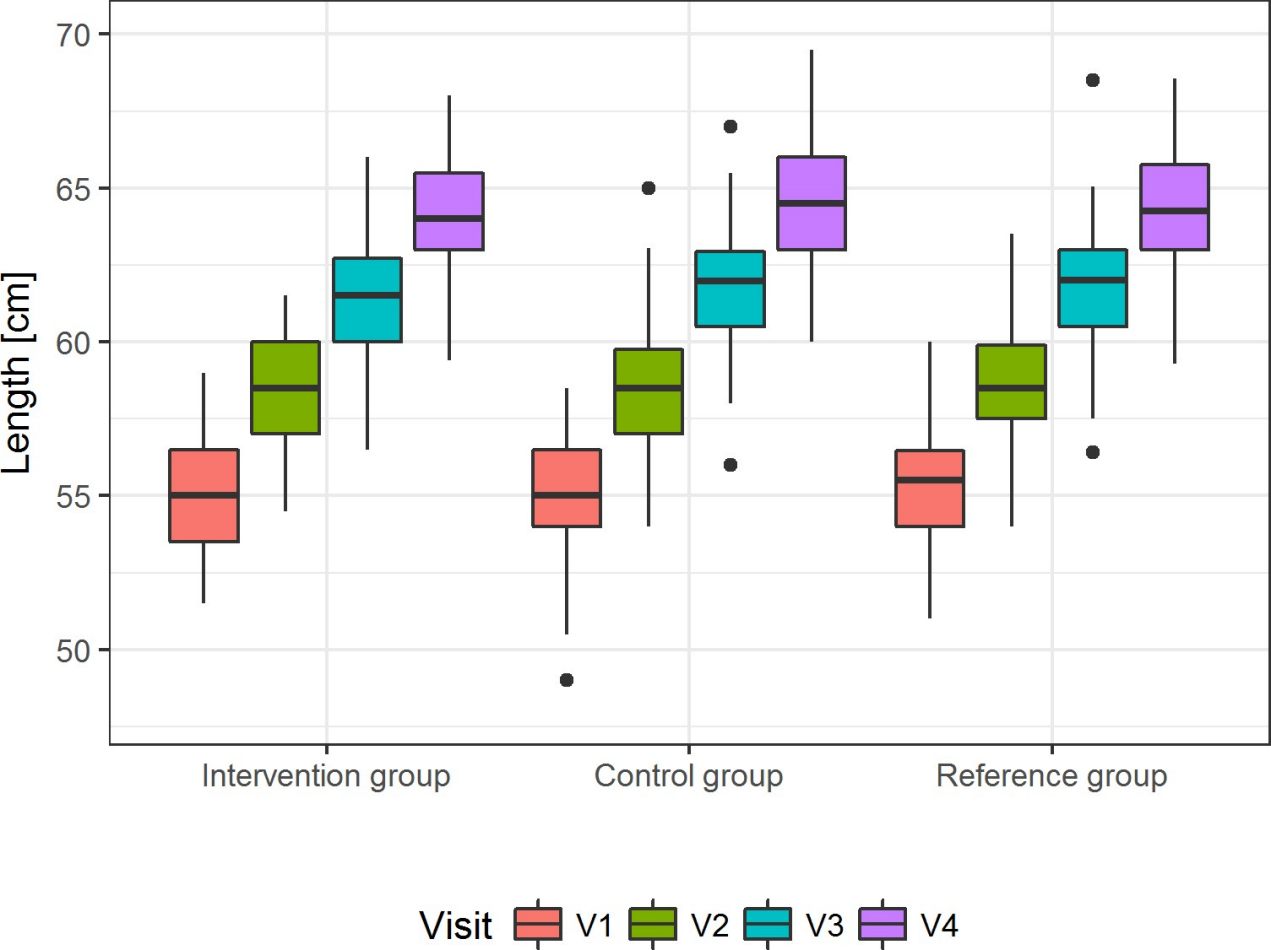
Daily length gain in the three groups during the intervention. Presented is the unadjusted median recumbent length [cm] with the 25^th^ and 75^th^ percentile, maximum and minimum for V1 to V4 in the per-protocol population (p = 0.6115 for least squares means of recumbent length at V4, S8 Table).

The age-treatment-interaction for daily length gain, daily gain in head circumference as well as change in daily calorie intake are shown in Table 4. For daily length gain, the 95% CI for the age-treatment interaction included the value for the 0.5 SD margin (Fig 3 and S7 Table). Consequently, there is not conclusive evidence to support equivalence for length growth, although the upper limit of the 95% CI is just slightly above the calculated margin. Differences in the least square means at V4 were very small (control—intervention: -0.14 ± 0.27 cm) and no difference between the groups was found (*p* = 0.6115, S8 Table).

**Fig 4:**
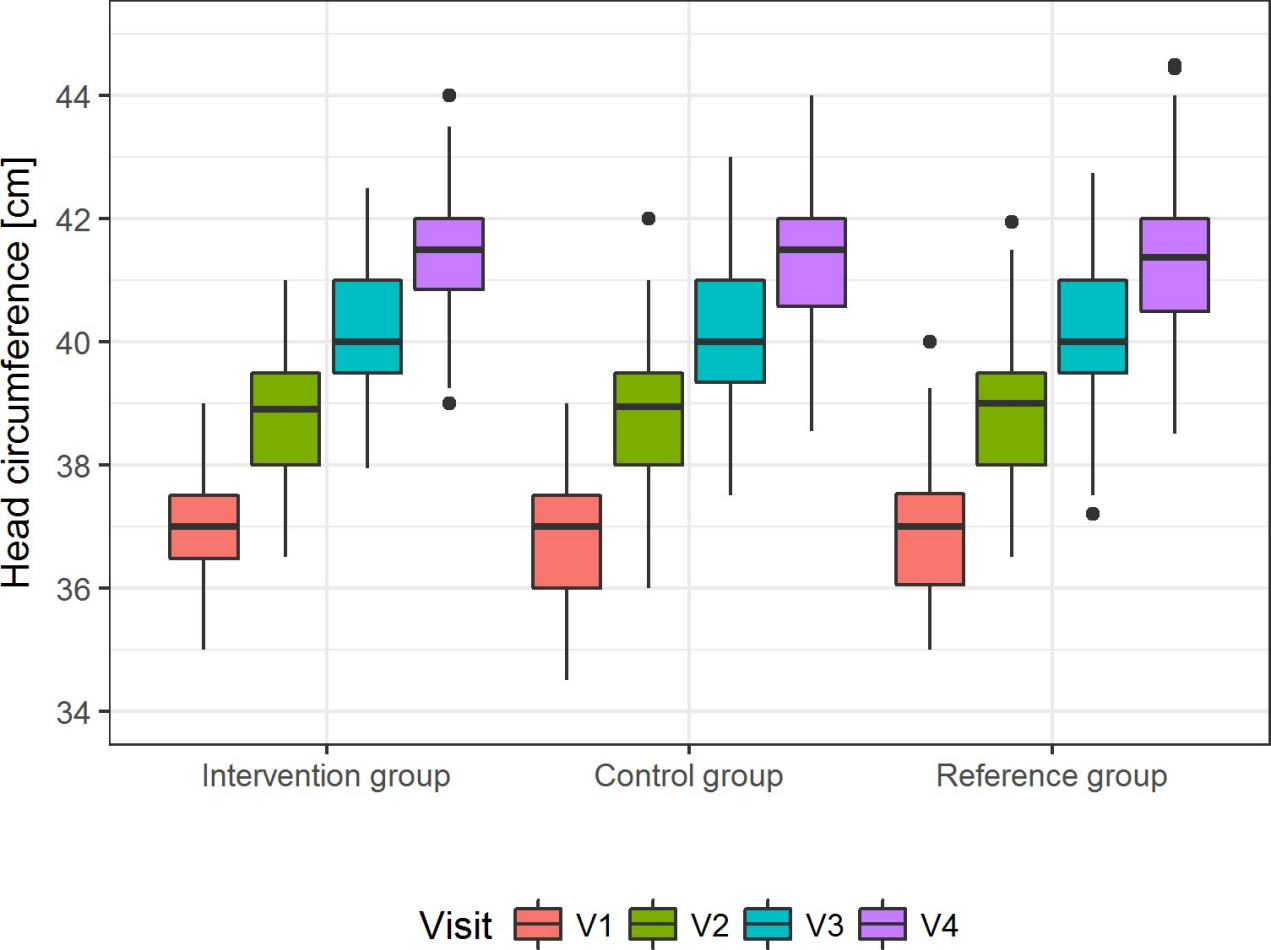
Gain in head circumference in the three groups during the intervention. Shown are the unadjusted median head circumference [cm] with 25^th^ and 75^th^ percentile, maximum and minimum for V1 to V4 in the per-protocol population (*p* = 0.3258 for least-squares means of head circumference at V4, S8 Table).

For daily head circumference gain, the 95% CI for the age-treatment interaction did not include the value for the 0.5 SD margin (Fig 4 and S7 Table) and thus, clinical equivalence can be demonstrated. Moreover, there were very small (control—intervention: 0.16 ± 0.17 cm), non-significant differences in the least square means at V4 (p = 0.3258; S8 Table).

### Calorie intake

For daily calorie change, the 95% CI for the age-treatment interaction included the value for the 0.5 SD margin (Table 4 and S6 Table) and thus, there is no conclusive evidence to demonstrate equivalence. Differences in the least square means at V4 again were very small (control—intervention: 19.9 ±15.1 kcal), and there was no difference between the groups (p = 0.1900, S8 Table). Also, for the other visits there were no group differences (S8 Table).

## Discussion

Although L-5-MTHF is the predominant folate form in breast milk [6], folic acid is currently the only folate source approved for the use in infant and follow-on formula. To our knowledge, this is the first study comparing infant formulae containing L-5-MTHF and folic acid, respectively, at equimolar doses, evaluating their effect on growth, tolerability and indicators of safety in infants.

For weight gain, the primary outcome, equivalence between the intervention and the control group was shown, because the 95% CI of the difference in mean daily weight gain falls within the predefined equivalence margin of ±3.5g/ d. This is also reflected in the small, non-significant difference between mean weights in both groups adjusted for birth weight and sex at V4 (50.09 g, *p* = 0.5950). Equivalence was also demonstrated for the gain in head circumference (secondary outcome) as the 95% CI in the intervention group was less than ±0.5 SD in the control group and the adjusted difference between the two groups was not significant (0.16 ± 0.17 cm, *p* = 0.3258).

For linear growth from V1 to V4 (secondary parameter), evidence on equivalence between the groups was inconclusive. The upper limit of the 95% CI was slightly greater than the equivalence margin (0.0084 cm/day vs. 0.0092 cm/day) and thus, no formal equivalence could be concluded. However, due to the nature of the test, this finding does not confirm an actual difference between the two groups either. Differences in the adjusted mean body lengths (least square means) at V4 were small (- 0.14 cm) and not significant (*p* = 0.6115), suggesting that after adjusting for birth weight and sex, there was no difference in body length between the two groups at visit 4.

Based on the small, non-significant difference in the adjusted length at V4, we consider it unlikely that the difference is of clinical relevance. Moreover, we are not aware of any literature on a physiological mechanism through which L-5-MTHF, but not folic acid, would affect linear growth without affecting weight gain. This is particularly true given that the markers for folate status indicate an at least equivalent bioavailability of L-5-MTHF compared to folic acid.

Equivalence could also not be established for the increase in daily calorie intake per kilogram. However as with linear growth, this does not imply a statistical difference. Caloric intake values are based on the information provided by the parents or caregivers using 3-day-diaries, and therefore provide insight only into a limited time window. Furthermore, 3-days records are known to have methodological shortcomings. While caloric intakes from the study formulae could be calculated rather accurately since they are based on the number of spoons added, caloric intake data from other foods are prone to error. Particularly towards the end of the four months intervention, foods other than infant formula contributed increasingly to daily energy intake, thus making total caloric intake harder to measure. Therefore, failure to establish equivalence for calorie intake might well be due to methodological limitations rather than physiological effects.

During early infancy, breast milk and/or infant formula are the sole sources of nutrition and must supply appropriate amounts of energy, water and all essential nutrients [30]. As folate deficiency has not been reported in breastfed infants, even if maternal status was low, breast milk folate levels are regarded as adequate for this age group. On that basis, the current European legislation for infant and follow-on formula sets a required folic acid content of 10 to 50 μg/100 kcal, while energy content is at 60 to 70 kcal/100 ml formula [4]. In line with this, the control formula contained 10 μg folic acid per 100 ml reconstituted infant formula (15.2 μg folic acid per 100 kcal),

As L-5-MTHF is currently not an approved folate source for infant and follow-on formula, no recommend levels have been defined by regulatory bodies in these products. Previous research indicates comparable bioavailability and activity for folic acid and L-5-MTHF at equimolar doses [17]. Therefore, we used folic acid and L-5-MTHF at equimolar doses in this study.

For formula fed infants, the levels for RCF at V4 were found significantly higher in the intervention than in the control group. RCF is an indicator for longer-term folate status because erythrocytes accumulate folate predominantly during erythropoiesis [31]. To our knowledge, the bioavailability of these two folate sources in young infants have not been compared previously. However, a range of studies in adults comparing the effect of low dose, equimolar L-5-MTHF and folic acid supplements on markers of folate status showed an equal or even higher bioavailability for L-5-MTHF [11, 12, 32, 33].

Given the supply of folic acid in the control group, it is not surprising that the levels of unmetabolized folic acid were significantly higher compared to the infants in the intervention group. This is in line with a study in newborns that reported a significant increase in unmetabolized folic acid after only 4 days of feeding a folic acid containing infant formula [15]. In the light of emerging, albeit controversial preclinical evidence of a potential adverse health effect of unmetabolized folic acid [16], this finding requires further investigation.

L-5-MTHF polyglutamates from natural food sources require hydrolysis of the polyglutamyl chain to L-5-MTHF monoglutamate prior to being absorbed in the human small intestine [34]. Since the calcium salt of L-5-MTHF dissociates to MTHF monoglutamate and Ca^2+^ in aqueous solutions, its digestion, absorption, and transport is thought to be similar to that of L-5-MTHF from natural food sources [35]. Other reduced folates, such as L-5-fTHF, are transformed into L-5-MTHF in the enterocyte after absorption, and L-5-MTHF is subsequently released into the portal circulation [13]. Conversion of absorbed folic acid into L-5-MTHF appears to occur predominantly in the liver, resulting in the appearance of unmetabolized folic acid in the circulation if the liver’s capacity is exceeded [13]. In infants, even the relatively low levels found in formula have led to a significant increase in the levels of unmetabolized folic acid in the plasma of formula fed infants [15].

Preclinical data suggest that unmetabolized folic acid may impair cellular folate metabolism e.g. in endothelial cells [36], likely due to the stronger affinity for folate binding proteins compared to L-5-MTHF [37]. Moreover, it has been suggested by in vitro and in vivo animal studies that unmetabolized folic acid might have adverse effects, but the evidence remains controversial [16]. It has been hypothesized that elevated levels of unmetabolized folic acid result in an increase in dihydrofolate, which then inhibits MTHFR as well as thymidylate synthase, thereby causing functional folate deficiency [7]. Preclinical data also indicate that unmetabolized folic acid reduces the transport of L-5-MTHF into endothelial cells, which might result in an impaired intracellular folate metabolism [36]. Moreover, data from adults indicate potential untoward effects on immune functions as indicated by reduced natural killer cell cytotoxicity [38, 39].

To turn folic acid into L-5-MTHF, the active folate form, several conversion steps are necessary and one of those is catalyzed by MTHFR [2]. While MTHFR deficiency is rare in humans [40], three common mutations of the MTHFR gene, namely C677T, A1298C, and T1317C, have been proposed for an association with various pathological conditions. The T1317C mutation appears to be a silent polymorphism [41] and is therefore not regarded as relevant.

The proportion of people that are homozygote for the MTHFR C677T mutation is thought to range from close to 0% in Sub-Saharan Africans to 32% in Mexicans [42, 43]. In Europe, prevalence ranging from 4% in Helsinki (Finland), to 26.4% in Campania (Italy) were reported [43]. In the case of the C677T polymorphism, MTHFR enzyme activity seems to be reduced by up to 50% in homozygotes [44]. The prevalence of the A1298C mutation in the MTHFR gene also seems to differ between ethnic groups: while non-Hispanic white in the U.S. showed a similar prevalence of homozygous carriers (~12%) as reported for Canadians, in Mexican Americans, it was ~20% and in non-Hispanic blacks just over 1% [45]. The reduction in MTHFR activity seems to be less for A1298C than for the C677T mutation (~70% of wild type) [46]. However, in vitro studies indicate a synergistic effect for the two mutations [46]. Consequently, infants homozygous for one or both polymorphisms would potentially benefit even more from the use of L-5-MTHF instead of folic acid in infant formula. Given the low number of infants with the 677TT genotype in our study population (Table 2), it might be prudent to conduct further studies in populations with a higher prevalence of this specific genotype as this might affect the bioavailability and consequently optimal level of L-5-MTHF.

Strengths of this study are the randomized controlled study design following good clinical practice standards, the inclusion of a large number of healthy infants, detailed and standardized assessment of growth, tolerance, adverse effects and biochemical markers. A limitation of this study is the lack of reliable information on energy intake from foods other than study formulae. However, being more stringent in collecting dietary intake data would have put a higher burden on the parents and caregiver, thereby increasing the risk of non-compliance and drop-outs.

## Conclusions

Infants who consumed an infant formula with L-5-MTHF did not show significant differences in growth and tolerance compared to infants fed the same formula with folic acid at equimolar doses. Furthermore, infants in the intervention group had significantly lower plasma levels of unmetabolized folic acid and significantly higher RCF concentrations. L-5-MTHF is the predominant folate form in breast milk and its addition to infant formulae did not raise any safety concerns in our study. Therefore, it appears prudent to allow the addition of L-5-MTHF as a folate source to infant and follow-on formula in equimolar doses to the currently recommended folic acid levels. Plasma of exclusively breastfed infants whose mothers do not have larger intakes of folic acid from supplements or fortified foods contain low levels of unmetabolized folic acid. Consequently, it appears prudent to provide folate sources to infant formula that do not lead to high levels of plasma unmetabolized folic acid, following the precautionary principle. L-5-MTHF is one possible safe approach to provide adequate folate without increasing the amount of unmetabolized folic acid.

## Supporting information

S1 Supplemental Material Study protocol. (PDF)

S2 Supplemental Material CONSORT 2010 checklist. (PDF)

S1 Table Formulation of infant formula powder for intervention and control group. (PDF)

S2 Table Baseline characteristics in the modified intention-to-treat population: Age, sex and MTHFR polymorphisms C677T (rs1801133) and A1289C (rs1801131). (PDF)

S3 Table Comparison of parameters of acceptability and tolerance of the intervention formula in the per-protocol population. (PDF)

S4 Table Summary of adverse events by category and group. (PDF)

S5 Table Summary of blood chemistry and hematology parameters. (PDF)

S6 Table Daily weight gain for the intervals between the visits for the modified intention-to-treat and the per-protocol population. (PDF)

S7 Table Fixed effects solution for the model for the gain in weight, recumbent length, head circumference and calorie intake in the modified intention-to-treat and per-protocol population. (PDF)

S8 Table Least square means for body weight, recumbent length, head circumference and calorie intake at visit 4 for the modified intention-to-treat and the per protocol population. (PDF)

S9 Table Least square means for body weight at visit 4 according to treatment-gender and -genotype interaction model for the modified intention-to-treat and the per protocol population. (PDF)

S1 Fig Consistency (a.), color (b.) and smelliness of stool at visits 1 and 4 in the per protocol population.
(TIF)
