# Correction: Suitability and safety of L-5-methyltetrahydrofolate as a folate source in infant formula: A randomized-controlled trial

**DOI:** 10.1371/journal.pone.0308350

**Published:** 2024-07-31

**Authors:** Barbara Troesch, Johann Demmelmair, Martina Gimpfl, Christina Hecht, Goran Lakovic, Robert Roehle, Ljilja Sipka, Branka Trisic, Milica Vusurovic, Rotraut Schoop, Sznezana Zdjelar, Berthold Koletzko, on behalf of the MEFOLIN Study Group

After publication of this article [1], the authors discovered that the infant’s ‘whole blood folate’ concentrations in [1] were incorrectly referred to as ‘red cell folate’ throughout the manuscript.

Here, the authors have provided additional information to clarify these issues: The authors measured whole blood folate by using the microbiological assay, and have corrected for the dilution factor only. Measuring red cell folate would additionally require corrections for individual values of plasma folate and hematocrit but this was not performed in [1].

The authors have provided the following correction: All instances of ‘red cell folate’ in the text should be replaced with ‘whole blood folate’.
