# Supplementary material for: Suitability and safety of L-5-methyltetrahydrofolate as a folate source in infant formula: A randomized-controlled trial
Source: PLoS One. 2019 Aug 19;14(8):e0216790. doi: 10.1371/journal.pone.0216790 (PMC6699731; doi:10.1371/journal.pone.0216790)
Supplement: S2 Supplemental Material — (PDF) [file pone.0216790.s002.pdf]

PROTOCOL FOR BIOMEDICAL RESEARCH PROJECTS INVOLVING HUMAN SUBJECTS

**TITLE**	**Suitability of an infant formula with L-5-Methyltetra-hydrofolate for the particular nutritional use in infants**
**PROTOCOL NUMBER**	2014-05-06-MTHF
**VERSION**	1.0
**DATE**	07.12.2014
**SPONSOR**	DSM Nutritional Products Ltd. (DNP)<br>Wurmisweg 576<br>CH-4303 Kaiseraugst<br>Switzerland

ROLES AND RESPONSIBILITIES

SPONSOR

**PROJECT MANAGER**	Dr. Stephanie Krammer-Lukas
Company	DSM Nutritional Products Ltd.
Department	HNH Innovation Project Management
Address	Wurmisweg 576, 241/425, 4303 Kaiseraugst, Switzerland
Phone	+41 61 815 8437
Fax	+41 61 815 8220
E-mail	Stephanie.krammer-lukas@dsm.com

**STUDY DIRECTOR**	Dr. Szabolcs Péter, MD, PhD
Company	DSM Nutritional Products Ltd.
Department	NIC-RD/HN
Address	Wurmisweg 576, 203.4/182, 4303 Kaiseraugst, Switzerland
Phone	+41 61 815 8966
Fax	+41 61 815 8050
E-mail	Szabolcs.peter@dsm.com

**CORPORATE SCIENTIST**	Dr. Peter Weber, MD, PhD
Company	DSM Nutritional Products Ltd.
Department	NSA
Address	Wurmisweg 576, 203/35, 4303 Kaiseraugst, Switzerland
Phone	+41 61 815 8135
Fax	+41 61 815 8740
E-mail	Peter.weber@dsm.com

PRINCIPAL INVESTIGATOR

**PRINCIPAL INVESTIGATOR**	Prof. Berthold Koletzko
Title, Function	Univ.- Professor
Institution	Dr. von Hauner Children’s Hospital, Ludwig-Maximilians-Univ. of Munich
Address	Lindwurmstr. 4, 80337 Munich, Germany
Phone	+49 895160 3967
Fax	+49 89 8396 4044
E-mail	Berthold.koletzko@med.lmu.de

CONTRACT RESEARCH ORGANISATION (ECRF, DATA MANAGEMENT, STATISTICS)

**CONTACT PERSON**	Dr. Olaf Bender
Title, Function	Project Coordinator
Address	KKS Charité, Augustenburger Platz 1,13353 Berlin, Germany
Phone	+49 30 450 553 864
Fax	+49 30 450 553 937
E-mail	olaf.bender@charite.de

CONTRACT RESEARCH ORGANISATION (MONITORING)

**CONTACT PERSON**	Ana Gašparović
Title, Function	Project Manager
Address	Sermon CRO, Donje Svetice 46 C/V, 10000 Zagreb, Croatia
Phone	+385 1 550 92 47
Fax	+385 1 23 34 616
E-mail	ana.gasparovic@sermoncro.com

ADDITIONAL PERSONS

**PRINCIPAL SITE INVESTIGATOR**	Prim. Dr. Milica Vusurovic
Institution	KBC "Dr Dragiša Mišović-Dedinje"
Department	Neonatology
Address	Heroja Milana Tepića 1, 11000 Belgrade, Serbia
Phone	+381 (0) 63 312 171
Fax	-
E-mail	milica.vusurovic@gmail.com

**LOCAL INVESTIGATOR**	Dr. Snezana Zdjelar
Institution	KBC "Dr Dragiša Mišović-Dedinje"
Department	Neonatology
Address	Heroja Milana Tepića 1, 11000 Belgrade, Serbia
Phone	+381 (0) 62 421 041
Fax	-
E-mail	nenazdjelar@gmail.com

**LOCAL INVESTIGATOR**	Dr. Liljana Sipka
Institution	KBC "Dr Dragiša Mišović-Dedinje"
Department	Neonatology
Address	Heroja Milana Tepića 1, 11000 Belgrade, Serbia
Phone	+381 (0) 65 813 7100
Fax	-
E-mail	lillia54@gmail.com

**LOCAL INVESTIGATOR**	Dr. Goran Lakovic
Institution	KBC "Dr Dragiša Mišović-Dedinje"
Department	Neonatology
Address	Heroja Milana Tepića 1, 11000 Belgrade, Serbia
Phone	+381 (0) 64 115 1509
Fax	-
E-mail	goranlakiboy@gmail.com

**STUDY NURSE**	Biljana Radic
Institution	Hipp GmbH und Co. Vertrieb KG
Department	Clinical Study Center, Belgrade
Address	Kneza Milosa 51, 11000 Belgrade, Serbia
Phone	+381 (0) 60 515 0520
Fax	+381 (0) 11 3612 944
E-mail	biljanardc@gmail.com

**LOCAL PROJECT COORDINATOR**	Dr. Branka Triŝic
Company	Hipp GmbH und Co. Vertrieb KG
Department	Clinical Study Center, Belgrade
Address	Kneza Milosa 51, Belgrade, Serbia
Phone	+381 (0)11 3612 944
Fax	+381 (0)11 3612 944
E-mail	hipp@technicom.net

**LOCAL STUDY MANAGER**	Radina-Nina Lukic
Company	Hipp GmbH und Co. Vertrieb KG
Department	Clinical Study Center, Belgrade
Address	Kneza Milosa 51, Belgrade, Serbia
Phone	+381 (0) 11 3612 944, +381 (0) 60 5150 530
Fax	+381 (0)11 3612 944
E-mail	ninalukic8@gmail.com

**LOCAL LABORATORY**	Drina Topalov
Company	Institute for Laboratory Diagnostics “Konzilijum“
Address	Svetog Save 28a, 11000 Belgrade, Serbia
Phone	+381 (0) 63 555 866
Fax	+381 (0) 11 243 0804
E-mail	drina.topalov@konzilijum.rs

**FOLATE LABORATORY**	Gry Kvalheim
Company	Bevital AS
Address	Jonas Lies vei 87, 5021 Bergen, Norway
Phone	+47 55974694, +47 90874191
Fax	-
E-mail	gry@bevital.no

**GENETIC LABORATORY**	Dr. Melanie Waldenberger
Company	Research Unit of Molecular Epidemiology, Helmholtz Zentrum München
Address	Ingolstädter Landstr. 1, D-85764 Neuherberg, Germany
Phone	+49 89 3187-1270
Fax	+49 89 3187-4567
E-mail	waldenberger@helmholtz-muenchen.de

**BIOSTATISTICIAN**	Dr. Rotraut Schoop
Company	DSM Nutritional Products Ltd.
Address	Wurmisweg 576, 203.4/188, 4303 Kaiseraugst, Switzerland
Phone	+41 61 815 7523
E-mail	rotraut.schoop@dsm.com

**BIOSTATISTICIAN**		Alexander Krannich
	Institution	Koordinierungszentrum für Klinische Studien der Charité<br>KKS Charité
	Address	Augustenburger Platz 1, 13353 Berlin, Germany
	Phone	+49 30 450 553 884
	Fax	+49 30 450 553 937
	E-mail	alexander.krannich@charite.de

**LOCAL PROJECT COORDINATOR**	Dr. Branka Triŝic
Signature	
Place and date	
**LOCAL LABORATORY**	Drina Topalov
Signature	
Place and date	
**BIOSTATISTICIAN**	Dr. Rotraut Schoop
Signature	
Place and date	
**BIOSTATISTICIAN**	Alexander Krannich
Signature	
Place and date	
**DSM INNOVATION PROJECT MANAGER**	Dr. Stephanie Krammer-Lukas
Signature	
Place and date	

Principal Investigator's Signature

I have carefully read this protocol entitled "Suitability of an infant formula with L 5 Methyltetra hydrofolate for the particular nutritional use in infants" and agree that this study is to be conducted in accordance with Good Clinical Practice (GCP), the ethical principles that have their origin in the Declaration of Helsinki and further described in International Conference on Harmonisation (ICH GCP) E6. Title 21 of the Code of Federal Regulations §§ 50, 56, and 312 and European Union Directive 2001/20/EC for Clinical Trials and 2005/28/EC for Good Clinical Practice

In that it contains all the necessary information required to conduct the study I agree to conduct this study as outlined in the protocol.

I understand that this study will not be initiated without approval of the appropriate institutional review board/independent ethics committee, and that all administrative requirements of the governing body of the institution will be complied with fully

Informed written consent will be obtained from all subjects/patients in accordance with the institutional requirements as set forth in the terms of the declaration of Helsinki as specified in the good clinical practice consolidation guidance issued by the International Conference on Harmonization.

I will enrol subjects/patients who meet the protocol inclusion criteria.

I understand that my signature on the completed case report form(s) indicates that I have carefully reviewed each page and accept full responsibility for the contents thereof

I understand that the information presented in this study protocol is confidential, and I hereby assure that no information based on the conduct of the study will be released without prior consent from DNP unless the requirement is superseded by the appropriate regulatory authority.

PRINCIPAL INVESTIGATOR Prof. Berthold Koletzko

Signature

Place and date München, 11. December 2014

SPONSOR'S APPROVALS AND SIGNATURES

STUDY DIRECTOR Dr. Szabolcs Peter

Signature i.A. Kell

Place and date KAISERAUGST, 11.12.2014

CORPORATE SCIENTIST Dr. Peter Weber

Signature

Place and date Kaiseraugst Dec 15, 2014

ADDITIONAL PERSONS

PRINCIPAL SITE INVESTIGATOR Prim. Dr. Milica Vusurovic 17.12.2014.

Signature

Place and date

**LOCAL PROJECT COORDINATOR**	Dr. Branka Trišic
Signature	[illegible]
Place and date	[illegible], 16.12.2014
**LOCAL LABORATORY**	Drina Topalov
Signature	Drina Topalov
Place and date	BEOGRAD, 23.12.2014
**BIOSTATISTICIAN**	Dr. Rotraut Schoop
Signature	Rotraut Schoop
Place and date	Kaiseraugst, 11.12.2014
**BIOSTATISTICIAN**	Alexander Krannich
Signature	[illegible]
Place and date	Berlin, 12.12.2014
**DSM INNOVATION PROJECT MANAGER**	Dr. Stephanie Krammer-Lukas
Signature	[illegible]
Place and date	Kaiseraugst, 15.12.2014

SYNOPSIS

TITLE	**Suitability of an infant formula with L-5-Methyltetra-hydrofolate for the particular nutritional use in infants**		
STUDY ACRONYM	MEFOLIN		
PROTOCOL NUMBER	2014-05-06-MTHF	EUDRACT NUMBER (IF APPLICABLE)	N/A
VERSION	1.0	DATE	07/12/2014
SPONSOR	DSM Nutritional Products Ltd.<br>Wurmisweg 576, 4303 Kaiseraugst, Switzerland		
PLANNED START OF STUDY	Q1 2015	ESTIMATED STUDY DURATION	36 months
PRINCIPAL INVESTIGATOR<br><br><br><br><br><br>PRINCIPAL SITE INVESTIGATOR<br><br><br><br><br><br>LOCAL INVESTIGATORS	Prof. Dr. Berthold Koletzko<br>Div. Metabolic and Nutritional Medicine,<br>Dr. von Hauner Children's Hospital,<br>Univ. of Munich Medical Centre - Klinikum d. Univ. München,<br>Lindwurmstr. 4, D-80337 Munich, Germany<br><br>Prim. Dr. Milica Vusurovic<br>KBC "Dr Dragiša Mišović-Dedinje"<br>Department of Neonatology<br>Heroja Milana Tepića 1<br>11000 Belgrade, Serbia<br><br>Dr. Snezana Zdjelar, Dr. Liljana Sipka, Dr. Goran Lakovic<br>KBC "Dr Dragiša Mišović-Dedinje"<br>Department of Neonatology<br>Heroja Milana Tepića 1<br>11000 Belgrade, Serbia		
STUDY SITE	Recruitment:<br>KBC "Dr Dragiša Mišović-Dedinje"<br>Heroja Milana Tepića 1<br>11000 Belgrade, Serbia<br><br>Enrollment and clinical examinations:<br>HiPP Clinical Study Center<br>Kneza Milosa 51<br>Belgrade, Serbia		
APPLICATION FIELD	Infant growth and development		
PRIMARY OBJECTIVE	Primary objective is to show equivalence of an infant formula containing L-5-Methyltetrahydrofolate ("MTHF") compared to a standard infant formula containing folic acid on weight gain of healthy term infants receiving these infant formulae exclusively during the first 4 months of life.		

**SECONDARY OBJECTIVES**	Secondary objectives are<br><br>– to show that parameters of growth and development (body length and fronto-occipital head circumference) are clinically equivalent between the groups and comparable to a reference group of breast fed infants;<br>– to show that energy intake in both infant formula groups is clinically equivalent;<br>– to show, that tolerability of both infant formulae is good, as assessed by 3-day diary.
**EXPLORATORY OBJECTIVES**	Exploratory objectives are<br><br>– to show that folic acid status (plasma levels of 5-MTHF, FA, fTHF, hmTHF, pABG, apABG to assess current folate supply, and red cell total folate [RCF] to assess longer term folate supply) in blood samples of infants receiving the intervention infant formula instead of the control formula is more comparable in intervention than control to that of a reference group of fully breastfed infants;<br>– to assess the frequency of the homozygous genotypes of common single nucleotide polymorphisms in methylentetrahydrofolate-reductase (MTHFR) mutations C677T and A1289C and their association with the folate status in infants (blood cells will be stored for eventual later epigenetic analyses);<br>– to assess the potential long-term impact of the intervention vs. control, compared to reference, on growth parameters, haematology, and adverse events at age of 1 year;<br>– to assess the plasma levels of 5-MTHF, FA, fTHF, hmTHF, pABG, apABG and blood hematology of the breast-feeding mothers;<br>– to assess breast milk levels of 5-MTHF, FA, fTHF, hmTHF, pABG, apABG;<br>– to assess maternal intake of folic acid and MTHF.
**STUDY DESIGN**	Mono-centric, randomized, double-blind, parallel-group, controlled prospective intervention study in healthy infants receiving one of the following preparations:<br><br><u>Group 1:</u> Infant formula including folic acid within the range stipulated by European legislation on infant formula (control group, n=120)<br><u>Group 2:</u> Infant formula including MTHF instead of folic acid (intervention group, n=120)<br><u>Group 3:</u> Breast milk (reference group, n=120)
**STUDY POPULATION**	Male or female healthy infants born at full-term
**SAMPLE SIZE**	Total number of subjects: 360 (n=120 in control group, n=120 in intervention group, n=120 in reference group)
**NUMBER OF SITES**	One (1) site

**INVESTIGATIONAL PRODUCT**	– Control product is an infant formula following current standards as laid down in the European legislation for infant formulae (from the year 2006), which contains 10 µg folic acid per 100 ml reconstituted infant formula<br>– The intervention product is an infant formula with similar compositional characteristics as the control product, except for including 13.0 µg MTHF (calcium salt of (6S)-5-Methyltetrahydrofolic acid) per 100 ml reconstituted infant formula (instead of folic acid)<br><br>The control product and the intervention product will be provided by Hipp and are as similar as technically feasible in all compositional aspects except for the source of folate.		
**REFERENCE**	– Breast milk		
**DOSAGE AND REGIME**	Ad libitum according to recommendations of Hipp	**DURATION OF SUPPLEMENTATION**	From inclusion until the age of 16 weeks of life
**OUTCOME PARAMETERS**	***Primary outcome measure:***<br>Infant weight gain in gram per day between age of four weeks and 16 weeks (total weight gain between V1 and V4 divided by the number of days between those visits)<br><br>***Secondary outcome measures (between week 4-16):***<br>– Body length gain (in cm/day) and fronto-occipital head circumference gain (in cm/day)<br>– Dietary intake of study formula and non-study formula, additional liquids and solids (expressed as kcal per day and as kcal per kg body weight per day) as assessed by 3-day diary<br>– Feeding related behaviour (acceptance, formula tolerance, spitting, vomiting, crying, sleeping time per day) based on 3-day diary; stool characteristics (color and consistency) assessed by the Bristol scale criteria included in the 3-day diary		
**ADDITIONAL PARAMETERS**	– Plasma levels of 5-MTHF, FA, fTHF, hmTHF, pABG, apABG and red cell total folate levels (RCF) at Baseline Visit and Visit 4<br>– Frequency of the homozygous C677T and A1289C genotypes of MTHFR in infants at Baseline Visit (blood cells will be stored for eventual later epigenetic analyses)<br>– Growth parameters (weight, length, head circumference) and haematology (haemoglobin, HCT, MCV) at age of 1 year<br>– Plasma levels of 5-MTHF, FA, fTHF, hmTHF, pABG, apABG and blood hematology of the breast-feeding mothers at visit 2<br>– Breast milk levels of 5-MTHF, FA, fTHF, hmTHF, pABG, apABG at Visit 2<br>– Maternal intake of folic acid and MTHF based on questionnaire at Visit 2		

**SAFETY PARAMETERS**	–Assessment of health and intermittent clinical conditions at visits 1 to 4<br>–Clinical safety panel, including hematology (haemoglobin, HCT, MCV) and serum biochemistry (creatinine and ALAT)<br>–AE/SAE reporting according to DNP SOPs<br>–Follow up visits at the age of 12 months and determination of weight, length, head circumference, haemoglobin, HCT, MCV, and occurrence of adverse events
**STUDY PROCEDURES**	The study will be performed in two phases:<br>1. Main study: Database will be locked after 4 months of age of the last infant and availability of all laboratory results, statistical analyses will be performed and main study report will be prepared<br>2. Follow-up study: While the study centre remains blinded, additional visit at the age of 12 month will be performed and additional report for the follow-up will be prepared<br><br>**Study visits are planned as follows:**<br><br>– Baseline Visit: between day 1 and day 27 of life<br>– Visit 1: at the age of 4 weeks<br>– Visit 2: at the age of 8 weeks<br>– Visit 3: at the age of 12 weeks<br>– Visit 4: at the age of 16 weeks<br>– Follow-up visit: at age of 12 months<br><br>**Recruitment period:** Study doctors of the KBC hospital will get in contact with the mothers on the maternity ward after the clinical staff has determined whether parents have decided to breastfeed or formula feed. Care will be taken that any potential interference with breastfeeding is avoided. The study personnel will inform parents about the study. Parents who are willing to participate in the study are checked for inclusion and exclusion criteria. Personal details of the parents are then forwarded to the Clinical Study Center, Belgrade.<br><br>**Enrollment period:** Manager of the Study Center get in contact with parents after they left the hospital. Parents who are still interested in the study are invited for a baseline visit at the Study Center.<br><br>**Visits during study:** All visits take place at the Study Center. Study doctors attend the visits to conduct the clinical examinations.<br><br>**Baseline Visit between day 1 and day 27 of life**<br>Recruitment takes place from birth until the 27th postnatal day. All infants who meet the inclusion / do not meet the exclusion criteria can be included in the study following a written declaration of consent given by their parents (persons having custody over infant).

	If the parents decide not to breastfeed their child such children are randomly assigned to one of the two intervention arms based on a randomization plan.<br>Upon the written declaration of consent given by the parents (persons having custody over infant), fully breastfed infants (>90 % of energy intake from breast milk, not more than 1 bottle per day intake from other sources) are admitted to the study and added to the reference group.<br><br>At the Baseline Visit a medical examination and baseline anthropometric data assessment will be performed.<br><br>The observation of the infant's growth that lasts at least 3 months starts upon recruitment and no later than the age of 4 weeks (28 days) and continues until the child is aged 16 weeks (4 months). That period is followed by the follow-up period – without any predefined intervention – that starts at age 16 weeks (4 months) and lasts until the child's first birthday.<br><br>Blood samples of infants will be collected at Baseline Visit to analyze 5-MTHF, FA, fTHF, hmTHF, pABG, apABG in plasma, total RCF and for determination of serum creatinine and ALAT, as well as haemoglobin, HCT and MCV. Blood cells will be stored for genotyping and eventual later epigenetic analyses.<br><br>**Visit 1 to Visit 4** :<br>At 4 weeks (V1), 8 weeks (V2), 12 weeks (V3) and 16 weeks (V4) of age, infants will participate in a clinical study visit again. Medical examination will be performed and anthropometric data will be assessed.<br><br>Prior to each visit (V1-V4) parents are asked to fill in 3-day diaries. These records include dietary intake and formula tolerance parameters.<br><br>At Visit 2 a blood sample from the breast-feeding mothers will be collected as well to determine the plasma levels of 5-MTHF, FA, fTHF, hmTHF, pABG, apABG and blood hematology. In addition, a manually expressed sample of breast milk will be collected to determine levels of 5-MTHF, FA, fTHF, hmTHF, pABG, apABG in human milk. Visit 2 also includes assessment of supplement intake by the mother.<br><br>Blood samples of infants will be collected at Visit 4 to analyze 5-MTHF, FA, fTHF, hmTHF, pABG, apABG in plasma, total RCF and for determination of serum creatinine and ALAT, as well as haemoglobin, HCT and MCV.<br><br>**<u>Follow-up visit:</u>** At an age of 12 months a follow-up visit is performed with a medical examination, determination of anthropometric data (weight, length, head circumference) and occurrence of adverse events. A blood sample will be drawn from the toddler (for determination of haemoglobin, HCT and MCV).

SUBJECT SELECTION CRITERIA	**Inclusion criteria**<br>- Written informed consent (by parents, caregiver)<br>- Healthy male or female infants <28 days of life<br>- Gestational age at delivery ≥37 and ≤42 weeks<br>- Birth weight between 2500 – 4500g<br>- Parents / Caregivers are able to speak Serbian language<br>**Exclusion criteria**<br>- Serious acquired or congenital diseases that is expected to interfere with normal feeding or growth<br>- Feeding of more than 10% of energy (1 bottle/day) from sources other than the formula (or breast milk in the reference group) at inclusion<br>- Participation in another clinical study<br>- Mothers with diabetes mellitus (including GDM)<br>- Reason to presume that the parents are unable to meet the study plan requirements<br>- Diseases of the mother which have an effect on the child's gastro-intestinal tract/ability to be fed<br>- Major abnormalities in hematological parameters<br>- Major abnormalities in hepatic, renal or metabolic functions<br>- Use of medication and vitamin supplements except vitamin K or D supplementation or vaccination<br>- Mother follows a vegan diet
STATISTICAL CONSIDERATIONS	<u>Research question:</u> Is a modified infant formula containing MTHF suited to ensure normal growth and development of healthy children during the first 4 months of life (in comparison to a standard infant formula containing folic acid)?<br><br><u>Primary target variable – hypothesis (H_0) to be rejected:</u><br>The mean daily weight gain in the intervention group (MTHF group) is **either** at least 3.5 grams per day **lower** than the mean daily weight gain in the control group (standard formula containing folic acid) **OR** at least 3.5 grams per day **higher** than in the control group<br><br>Hypothesis H_A: The mean daily weight gain in the intervention group is less than 3.5 grams per day lower than the mean daily weight gain in the control group **AND** less than 3.5 grams per day higher than in the control group.<br>MTHF infant formula is suitable for the particular nutritional use by infants from birth onward.<br><br>**<u>Necessary sample size:</u>**<br>• According to the report of the EC Scientific Committee on Food 2003, "growth studies should be designed to have a power to detect a difference in weight gain equal to 0.5 standard deviations". In analogy to this recommendation, we consider the average daily weight gain of infants in the intervention group equivalent to the average daily weight gain of infants in the control group, if it is within the boundaries of +/- 0.5 standard deviations.

- Based on previous observation in Serbian infants (Fleddermann, Demmelmair et al. 2013), we estimate that the infants gain about 30 g weight per day with a standard deviation of about 7 g.
- We do not expect a difference in average daily weight gain between the group receiving intervention formula and the group receiving standard formula. The standard deviation of the group receiving infant formula with L-5-MTHF is supposed to be the same as in the control group, and comparable to the growth observed in the reference group.
- Using these assumptions and requiring a power of 80% and a type I error rate of 2.5%, sample size was calculated using Julious *(Sample Sizes for Clinical Trials, CRC Press 2010, Formula (5.10), pg. 85)*, for equivalence clinical trials with normal data in the special case of no treatment difference, resulting in group sample sizes of 84 analyzable infants per group. Since we expect a drop-out proportion of approximately 30 percent – a true loss to follow up of about 10-15% and another 15 % which will switch formula due to preference changes – 120 infants will have to be recruited per formula group.
- The group of breast fed infants serves as a reference only. No direct statistical comparison with the control or intervention group is planned.

Analysis populations:

- The primary endpoint will be analyzed using the modified Intent To Treat (ITT) population and the Per Protocol (PP) population. The ITT population is defined as all subjects who completed V1 after initiation of treatment. It is planned that 120 subjects in the formula groups, respectively, and 120 in the reference group should complete V1. Therefore, dropouts between Baseline Visit and V1 should be replaced (screening failure). The PP population is defined as all subjects that completed the 16 (V4) weeks study without any major protocol violation.

Statistical analysis of primary outcome body weight gain:

- The primary outcome is the daily weight gain in grams between day 28 and day 112 after birth. The primary outcome variable "weight gain per day" will be calculated by subtracting weight obtained at the Visit 1 (28 days of age) from the weight obtained at Visit 4 (at 112 days of age) divided by the number of days between those visits.
- The primary objective is to show equivalence of the two arms, intervention and control. For this, a TOST (two one-sided test) procedure will be performed, taking the cut-off level of statistical significance at 0.025. Because of the duality of test procedure and corresponding confidence interval equivalence of

	interventional nutrition regime can be proven by means of the interval boundaries. If the 95%-confidence interval for the difference of the mean daily weight gain of intervention and control arm is within the interval (-3.5 grams, 3.5 grams), H_0 will be rejected and equivalence will be concluded.<br>• The confidence interval for the difference of the mean daily weight gain will be calculated using a linear model on body weight with treatment, time in days and the time-treatment interaction as fixed effects, birth weight and sex as adjusting covariates and subject as random effect.<br><br>Further Analyses<br>• Data will be displayed in absolute values and as mean difference to Visit 1 with CI and SD by treatment group.<br>• In order to evaluate relative efficacy of the study formulas and breast milk, significant difference between groups for folate status and weight gain will be evaluated. Equivalence testing will be done for major parameters where an adequate equivalence margin can be appropriately defined for the preparation. The relevant margins will be defined before unblinding and initiation of data analysis. The same adjusting covariates as in the primary analysis will be used.
**ETHICAL, LEGAL AND REGULATORY REQUIREMENTS**	– Ethical approval is required from the site's IRB/EC.<br>– Study needs to be conducted according to local requirements.<br>– Study has to be conducted according to the principles of GCP and the Declaration of Helsinki.<br>– The study will be monitored by Sermon CRO.<br>– According to the ICH E9 Guideline on Statistical Principles for Clinical Trials, an Equivalence Trial is: "A trial with the primary objective of showing that the response to two or more treatments differs by an amount which is clinically unimportant. This is usually demonstrated by showing that the true treatment difference is likely to lie between a lower and an upper equivalence margin of clinically acceptable differences." Therefore this study is considered as an equivalence trial.<br>– This study is part of a dossier to be sent to the European Commission for admission of adding L-5-Methyltetrahydrofolate to infant and follow on formula. L-5-MTHF is the predominant form of folate found in human breast milk. The supplementation of MTHF (instead of folic acid) to infants fed with infant formula may help to increase L-5-MTHF and reduce unmetabolized folic acid plasma levels in this population. Thus, the plasma MTHF levels of bottle fed infants may become more similar to those of the breast fed population.

STUDY OVERVIEW

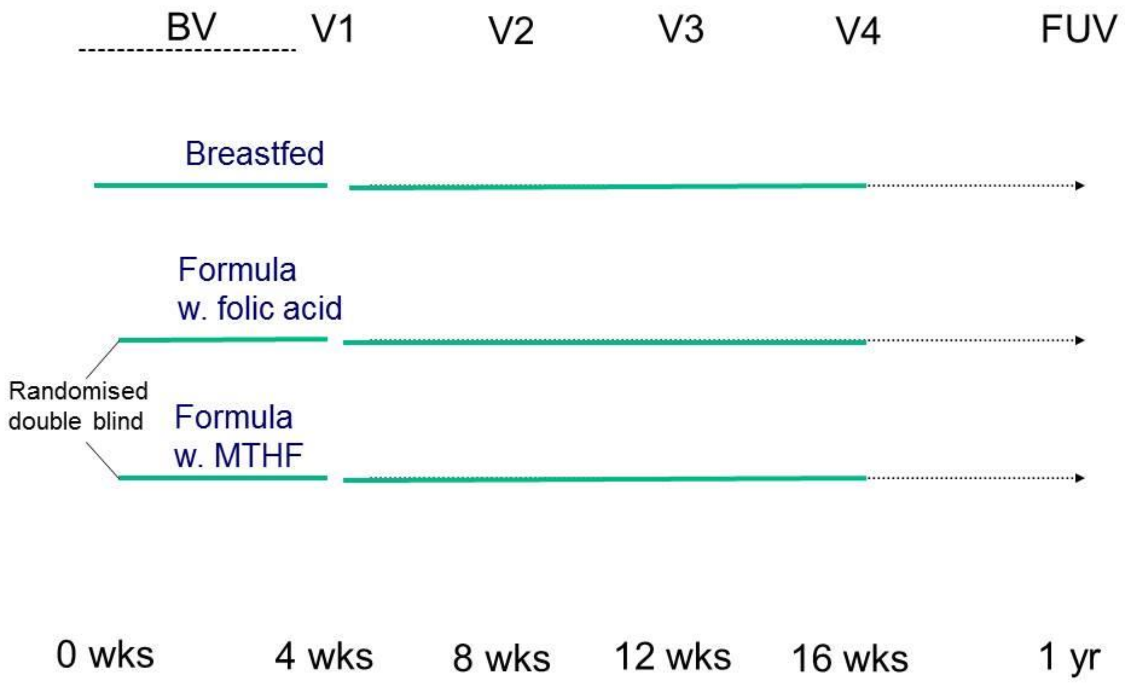

SCHEDULED ASSESSMENTS:

	Assessment periods					
Visits	**Baseline Visit**	**V1**	**V2**	**V3**	**V4**	**Follow-up visit**
Time	Between Day 1 and Day 27 of life	At the age of 4 weeks (±3 days)	At the age of 8 weeks (±3 days)	At the age of 12 weeks (±3 days)	At the age of 16 weeks (±3 days)	At the age of 12 months (±7 days)
**Assessments / Procedures**						
Inclusion/exclusion criteria	X					
Informed Consent	X					
Randomization	X					
Medical examination	X	X	X	X	X	X
Anthropometric data (weight, length, head circumference)	X	X	X	X	X	X
Blood sampling (plasma levels of 5-MTHF, FA, fTHF, hmTHF, pABG, apABG, total RCF, genotype at Baseline Visit)	X				X	
3-day diary (incl. Bristol scale) prior to each visit		X	X	X	X	
Questionnaire for the assessment of maternal supplement intake (folic acid, MTHF)			X			
Maternal blood sampling from the breast-feeding mothers (plasma levels of 5-MTHF, FA, fTHF, hmTHF, pABG, apABG, hematology)			X			
Breast milk sampling (levels of 5-MTHF, FA, fTHF, hmTHF, pABG, apABG)			X			
IP accountability	X	X	X	X	X	
**Safety**						
Adverse event reporting	\---/					
Assessment of health and intermittent clinical conditions		X	X	X	X	
Serum biochemistry (creatinine, ALAT)	X				X	
Hematology (haemoglobin, HCT, MCV)	X				X	X

ABBREVIATIONS

AE	Adverse event
ALAT	Alanine aminotransferase
APABG	Acetamidobenzoylglutamate
AR	Adverse reaction
CF	Control formula
CI	Confidence interval
CRF	Case report form
eCRF	Electronic case report form
CRO	Contract research organization
DNA	Deoxyribonucleic acid
DNP	DSM Nutritional Products Ltd.
DM	Data management
e.g.	For example
FA	Folic acid
FAO	Food and Agriculture Organization of the United Nations
fTHF	5-Formyl-tetrahydrofolate
FUV	Follow-up visit
GCP	Good clinical practice
GDM	Gestational diabetes mellitus
HCT	Haematocrit
hmTHF	4-Alfa-hydroxy-5-methyl-THF
IEC	Independent ethics committee
IF	Intervention formula
IRB	institutional review board
ISF	Investigator site file
ITT	Intent to treat population
MCV	Mean corpuscular volume
MTHF	L-5-Methyltetrahydrofolate
MTHFR	Methylentetrahydrofolate-reductase
PABG	Para-aminobenzoylglutamate
PGA	Pteroyl glutamic acid
PI	Principal investigator
PP	Per protocol population
PSI	Principal site investigator
RCF	Red cell folate
RDE	Remote data entry
SAE	Serious adverse event
SAM	S-adenosyl methionine
SCF	Scientific Committee on Food
SD	Standard deviation
SOP	Standard operating procedure
SUSAR	Suspected unexpected serious adverse reaction
TMF	Trial master file
TOST	Two one-sided test
WHO	World Health Organization

TABLE OF CONTENTS

1 INTRODUCTION 22

1.1 Background information 22

1.2 Investigational product 25

1.3 Rationale for conducting the biomedical research project 25

2 OBJECTIVES 25

2.1 Primary objective and endpoint variable 25
2.1.1 Primary objective 25
2.1.2 Primary endpoint variable 26

2.2 Secondary and exploratory objectives and endpoint variables 26
2.2.1 Secondary objectives 26
2.2.2 Exploratory objectives 26
2.2.3 Secondary endpoint variables (between week 4-16): 27
2.2.4 Additional parameters 27

2.3 Safety objectives and endpoint variables 27
2.3.1 Safety objective(s) 27
2.3.2 Safety endpoint variable(s) 27

3 SITE AND SUBJECTS 27

3.1 Number of subjects 27

3.2 Number of sites 28

3.3 Biomedical research project population 28
3.3.1 Inclusion criteria 28
3.3.2 Exclusion criteria 28

4 INVESTIGATIONAL PRODUCT AND SUPPLEMENTATION REGIMEN 29

4.1 Investigational product 29
4.1.1 Investigational product name and formulation 29
4.1.2 Control product name and formulation 29
4.1.3 Packaging and labelling 29
4.1.4 Handling and storage conditions 30

4.2 Supplementation regimen 31
4.2.1 Rationale for dose selection 31
4.2.2 Dosage regimen and dose adjustment 31
4.2.3 Route of administration 31
4.2.4 Supplementation duration 31

4.3 Dispensing and accountability 31

4.4 Compliance 32

4.5 Protocol Deviations/Violations 32

4.6 Warnings and precautions 32

4.7 Concomitant treatments/supplements and restrictions 33

5 STUDY DESIGN AND SUBJECT ASSIGNMENT 33

5.1 Study design 33

5.2 Duration of biomedical research project 33
5.2.1 Duration of biomedical research project 33
5.2.2 Duration of biomedical research project per subject 33

5.3 Randomization, blinding and treatment allocation 33

5.4 Unblinding procedure 34

6 STUDY PROCEDURES 34

6.1 Recruitment procedures 34

6.2 Schedule of assessments 35

6.3 Screening and eligibility 37
6.3.1 Obtaining informed consent 37
6.3.2 Baseline Visit between Day 1 and Day 27 of life 37
6.3.3 Assessment of eligibility and randomization procedure 37

6.4 Intervention phase 37
6.4.1 Visit 1 at the age of 4 weeks 37
6.4.2 Visit 2 at the age of 8 weeks 37
6.4.3 Visit 3 at the age of 12 weeks 38
6.4.4 Visit 4 at the age of 16 weeks 38

6.5 Follow-up phase 38
6.5.1 Follow-up visit at the age of 12 months 38

7 MEASUREMENTS AND ASSESSMENTS 38

7.1 Blood and milk samples 38
7.1.1 Blood sample collection 39
7.1.2 Milk sample collection 39
7.1.3 Blood sample storage and shipment 39
7.1.4 Milk sample storage and shipment 40
7.1.5 Blood sample assessment 40
7.1.6 Milk sample assessment 40

7.2 DNA sample 41
7.2.1 DNA sample collection 41
7.2.2 DNA sample storage, shipment and genotyping 41

7.3 Other assessments 41
7.3.1 Anthropometric measurements 41
7.3.2 Questionnaires 42

8 EARLY SUBJECT WITHDRAWAL 42

9 STATISTICAL CONSIDERATIONS 43

10 SAFETY 44

10.1 Definitions and Standards 44

10.2 Adverse event assessment 45

10.3 Reporting procedures 46
10.3.1 Responsibilities 47

11 ETHICAL CONSIDERATIONS 48
11.1 Local Regulations and Declaration of Helsinki 48
11.2 DNP's GCP Management Directive 49
11.3 Informed Consent 50
11.4 Ethical Committee approval 50
11.4.1 Risk-benefit assessment 50
11.5 Confidentiality 51
11.5.1 Data 51
11.5.2 Subject Anonymity 51
11.6 Subjects' compensation / remuneration 51
11.7 Registration of study in a public clinical trial database 51

12 STUDY DOCUMENTATION AND RECORD KEEPING 51
12.1 Protocol amendments 51
12.2 Investigator site file 51
12.3 Case Report Form 52
12.3.1 Electronic Data Capture 52
12.4 Data management 53
12.5 Source document and source data verification 53
12.6 Insurance 53
12.7 Monitoring 53
12.8 Quality assurance and quality control 54
12.9 Final Study Report 54
12.10 Archiving 54
12.11 Publication 55

13 CONDITIONS FOR TERMINATING THE STUDY 55

14 REFERENCES 56

APPENDICES 60

1 INTRODUCTION

1.1 Background information

Folate is a general term for compounds that have a common vitamin activity and includes the synthetic form of the vitamin folic acid (pteroyl glutamic acid, PGA) and a wide variety of derivatives (Scientific Committee on Food 2003). Folic acid, the synthetic form, is not present in nature. Folate from naturally occurring food sources exhibits variable and often incomplete bioavailability, folic acid has higher bioavailability relative to food folate. Pteroyl glutamic acid consists of three subunits, pteridine, *p*-aminobenzoic acid and glutamic acid, and has a molecular mass of 441. It is odourless and tasteless, and is virtually insoluble in water, alcohol, acetone, chloroform, and ether; but is soluble in hydrochloric acid and sulphuric acids. The disodium salt is freely soluble. Aqueous solutions are heat sensitive and decompose rapidly in the presence of light. The derivatives of PGA are predominantly present in the body and foods as reduced folates, with variable numbers of glutamate residues.

Folate is functionally important for one carbon transfer reactions, including those involved in amino acid metabolism (serine/glycine and homocysteine/methionine interconversion), purine and pyrimidine synthesis, and the formation of the primary methylating agent, S-adenosyl methionine (SAM).

Folate deficiency is associated with a reduction in *de novo* DNA biosynthesis and thus impairment of cellular replication: recognised haematologically as macrocytic anaemia. Poor folate status may lead to hyperhomocysteinaemia, which has been associated with and increased risk of vascular disease. Folate is effectively reabsorbed in the kidney proximal tubules and little or no folate is lost in the urine at normal folate intakes.

In human milk, a wide range of folate concentrations has been reported, from 24-141 µg/L (3.8 to 20.9 µg/100 kcal) (Scientific Committee on Food 2003). Salmenpera *et al.* (Salmenpera, Perheentupa et al. 1986) followed 200 infants of healthy well-nourished mothers, all of whom were given a folate supplement (0.1 mg/day). In infants fed a formula containing 35 µg/L (5 µg/100 kcal, FAO/WHO recommended level), 69 to 94% had plasma folate levels below the lowest levels for the breast-fed infants. Those infants weaned earliest had the lowest haemoglobin concentrations and highest MCV at 9 months of age. The authors concluded that infants fed the recommended level were at risk of developing folate deficiency.

The Scientific Committee on Food of the European Commission in 2003 recommended a folic acid content in infant formulae and follow-on formulae of 10-30 µg/100 kcal (Scientific Committee on Food 2003), whereas the current European legislation on infant formulae and follow-on formulae sets a required content of folic acid of 10-50 µg/100 kcal (European-Commission 2006). However, the use of L-5-Methyltetra-hydrofolate on infant formulae and follow-on formulae is not foreseen either in the SCF report (Scientific Committee on Food 2003) or in the current European legislation (European-Commission 2006).

In relation to the inclusion of folic acid or 5-MTHF into an infant formula some aspects of folate intake and metabolism are of relevance. Observational and interventional studies have shown the importance of folate status for several diseases and health risks

including cardiovascular diseases, malignant diseases and neural tube defects (Blom and Smulders 2011, Kennedy, Stern et al. 2012). The relevance of folate status during the perinatal period is suggested by high foetal requirements, active placental transfer (Fekete, Berti et al. 2010) and high folate levels in cord blood and infantile blood samples collected at the age of 6 months compared to samples collected at later ages (Hay, Johnston et al. 2008). As with other nutrients, it is well conceivable that perinatal folate status influences long term health and disease risk of the offspring (Dominguez-Salas, Cox et al. 2012).

While natural folate in the human diet occurs in the polyglutamated form, the dietary folate for infants is provided by breast milk or infant formula, which both contain predominantly short chain folates or folate monoglutamate (Kim, Yang et al. 2004). It is well accepted that folic acid from supplements or fortified food has a higher bioavailability than natural folate, but a distinction between the bioavailability of folic acid and reduced 5-MTHF seems more difficult. Similar short term bioavailability had been reported (Pentieva, McNulty et al. 2004), but more recently short term studies have reported superior efficiency of 5-MTHF to increase plasma levels (Prinz-Langenohl, Bramswig et al. 2009, Ohrvik, Buttner et al. 2010). Accordingly longer term studies, considering red cell folate levels showed higher folate levels after 24 weeks or 16 weeks, respectively, of equimolar supplementation with 5-MTHF than with folic acid (Houghton, Sherwood et al. 2006, Lamers, Prinz-Langenohl et al. 2006). This agrees with the observation in folate depleted rats, which were fed milk diets either enriched with of 5-MTHF or folic acid for 4 weeks, leading to higher liver and red blood cell folate levels in the 5-MTHF group (Perez-Conesa, Haro-Vicente et al. 2009).

A mechanistic explanation of the difference between the folate forms may be provided by a recent tracer experiment in patients with a transjugular intrahepatic portosystemic shunts enabling sampling of the portal vein (Patanwala, King et al. 2014). In the crossover study physiological doses of stable isotope labelled 5-Formyl tetrahydrofolate and folic acid were orally applied and subsequently portal vein and a peripheral vein were sampled. During the 85 min observation period about twice as much 5-Methyltetrahydrofolate was detected after giving the reduced form compared to the folic acid application in the portal vein. Gut mucosal cells efficiently absorb both forms of folate, but the transport out of the cell into the portal vein via multidrug resistance protein 3 (assumed to have a key role) is much less efficient for folic acid than for the reduced form. The efficient conversion of folic acid to 5-Methyltetrahydrofolate in mucosal cells has only been shown in animals and cell cultures, but not in humans. On the other hand it has been shown that liver Dihydrofolate reductase activity (catalysing reduction of folic acid to dihydro folic acid and reduction of dihydro folic acid to tetrahydro folic acid) in human liver is low with a high inter subject variability (Bailey and Ayling 2009). Thus, folic acid has to be removed by the liver from the portal vein, which explains a slow and attenuated response of systemic 5-MTHF and chronic exposure to folic acid might induce saturation and appearance of not reduced folic acid in the systemic circulation (Patanwala, King et al. 2014). In this context it is interesting to note that Niesser et al. have observed urinary excretion of p-aminobenzoylglutamate (a folate catabolite) within an hour after the oral intake of folic acid (Niesser, Demmelmair et al. 2013). Although the corresponding experiment with 5-MTHF has not been performed, higher urinary excretion of folic acid might be related to the slightly lower efficiency of folic acid than of 5-MTHF to increase folate status marker.

It is not fully established, whether the appearance of folic acid in the circulation has negative health consequences (Kalmbach, Choumenkovitch et al. 2008), but reduced natural Killer cell activity has been observed in subjects with detected non

metabolized folic acid in plasma (Troen, Mitchell et al. 2006).

An in vitro study in the 1980ies has suggested that the activity of the different branches of the folate cycles (i.e. transfer of methyl groups to homocysteine or synthesis of tymidine or purines) depends on the form of folate incubated with liver tissue slices (Matthews and Baugh 1980). This could mean that availability of S-Adenosylmethionine is higher if 5-MTHF is provided instead of folic acid, because intermediates of the conversion process of folic acid to 5-MTHF can be used as substrates for purine and pyrimidine synthesis. Thus, folic acid might favour synthesis of DNA bases, while 5-MTHF might favour methylation reactions. This has not been shown so far, but if it occurs, it may be of relevance as the pattern of folate species has been found related to global DNA methylation in human mucosa cells (Liu, Hesson et al. 2012).

There are only limited data available on infantile plasma or red blood cell levels of non-metabolized folic acid, but Obeid et al. have detected folic acid in 55 % of the analyzed cord blood samples (Obeid, Kasoha et al. 2010). In adults living in countries with (USA) and without (Ireland) mandatory fortification with folic acid, in a high percentage of the subjects non metabolised folic acid could be detected in plasma (75 % and 94 %, respectively) with levels related to dietary intake (Kalmbach, Choumenkovitch et al. 2008, Boilson, Staines et al. 2012). Although suggested by the US data (Kalmbach, Choumenkovitch et al. 2008) the relevance of the Dihydrofolatereductase genotype for folic acid levels could not be confirmed in the data from Ireland. Nevertheless, in the relatively small group of 137 Irish subjects also no effect of the 5,10 methylen tetrahydrofolate reductase genotype could be identified, although its relevance for the one carbon metabolism has convincingly been demonstrated before. As dietary intake has been identified as relevant, it is interesting to look at the breast milk folic acid content, which could serve as a reference for infantile intake. Houghton et al did not find different effects of supplementation with 5-MTHF, folic acid (906 nmol/d each) or placebo on milk folate levels and identified in average 8% of milk folate as folic acid without differences between supplementation groups (Houghton, Yang et al. 2009). In 28 lactating women intake of about 750 µg of supplemental folic acid from postpartum week 5 to week 15 did not lead to an increase of total milk folate (around 60 µg/l) but to a slight increase of the percentage of folic acid from 32 % to 40 % (West, Yan et al. 2012). Thus, reduced folate species are dominating in breast milk, while in infant formulas folic acid is the major species (Kim, Yang et al. 2004).

The recent observation by Hartmann et al., that supplementation with 1 mg per day of folic acid does not alter the pattern of folate species in red blood cells (Hartman, Fazili et al. 2014), does not necessarily mean that distribution of methyl groups via the folate cycle is not altered by supplementation, it may well be that other cells (e.g. hepatocytes) are more exposed to folic acid or that it is more relevant to measure reaction products (e.g. methionine to homocysteine ratio or DNA methylation) than folate cycle intermediates.

Although the provision of adequate amount of folate with the infantile diet is of primary importance, the available evidence suggests that especially in infants dietary folate species may be of importance for current and long term wellbeing. While breast milk folate species distribution seems to respond hardly to dietary intervention, a comparison of folic acid and 5-MTHF in respect to infantile outcome and biomarkers of folate metabolism seems warranted and feasible.

1.2 Investigational product

Except the new folate source of the intervention formula, the intervention infant formula as well as the control infant formula meet the regulatory standards established by the EU-directive 2006/141/EC on infant formulae and follow-on formulae. Up to now according to Annex III table 1 “Vitamins” of the directive folic acid is the only allowed source (“vitamin formulation”) of folate as used in the control formula.

1. Intervention formula: modified HiPP 1 Bio Combiotic low protein infant formula with Metafolin (MTHF) instead of usual folate source.

2. Control formula: standard HiPP 1 Bio Combiotic low protein infant formula with folic acid as folate source

For details of the composition of the formulae please see description in the “Technical Information”.

1.3 Rationale for conducting the biomedical research project

It appears potentially beneficial to provide L-5MTHF rather than folic acid with infant and follow-on formula to achieve a supply of the physiological form of folate and to avoid high plasma concentrations of free folic acid which have been associated with potential adverse effects, but no data from clinical studies in infants are available. This study aims at characterising safety and efficacy of L-5MTHF compared to folic acid in infant formula on growth and development in healthy infants born at term. As 5-methylTHF is the major transport form of folate, reduced MTHFR activity is expected to decrease the amount of circulating folate, possibly exacerbating its metabolic impact. Individuals who are homozygous for the MTHFR C677T variant (i.e. TT genotype) exhibit lower specific activity of MTHFR and reduced stability of the enzyme (Jacques, Bostom et al. 1996, Hustad, Midttun et al. 2007).
This study is part of a dossier to be sent to the European Commission for admission of adding L-5-Methyltetrahydrofolate to infant and follow on formula.
L-5-MTHF is the predominant form of folate found in human breast milk. The supplementation of MTHF (instead of folic acid) to infants fed with infant formulas may help to increase L-5-MTHF and reduce unmetabolized folic acid plasma levels in this population. Thus, the plasma MTHF levels of bottle fed infants may become more similar to those of the breast fed population.

2 OBJECTIVES

2.1 Primary objective and endpoint variable

2.1.1 Primary objective

Primary objective is to show equivalence of an infant formula containing L-5-Methyltetrahydrofolate (“MTHF”) compared to a standard infant formula containing folic acid on weight gain of healthy term infants receiving exclusively these infant formulae in the first 4 months of life.

Research question: Is a modified infant formula containing MTHF suited to ensure normal growth and development of healthy children during the first 4 months of life (in comparison to a standard infant formula containing folic acid)?

<u>Primary target variable – hypothesis (H_0) to be rejected:</u>
The mean daily weight gain in the intervention group (MTHF group) is **either** at least 3.5 grams per day **lower** than the mean daily weight gain in the control group (standard formula containing folic acid) **OR** at least 3.5 grams per day **higher** than in the control group

Hypothesis H_A: The mean daily weight gain in the intervention group is less than 3.5 grams per day lower than the mean daily weight gain in the control group **AND** less than 3.5 grams per day higher than in the control group.
MTHF infant formula is suitable for the particular nutritional use by infants from birth onward.

2.1.2 Primary endpoint variable

Infant weight gain in gram per day between age of four weeks and 16 weeks (total weight gain between V1 and V4 divided by the number of days between those visits)

2.2 Secondary and exploratory objectives and endpoint variables

2.2.1 Secondary objectives

Secondary objectives are

- to show that parameters of growth and development (body length and fronto-occipital head circumference) are clinically equivalent between the groups and comparable to a reference group of breast fed infants. Clinical equivalence is demonstrated if the 95% confidence interval of the difference between intervention and control group is contained within the equivalence range (-0.5 sd, 0.5 sd).
- to show that energy intake in both infant formula groups is clinically equivalent. Clinical equivalence is demonstrated if the 95% confidence interval of the difference in energy intake between intervention and control group is contained within the equivalence range (-5% of total energy intake in control group, 5% of total energy intake in control group);
- to show that tolerability of both infant formulae is good, as assessed by 3-day diary;

2.2.2 Exploratory objectives

Exploratory objectives are

- to show that folic acid status (plasma levels of 5-MTHF, FA, fTHF, hmTHF, pABG, apABG to assess current folate supply, and red cell total folate [RCF] to assess longer term folate supply) in blood samples of infants receiving the intervention infant formula instead of the control formula is more comparable in intervention than control to that of a reference group of fully breastfed infants;
- to assess the frequency of the homozygous genotypes of common single nucleotide polymorphisms in methylentetrahydrofolate-reductase (MTHFR) mutations C677T and A1289C and their association with the folate status in infants (blood cells will be stored for eventual later epigenetic analyses);
- to assess the potential long-term impact of the intervention vs. control, compared to reference, on growth parameters, haematology, and adverse events at age of 1 year;
- to assess the plasma levels of 5-MTHF, FA, fTHF, hmTHF, pABG, apABG and blood hematology of the breast-feeding mothers;

- to assess breast milk levels of 5-MTHF, FA, fTHF, hmTHF, pABG, apABG;
  to assess maternal intake of folic acid and MTHF.

2.2.3 Secondary endpoint variables (between week 4-16):

- Body length gain (in cm/day) and fronto-occipital head circumference gain (in cm/day)
- Dietary intake of study formula and non-study formula, additional liquids and solids (expressed as kcal per day and as kcal per kg body weight per day) as assessed by 3-day diary
- Feeding related behaviour (acceptance, formula tolerance, spitting, vomiting, crying, sleeping time per day) based on 3-day diary; stool characteristics (color and consistency) assessed by the Bristol scale criteria included in the 3-day diary

2.2.4 Additional parameters

- Plasma levels of 5-MTHF, FA, fTHF, hmTHF, pABG, apABG and red cell total folate levels (RCF) at Baseline Visit and Visit 4
- Frequency of the homozygous C677T and A1289C genotypes of MTHFR in infants at Baseline Visit (blood cells will be stored for eventual later epigenetic analyses)
- Growth parameters (weight, length, head circumference) and haematology (haemoglobin, HCT, MCV) at age of 1 year
- Plasma levels of 5-MTHF, FA, fTHF, hmTHF, pABG, apABG and blood hematology of the breast-feeding mothers at Visit 2
- Breast milk levels of 5-MTHF, FA, fTHF, hmTHF, pABG, apABG at Visit 2
- Maternal intake of folic acid and MTHF based on questionnaire at Visit 2

2.3 Safety objectives and endpoint variables

2.3.1 Safety objective(s)

To assess safety and tolerability of the intervention product and control product.

2.3.2 Safety endpoint variable(s)

- Assessment of health and intermittent clinical conditions at visits 1 to 4
- Standard clinical safety panel, including hematology (haemoglobin, HCT, MCV) and serum biochemistry (creatinine and ALAT)
- AE/SAE reporting according to DNP SOPs
- Follow up visits at the age of 12 months and determination of weight, length, head circumference, haemoglobin, HCT, MCV, and occurrence of adverse events

3 SITE AND SUBJECTS

3.1 Number of subjects

In total, 360 (n=120 in control group, n=120 in intervention group, n=120 in reference group) will be recruited. Using these assumptions and requiring a power of 80% and a type I error rate of 2.5%, sample size was calculated using Julious *(Sample Sizes for Clinical Trials, CRC Press 2010, Formula (5.10), pg. 85)*, for equivalence clinical trials

with normal data in the special case of no treatment difference, resulting in group sample sizes of 84 analyzable infants per group. Since we expect a drop-out proportion of approximately 30 percent – a true loss to follow up of about 10-15% and another 15% which will switch formula due to preference changes – 120 infants will have to be recruited per formula group.

3.2 Number of sites

The study will be performed at one site:

Recruitment is performed at:

KBC "Dr Dragiša Mišović-Dedinje"
Heroja Milana Tepića 1
11000 Belgrade, Serbia

Prim. Dr. Milica Vusurovic (Principal Site Investigator)
Tel. +381 (0) 63 312 171
Email: milica.vusurovic@gmail.com

Enrollment and clinical examinations are performed at:

Hipp Clinical Study Center, Belgrade
Kneza Milosa 51
Belgrade, Serbia

Dr. Branka Triŝic (Local Project Coordinator)
Tel.: +381 (0)11 3612 944
Fax: +381 (0)11 3612 944
E-Mail: hipp@technicom.net

3.3 Biomedical research project population

3.3.1 Inclusion criteria

- Written informed consent (by parents, caregiver)
- Healthy male or female infants <28 days of life
- Gestational age ≥37 and ≤42 weeks
- Birth weight between 2500 – 4500g
- Parents / Caregivers are able to speak Serbian language

3.3.2 Exclusion criteria

- Serious acquired or congenital diseases that is expected to interfere with normal feeding or growth
- Feeding of more than 10% of energy (1 bottle/day) from sources other than the formula (or breast milk in the reference group) at inclusion
- Participation in another clinical study
- Mothers with diabetes mellitus (including GDM)

- Reason to presume that the parents are unable to meet the study plan requirements
- Diseases of the mother which have an effect on the child's gastro-intestinal tract/ability to be fed
- Major abnormalities in hematological parameters
- Major abnormalities in hepatic, renal or metabolic functions
- Use of medication and vitamin supplements except vitamin K or D supplementation or vaccination
- Mother follows a vegan diet

4 INVESTIGATIONAL PRODUCT AND SUPPLEMENTATION REGIMEN

4.1 Investigational product

4.1.1 Investigational product name and formulation

Product category:	Infant formula „1“ , from birth onwards
Study Name of the product:	HiPP Fol-SN
Name of the product:	HiPP 1 Bio Combiotic low protein infant formula
Net content:	500 g = 2 x 250 g
Form:	Powder
Storage conditions:	Closed, dry, at room temperature (25°C)
Characteristics	With new folate source

For details please see description in the “Technical Information”.

4.1.2 Control product name and formulation

Product category:	Infant formula „1“ , from birth onwards
Study Name of the product:	HiPP Fol-SN
Name of the product:	HiPP 1 Bio Combiotic low protein infant formula
Net content:	500 g = 2 x 250 g
Form :	Powder
Storage conditions:	Closed, dry, at room temperature (25°C)
Characteristics:	With usual folate source

For details please see description in the “Technical Information”.

4.1.3 Packaging and labelling

The investigational product is packaged in white 500g-paper-boxes. Each box contains 2 bags. Four boxes are put together in a covering box including measure scopes. Declaration on the 500g-paper boxes is done in German and Serbian language. The study formulae are named “HiPP Fol-SN”. This name is an artificial term for study purposes.

The label of the bags contains the following information:
- HiPP Fol-SN
- Retest date
- Batch number
- Manufacturing date

The label of the formula box contains the following information in German (and Serbian) language:
- Protocol number
- Subject ID
- Batch number covered by Randomization number
- Product name: HiPP Fol-SN von Geburt an/from birth onwards
- Usage: “Ware nur für Studienzwecke”/”Study formula for clinical trial use only”
- Instructions for preparation of formula
- Information where to find the retest date
- Information about dosage and drinking amounts
- Important notice
- Storage conditions
- Contact data of the study team: “Bitte wenden Sie sich bei Fragen an Ihr Studienteam”/“In case of questions, please contact your paediatric investigator”
- Address of the Distributor: HiPP GmbH & Co. Vertrieb KG
D-85265 Pfaffenhofen
- Name and address of the Sponsor
- Net weight: 500g

The covering box contains the following information (in English):
- Protocol number
- Subject ID
- Product name: HiPP Fol-SN Study formula for clinical trial use only
- Name and address of the Distributor
- Name and address of the Principal Site Investigator
- Contact data of the study team
- Name and address of the Sponsor
- Instructions for use: see label of inner formula box
- Storage conditions
- Net weight
- Randomization number
- Retest date

For details and an example of the label please see description in the “Technical Information”.

4.1.4 Handling and storage conditions

The infant formulae are available as powder. The preparation will be carried out as described on label of the package with the provided spoon (for details see “Technical Information”). Parents / Caregivers will be advised to follow these descriptions carefully.
The powder has to be stored at a dry, not too warm place (room temperature) and has to be closed carefully. It has to be used within three weeks after opening the bag.

Quality control is performed by the producer at the stage of raw materials, during production and on product release. The shelf life of the study formulae is 15 months. A retest date is printed on each package.

4.2 Supplementation regimen

4.2.1 Rationale for dose selection

The dosis of MTHF was calculated based on the bioavailability and active part of both substances (folic acid and MTHF) considering water content molecular weight and “acid as is”= activity. The bioavailability is equally for MTHF and folic acid. The difference in activity is 1.3, which was used as a factor to calculate the amount of MTHF.

Infant formulae are fed ad libitum. Parents/caregivers are advised on the typical amount of formula consumed by healthy infants based on energy density. Advice will be provided on appropriate reconstitution of the powdered formula with particular emphasis on provision of the correct concentrations. Following current recommendations, parents/caregivers are advised to provide formula feeding ad libitum (Koletzko, Bauer et al. 2013).

4.2.2 Dosage regimen and dose adjustment

As stated above, infant formula feeding ad libitum is recommended.

4.2.3 Route of administration

In the healthy infants studied here, oral feeding usually from a standard infant feeding bottle will be used. In exceptional situations, e.g. intercurrent illness, feeding by cup and spoon or feeding from a syringe may be used for a short period of time but this is not encouraged for regular feeding.

4.2.4 Supplementation duration

Control and intervention formula are provided from the time of inclusion at any time after birth until the end of intervention at the infantile age of 16 weeks.

4.3 Dispensing and accountability

The producer will provide a Tracking log for the shipment of investigational products from central stock in Germany (location of packing and blinding; SCA, Ingolstadt) to the central stock in Belgrade (Alca) and from stock to study site. The investigator will receive a Product Dispensing and Return Log in order to account for investigational product dispensed to and returned by the site. In addition an individual product dispensing log per subject has to be maintained. It must be kept up-to-date and list the subject ID, the amount of investigational product and date dispensed to the subject and the amount of product and date returned. Subjects must be instructed to return empty 500g-paper-boxes on each visit.

If applicable, the monitor will inspect during visits the amount of investigational product dispensed, returned and on stock at the site (including empty containers, partly used and used product). The investigator must return all unused product to DNP or discard as agreed with the Study Director at the end of the study.

Parents will receive first formula boxes at Baseline Visit if the infant is randomized at this visit. If not the parents will receive formula boxes after randomization. Change

from another formula or from breastfeeding to exclusive study formula-feeding must be carried out until beginning of day 28 (run-in phase). Per definition day 28 is the latest possible day to fully feed the study formula for the first time. Exclusive formula feeding must start at that day and then continue until Visit 4 (16 weeks ± 3 days of life).
In case the healthy infant is not breastfed, control formula as well as modified study formula is suitable to be fed from birth onwards. The exact amounts consumed will be written down in the 3-day diary.

4.4 Compliance

An energy intake of 10% or more (which corresponds to ≥50 ml) of daily energy from liquid or solid foods other than breast milk or study formula will be considered non-compliance, and those subjects will be excluded from per-protocol analysis.

4.5 Protocol Deviations/Violations

A protocol deviation/violation is any noncompliance with the clinical study protocol or Good Clinical Practice. As a result of deviations, corrective actions are to be developed by the study staff and implemented promptly. However, no deviation from the protocol will be implemented without the prior review and approval of DSM and the IRB/IEC except where it may be necessary to eliminate an immediate hazard to a research subject. In such case, the deviation will be reported to DSM and the IRB/IEC as soon as possible.

Concerning scheduled assessments, a time difference of more than ±3 days for V1-V4 and ±7 days for FUV will be considered as protocol deviation/violation. For further conditions see Section 4.7 “Concomitant treatments/supplements and restrictions”.

A **significant** protocol deviation/violation is a deviation/violation that has an impact on subject safety, may substantially alter risks to subjects, may have an effect on the integrity of the study data, or may affect the subject’s willingness to participate in the study. Significant protocol deviations/violations can vary in the degree of seriousness according to how the changes impact subject safety, the degree of non-compliance with local regulations and applicable laws, the study’s IRB/IEC, and the degree of foreknowledge of the event.

An example of **significant** protocol deviation/violation is absence of weight measurement at V1 and/or V4.

All deviations/violations from the protocol must be addressed in study subject source documents and significant deviations/violations must be promptly reported to DSM and the local IRB/IEC, according to their requirements.

4.6 Warnings and precautions

Please pay careful attention to the instructions when preparing infant formula. Incorrect preparation or storing a prepared bottle for a longer period of time can be harmful to health due to undesired bacterial growth.
For this reason, always <u>prepare every meal from scratch</u> and <u>feed immediately</u>. Do not re-use leftovers. Clean bottle, teat and ring thoroughly. Close the opened sachet tightly after every use, store in a cool and dry place and use up within three weeks.
Do not heat milk formula in a microwave oven (danger of scalding).
For details please see description in the “Technical Information”.

4.7 Concomitant treatments/supplements and restrictions

Preparations containing B vitamins provided to babies for a duration of more than three days or for any duration during the week preceding a blood sample collection will be considered a protocol violation.

5 STUDY DESIGN AND SUBJECT ASSIGNMENT

5.1 Study design

Mono-centric, randomized, double-blind, parallel-group, controlled prospective intervention study in healthy infants receiving one of the following preparations:

Group 1: Infant formula including folic acid within the range stipulated by European legislation on infant formula (control group, n=120)
Group 2: Infant formula including MTHF instead of folic acid (intervention group, n=120)
Group 3: Breast milk (reference group, n=120)

5.2 Duration of biomedical research project

5.2.1 Duration of biomedical research project

The first subject in is expected from Q1 2015 onwards.

The study will be performed in two phases:

1. Main study: Database will be locked after 4 month of age of the last infant and availability of all laboratory results, statistical analyses will be performed and main study report will be prepared
2. Follow-up study: While the study centre remains blinded, additional visit at the age of 12 month will be performed and additional report for the follow-up will be prepared

The study duration is expected to be 36 months.

5.2.2 Duration of biomedical research project per subject

Subjects will be fed study formula for a maximum duration of 4 months (birth to age 112 days) and a minimum duration of 3 months (age 28 days to age 112 days). The FUV takes place at the age of 12 month of the subject.

5.3 Randomization, blinding and treatment allocation

Mothers will be advised to exclusively breastfeed their child for at least four months. But they will be informed that, in case they decide to stop exclusively breastfeeding against all recommendations within the first 4 weeks of life – due to any reason not related to this study – they can contact the study team and get study infant formula for free.

Infants will be randomised into one of the study formula groups and receive a Randomization Number from the random list (Random Block Size = 6) stratified

according to gender (male = random numbers 1 – 150 and female = random numbers 150-300), starting at top of the lists (e.g. 1 which corresponds to random number R001 in the eCRF documentation). This random number will not replace the Subject ID Number; but should be reported on all documents and biological samples in addition to the Subject ID Number (e.g. S0001).

All study formula boxes will be blinded on authority of the distributor (HiPP) according to Sponsor´s requirements (study design). Random number list will be obtained from the statistical department of Univ. of Munich Medical Centre and provided directly to the logistic partner responsible for blinding (SCA Full Filement LTD, Bruhnstr. 15, 85053 Ingolstadt).
The products will be packed in identical white boxes and labelled with the same product name. The batch number will be printed on each formula box (covered by the Randomization number) and inner bag. Proper supply of all nutrients will be assured by adding appropriately sized spoons to the blinded packages. The identity of the specific product will be blind to subjects, study staff and investigators. Boxes are marked with specified Study Formula Randomization Number according to Random number list. Unblinding can be done immediately in urgent needs (see 5.4).

5.4 Unblinding procedure

Study personnel will remain blinded to the treatment assigned to subjects throughout the study. The Principal Investigator, the Principal Site Investigator and UBC Pharmacovigilance will receive sealed code envelopes for each subject enrolled into the biomedical research project identifying which treatment the subject receives. The code envelopes are for emergency use only, i.e. when it becomes absolutely necessary to know what treatment the subject receives in case of a serious adverse event (SAE) in order to manage the subject's condition. A broken code requires the subject to be withdrawn from the biomedical research project.

The Principal Site Investigator should promptly document and explain to the Principal Investigator and the Sponsor any premature unblinding (e.g., accidental unblinding, unblinding due to a SAE) of the investigational product. The date, time and reason for opening the code envelope (unblinding) must be written onto the envelope and signed by the Principal Site Investigator and has to be documented in the eCRF.

If appropriate, the monitor will check the intactness of the seal during monitoring visits and adequate documentation of broken seals.

After the biomedical research project, all unbroken and broken envelopes are collected by the Sponsor.

6 STUDY PROCEDURES

6.1 Recruitment procedures

Parents / caregivers will receive information about the study before birth or within the first few days after birth (from birth to the 5th day of life) at the recruitment site from a member of the study team. Care will be taken that no interference with breast feeding occurs. Infants of parents who agree to participate in the study are checked for inclusion and exclusion criteria. In oral agreements with parents, personal details of the parents are then forwarded to the Clinical Study Center Belgrade for contacting

or in case of interest, parents / caregivers will get contact information and are asked to get in touch with the study team, respectively. In compliance with the CONSORT statement the number of subjects assessed for eligibility will be reported.

Prior to the Baseline Visit, parents / caregivers will receive Parent Information and an Informed Consent form from the study team. The informing conversation should be made by educated study staff, the paediatric physician primarily involved in conducting the study or by a colleague paediatric or obstetric physician that is familiar with the study design and is able to answer parents / caregivers questions in detail. During the parent information conversation the investigator should clarify whether the infant will fit to the inclusion and exclusion criteria (as described in the "Parent Information") to prevent parents / caregivers of non-eligible subjects from signing the informed consent. The Principal Site Investigator must clarify if parents / caregiver are able to understand and speak Serbian language fluently to make sure they will understand all aspects relevant for them. Subjects are recruited, if their parents / caregivers gave written informed consent and if they had been successfully screened for inclusion and exclusion criteria.

Subjects recruited for the study will be assigned to Subject ID Number which is documented. The Subject ID Number will accompany parents / caregivers and subject throughout the study duration. All blood and milk samples as well as all diaries and visit documentation sheets will be coded with this Subject ID Number.

Randomization will be done ideally at the Baseline Visit, but can be done until before day 28.

6.2 Schedule of assessments

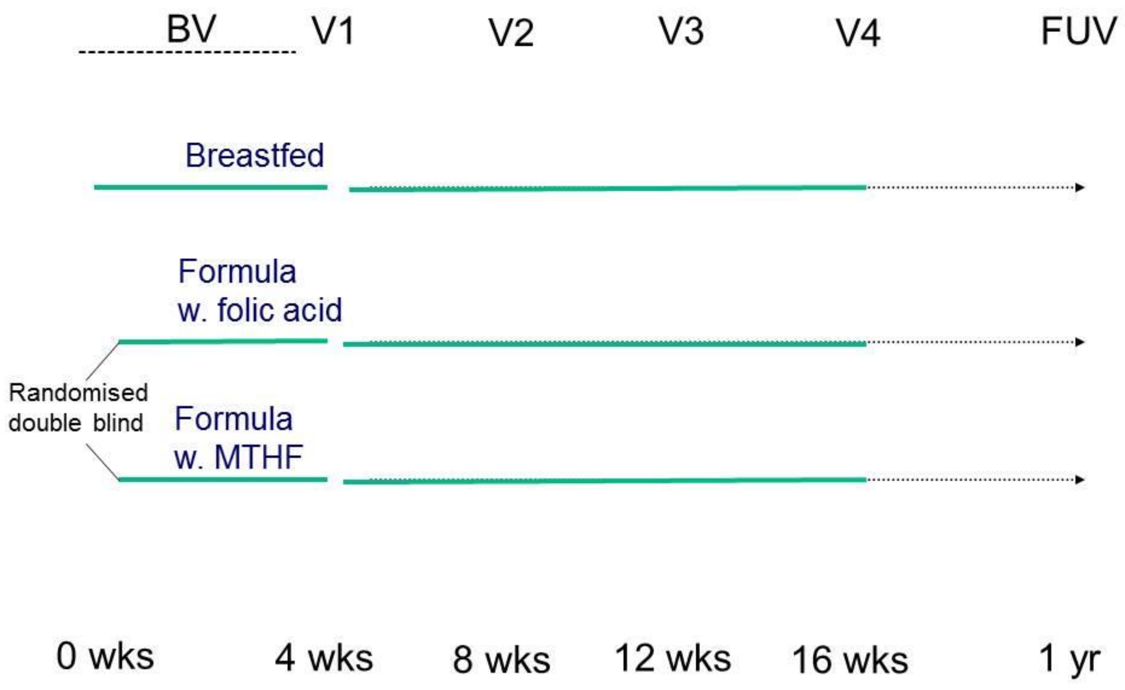

SCHEDULED ASSESSMENTS:

	Assessment periods					
Visits	**Baseline Visit**	**V1**	**V2**	**V3**	**V4**	**Follow-up visit**
Time	Between Day 1 and Day 27 of life	At the age of 4 weeks (±3 days)	At the age of 8 weeks (±3 days)	At the age of 12 weeks (±3 days)	At the age of 16 weeks (±3 days)	At the age of 12 months (±7 days)
**Assessments / Procedures**						
Inclusion/exclusion criteria	X					
Informed Consent	X					
Randomization	X					
Medical examination	X	X	X	X	X	X
Anthropometric data (weight, length, head circumference)	X	X	X	X	X	X
Blood sampling (plasma levels of 5-MTHF, FA, fTHF, hmTHF, pABG, apABG, total RCF, genotype at Baseline Visit)	X				X	
3-day diary (incl. Bristol scale) prior to each visit		X	X	X	X	
Questionnaire for the assessment of maternal supplement intake (folic acid, MTHF)			X			
Maternal blood sampling from the breast-feeding mothers (plasma levels of 5-MTHF, FA, fTHF, hmTHF, pABG, apABG, hematology)			X			
Breast milk sampling (levels of 5-MTHF, FA, fTHF, hmTHF, pABG, apABG)			X			
IP accountability	X	X	X	X	X	
**Safety**						
Adverse event reporting	\---/					
Assessment of health and intermittent clinical conditions		X	X	X	X	
Serum biochemistry (creatinine, ALAT)	X				X	
Hematology (haemoglobin, HCT, MCV)	X				X	X

6.3 Screening and eligibility

6.3.1 Obtaining informed consent

Parents / caregivers of subjects who are foreseen to fulfil the inclusion / exclusion criteria for enrolment into the biomedical research project will be asked to give informed consent in writing prior to any biomedical research project specific procedures. Both the parents / caregivers of the subject and investigator will sign and date the informed consent form. All subjects whose parents / caregivers have signed the informed consent form will be listed on the *Subject Screening and Enrolment Log* and *Subject Identification Log*.

Specific precautions and care will be taken not to actively discourage breastfeeding, and not to suggest bottle feeding to parents of a breastfed baby.

6.3.2 Baseline Visit between Day 1 and Day 27 of life

The baseline visit ensures the eligibility of the volunteers to be included in the trial. During the baseline visit (between Day 1 and Day 27 of life) subjects will be screened as to their eligibility to take part in the study. This process will include:

- *Written informed consent*
- *Review of the inclusion/exclusion criteria*
- *Randomization*
- *Medical examination*
- *Anthropometric measurements (weight, length, head circumference)*
- *Blood Sampling for determination of plasma levels of 5-MTHF, FA, fTHF, hmTHF, pABG, apABG, total RCF, genotype and Serum biochemistry (creatinine, ALAT) and hematology (hemoglobin, HCT, MCV)*
- *Start of AE reporting*
- *In case of Randomization: Start of IP Accountability*

6.3.3 Assessment of eligibility and randomization procedure

Based on screening examinations, the investigator will assess the subject's eligibility to be entered into the biomedical research project. On the *Subject Screening and Enrolment Log*, the decision whether to randomize/enter the subject into the biomedical research project or not is documented.

6.4 Intervention phase

6.4.1 Visit 1 at the age of 4 weeks

The following assessments are done:

- *Medical examination*
- *Anthropometric measurements (weight, length, head circumference)*
- *3-day diary (incl. Bristol scale) prior to each visit for assessment of health and intermittent clinical conditions*
- *IP Accountability*
- *Ongoing AE reporting*

6.4.2 Visit 2 at the age of 8 weeks

The following assessments are done:

- *Medical examination*
- *Anthropometric measurements (weight, length, head circumference)*
- *3-day diary (incl. Bristol scale) prior to each visit for assessment of health and intermittent clinical conditions*
- *IP Accountability*
- *Ongoing AE reporting*
- *Maternal blood sampling from the breast-feeding mothers (plasma levels of 5-MTHF, FA, fTHF, hmTHF, pABG, apABG, hematology*
- *Breast milk sampling (levels of 5-MTHF, FA, fTHF, hmTHF, pABG, apABG*
- *Questionnaire for the assessment of maternal supplement intake (folic acid, MTHF)*

6.4.3 Visit 3 at the age of 12 weeks

The following assessments are done:

- *Medical examination*
- *Anthropometric measurements (weight, length, head circumference)*
- *3-day diary (incl. Bristol scale) prior to each visit for assessment of health and intermittent clinical conditions*
- *IP Accountability*
- *Ongoing AE reporting*

6.4.4 Visit 4 at the age of 16 weeks

The following assessments are done:

- *Medical examination*
- *Anthropometric measurements (weight, length, head circumference)*
- *3-day diary (incl. Bristol scale) prior to each visit for assessment of health and intermittent clinical conditions*
- *IP Accountability*
- *Blood Sampling for determination of plasma levels of 5-MTHF, FA, fTHF, hmTHF, pABG, apABG, total RCF, genotype and Serum biochemistry (creatinine, ALAT) and hematology (hemoglobin, HCT, MCV)*
- *Ongoing AE reporting*

6.5 Follow-up phase

6.5.1 Follow-up visit at the age of 12 months

The following assessments are done:

- *Medical examination*
- *Anthropometric measurements (weight, length, head circumference)*
- *Blood Sampling for determination of hematology (hemoglobin, HCT, MCV)*

7 MEASUREMENTS AND ASSESSMENTS

7.1 Blood and milk samples

During the Baseline Visit, Visit 4 and Visit 5 blood sampling in the infants is planned and associated with Visit 2 blood and breast milk collected from breast feeding mothers. Analyses to be performed are different between subject groups and time points, which is reflected in different SOPs for the respective collections.

SOP infant baseline and V4 (Appendix 7)
SOP mother (Appendix 8)
SOP infant V5 (Appendix 9)

time point (visit)	infant	breast feeding mothers	parameters
Baseline	SOP infant: baseline, V4	no sampling	blood folates, creatinine, ALAT, blood count, DNA
V1	no sampling	no sampling	---
V2	no sampling	SOP mother	blood and milk folates, blood count
V3	no sampling	no sampling	---
V4	SOP infant: baseline, V4	no sampling	blood folates, creatinine, ALAT, blood count, DNA
V5	SOP infant V5	no sampling	blood count

7.1.1 Blood sample collection

Blood sampling in the infants and the breast feeding mothers (V2 only) is performed according to the clinical routines in the study center. Blood drawing shall be done in infants at least 3 hours after the last formula or breast feeding (Baseline Visit and Visit 4), while in the mothers fasted blood samples shall be obtained. In the infants the time since the last feeding (formula, breast milk) and the in case of formula the volume shall be recorded (Baseline Visit and Visit 4). Blood sampling at V5 can be performed independent of prandial state. Blood samples are required for folate related analyses, blood count, clinical chemistry (ALAT, creatinine) and DNA collection, which requires collection of more than one primary tube, i.e. EDTA blood (about 2 ml) and native blood (about 0.6 ml) is required for full blood, plasma and serum based analyses, respectively. As not all measurement are performed from all collected samples different sample handling procedures apply.

7.1.2 Milk sample collection

Breast milk is collected for the determination of the concentrations of 5-MTHF, FA, fTHF, hmTHF, pABG and apABG.
Collection of breast milk is performed by the mothers at home by manual expression of foremilk of the first feed in the morning before breakfast. Four to five ml are divided into three aliquots (about 1.5 ml each), frozen at home and transported refrigerated to the study center. The samples are stored at -80°C until shipment to the laboratories on dry ice together with the blood derived samples (SOP mother).

7.1.3 Blood sample storage and shipment

Blood count (performed from a 200 µl aliquot of the full EDTA blood), ALAT and creatinine (performed from serum derived from the native blood) determination are performed from fresh material according to the routines at the clinical study center at the day of sample collection. For red cell folate analysis an aliquot of full EDTA blood is combined with an ascorbic acid solution to haemolyse cells and preserve folate according to SOPs (Appendix 7, 8). The rest of the sample is centrifuged and separated into plasma and cell sediment (red blood cells and buffy coat) according to SOP. Plasma aliquots and cells are directly frozen at -80° at the study center after labelling. All blood samplings and the details about available aliquots are documented in the

Biological Sampling Log where each sample has to be entered in order to document it's date of withdrawal, place of storage, date of shipment and further details according to SOPs (Appendices 7-9).
Shipment to the analysing laboratories will be performed in one batch after completion of sample collection at V4 from all study subjects. Transport has to be on dry ice using a courier service, preferably World Courier.

7.1.4 Milk sample storage and shipment

Milk aliquots are directly frozen at -80° at the study center after labelling. All milk samplings and the details about available aliquots are documented in the Biological Sampling Log where each sample has to be entered in order to document it's date of collection, place of storage and date of shipment and further details according to SOP mother(Appendix 8).
Shipment to the analysing laboratories will be performed in one batch after completion of sample collection from all study subjects. Transport has to be on dry ice using a courier service, preferably World Courier together with the blood derived samples.

7.1.5 Blood sample assessment

The following assessments are done in the clinical chemistry laboratory at The Institute for laboratory diagnostics Konzilijum, Belgrade from EDTA blood (0.2 ml) and native blood (0.6 ml) respectively.
Using an automatic analyzer Sysmex, model XT-2000i according to the manufacturer`s instructions haematologic analysis including, erythrocytes, leukocytes, thrombocytes, hematocrit, hemoglobin, MCV, MCH, and MCHC, is performed from EDTA blood (infantile samples from Baseline, V4, V5 and maternal samples V2).
From the native blood (0.6ml) serum is obtained by centrifugation according to the SOP (Appendix 7). ALAT is determined by a kinetic UV test on an automatic analyzer (ROCHE, model: COBAS 6000, or 500C) according to the manufacturer`s instructions. Creatinine is determined on the same instruments according to instructions using a kinetic, photometric test (infantile samples Baseline and V4).
From EDTA blood a 0.05 ml aliquot is treated with ascorbic acid solution and stored at -80°C according to SOPs (Appendix 7 and Appendix 8) for microbiological determination of full blood folate using a Lactobacillus casei strain (Molloy and Scott 1997).
Considering the hematocrit and the 5-MTHF concentration in plasma red cell folate is calculated (infantile samples baseline and V4, maternal sample V2).
The remainder of the sample is separated into cells and plasma aliquots by refrigerated centrifugation (Appendix 7), and derived samples are stored at -80°C for later analysis. Two aliquots are used for the determination of 5-MTHF, folic acid, 5-formyl-tetrahydrofolate, 4-Alfa-hydroxy-5-methyl-THF, para-aminobenzoylglutamate, and acetamidobenzoylglutamate using liquid chromatography-tandem mass spectrometry by Bevital, Bergen Norway (Hannisdal, Ueland et al. 2009). While 5-MTHF and folic acid are foreseen as outcome parameters in the study protocol and correspondingly evaluated, the further parameters obtained by the analytical method will only be used for explorative analyses. The other aliquots are stored for eventual later analysis of further metabolites (after irreversible anonymisation of the samples), which may turn out as informative in respect to folate metabolism (e.g homocysteine) (infantile samples baseline and V4, maternal sample V2).

7.1.6 Milk sample assessment

From two of the breast milk sample aliquots determination of the concentrations of 5-MTHF, FA, fTHF, hmTHF, pABG and apABG following the analytical methodology

outlined for plasma is performed by Bevital (Hannisdal, Ueland et al. 2009). The remaining back up aliquot will be stored at LMU München for eventual later analysis of macronutrients or other metabolites which may turn out to be relevant for data interpretation, if not required for folate determinations. Additional analyses will only be performed after anonymization of the samples.

7.2 DNA sample

7.2.1 DNA sample collection

DNA for the study will be obtained from white blood cells, which become available via the collection of EDTA blood (infantile samples baseline and V4). For extraction of DNA, buffy coat and red cells remaining after centrifugation of EDTA blood and removing plasma is kept in the blood sampling tubes and stored at -80°C (SOP infant baseline and V4, Appendix 7).

7.2.2 DNA sample storage, shipment and genotyping

Buffy coat for DNA extraction and red cell remain in the primary collection tube. As all other biosamples, tubes with red cells and buffy coat are stored after labelling at -80°C. After completion of sample collection the tubes are shipped to the genetics laboratory (Helmholtz Zentrum München) on dry ice. The DNA will be extracted from all samples collected from infants (Baseline Visit and V4). Aliquots of the DNA samples collected at baseline will be used for genotyping (analysing SNPs of C677T and A1289C of the MTHFR gene applying Taqman genotyping. Genotyping will include a quality control. In cases where the quality of the DNA collected at baseline is not sufficient, DNA obtained at V4 will be used.
Only for explorative purposes cells (or extracted DNA) are stored (Appendix 7) for eventual later epigenetic analyses after anonymization (infantile samples baseline and V4). It is well accepted that micronutrietens including folate and other B vitamins influence DNA methylation and gene expression (Vanhees, Vonhogen et al. 2014). Thus, it is conceivable that DNA methylation status of study infants might be differently influenced by the intake of 5-MTHF and folic acid. This could eventually induce programming of health risks via epigenetics. Unless more advanced techniques become available until the analyses will be done, DNA methylation status will be determined using Infinium Human Methylation Bead Chips (Illumina, San Diego, CA). Genomic DNA will be bisulfite converted and an aliquot of converted DNA will be subjected to the Illumina Infinium methylation assay and hybridized on the bead chips.

7.3 Other assessments

7.3.1 Anthropometric measurements

Anthropometric measurement must be performed within all study visits - as described below (SOP for anthropometric measurements). Always the same investigator or member of the study team has to perform this measurement. To assure that variances through different methods of determination are minimized, study team members in the clinic will be trained together at the beginning of the study.

- Body weight (g): measurement will be done with a calibrated scale with a precision of 1 g. The infant, without diaper, is placed on the scales so that the weight is equally distributed on each side of the centre of the pan. Weight is recorded, to

the nearest 1 g, with the infant lying quietly, which may require patience. The measurement is repeated and recorded twice after excluding any clearly erroneous results.

- Body length / distance crown - sole (cm): Two observers are required to measure recumbent length. The crown of the head touches the stationary, vertical head-board, and the centre line of the body coincides with the centre line of the measuring table. The infant's head is held with the Frankfurt Plane aligned perpendicular to the plane of the measuring table. The shoulders and buttocks are flat against the tabletop, with the shoulders and hips aligned at right angles to the long axis of the body. The legs are extended at the hips and knees and lie flat against the tabletop, with the arms resting against the sides of the trunk. The measurer positioning the head stands behind the end of the table to ensure that the subject does not change position and to check the alignment of the body with the long axis of the table. The second measurer makes sure both head and feet touch the headboard while the legs are straightened without forcing them. He places one hand on the knees to ensure that the legs remain flat on the table. He or she applies firm pressure with the other hand to shift the movable board against the heels. The measurement is repeated and recorded twice after excluding any clearly erroneous value. The length is recorded to the nearest 0.1 cm.

- Head circumference (cm): An infant is measured when recumbent or seated. For this measurement an insertion tape is used. The measurer stands facing the left side of the infant and places the tape so that the zero-end is on the lateral aspect of the head. This involves passing the tape around the head and then transferring the ends of the tape from one hand to the other so that the zero mark on the tape is inferior to the value to be recorded. The tape is positioned so that large amounts of cranial hair (braids) are excluded. Anterior, the tape is placed just superior to the eyebrows and posterior it is placed so that the maximum circumference is measured. The plane of the tape must be the same on both sides of the head. The tape is pulled tightly to compress hair and obtain a measure that "approximates" cranial circumference. The measurement is recorded to the nearest 0.1 cm. The measurement is repeated and recorded twice after excluding any clearly erroneous value.

7.3.2 Questionnaires

The 3-day diaries for both formula- and breastfed groups are to found in Appendix 6.

8 Early Subject Withdrawal

Parents / caregivers of subjects have the right to withdraw subjects from the study at any time for any reason, without being obliged to give reasons and without penalty or loss of benefits they are entitled to. The investigator also has the right to withdraw subjects from the study if this is in the best interest of the subject.

Any of the following conditions leads to premature withdrawal:

- request by parents / caregivers of subjects to discontinue for any reason during the study (withdrawal of consent)
- erroneously included / randomized subject
- adverse event or concurrent illness that, in the opinion of the investigator, warrants the subject’s withdrawal from intervention

- intake of concomitant medication/dietary supplements prohibited by the protocol
- parents / caregivers of subjects who do not follow the requirements of the investigator, especially those concerning safety and/or if the parents / caregivers of subjects after the enrolment are uncooperative or not willing to comply with the protocol (non-compliant)
- failure to return (lost to follow-up)
- request by regulatory agencies for termination of supplementation of an individual subject or all subjects under this protocol

The circumstances of any discontinuation have to be documented in detail in the corresponding study termination form in the case report form. Whenever a subject will be withdrawn from the study for whatever reason, adequate efforts will be made to perform a final assessment to include standard medical and laboratory tests which would have been done on normal completion of the study. The data of the withdrawn subjects will be documented in the clinical study report.

Subjects who withdraw prematurely from the study will not be replaced.

9 STATISTICAL CONSIDERATIONS

Necessary sample size:

- According to the report of the EC Scientific Committee on Food 2003, "growth studies should be designed to have a power to detect a difference in weight gain equal to 0.5 standard deviations". In analogy to this recommendation, we consider the average daily weight gain of infants in the intervention group equivalent to the average daily weight gain of infants in the control group, if it is within the boundaries of +/- 0.5 standard deviations.
- Based on previous observation in Serbian infants (Fleddermann, Demmelmair et al. 2013), we estimate that the infants gain about 30g weight per day with a standard deviation of about 7g.
- We do not expect a difference in average daily weight gain between the group receiving intervention formula and the group receiving standard formula. The standard deviation of the group receiving infant formula with L-5-MTHF is supposed to be the same as in the control group, and comparable to the growth observed in the reference group.
- Using these assumptions and requiring a power of 80% and a type I error rate of 2.5%, sample size was calculated using Julious *(Sample Sizes for Clinical Trials, CRC Press 2010, Formula (5.10), pg. 85)*, for equivalence clinical trials with normal data in the special case of no treatment difference, resulting in group sample sizes of 84 analyzable infants per group. Since we expect a drop-out proportion of approximately 30 percent – a true loss to follow up of about 10-15% and another 15 % which will switch formula due to preference changes – 120 infants will have to be recruited per formula group.
- The group of breast fed infants serves as a reference only. No direct statistical comparison with the control or intervention group is planned.

Analysis populations:

- The primary endpoint will be analyzed using the modified Intent To Treat (ITT) population and the Per Protocol (PP) population. The ITT population is defined as all subjects who completed V1 after initiation of treatment. It is planned that 120 subjects in the formula groups, respectively, and 120 in the reference

group should complete V1. Therefore, dropouts between Baseline Visit and V1 should be replaced (screening failure). The PP population is defined as all subjects that completed the 16 (V4) weeks study without any major protocol violation.

Statistical analysis of primary outcome body weight gain:

- The primary outcome is the daily weight gain in grams between day 28 and day 112 after birth. The primary outcome variable “weight gain per day” will be calculated by subtracting weight obtained at the Visit 1 (28 days of age) from the weight obtained at Visit 4 (at 112 days of age) divided by the number of days between those visits.
- The primary objective is to show equivalence of the two arms, intervention and control. For this, a TOST (two one-sided test) procedure will be performed, taking the cut-off level of statistical significance at 0.025. Because of the duality of test procedure and corresponding confidence interval equivalence of interventional nutrition regime can be proven by means of the interval boundaries. If the 95%-confidence interval for the difference of the mean daily weight gain of intervention and control arm is within the interval (-3.5 grams, 3.5 grams), H_0 will be rejected and equivalence will be concluded.
- The confidence interval for the difference of the mean daily weight gain will be calculated using a linear model on body weight with treatment, time in days and the time-treatment interaction as fixed effects, birth weight and sex as adjusting covariates and subject as random effect.

Further Analyses

- Data will be displayed in absolute values and as mean difference to Visit 1 with CI and SD by treatment group.
- In order to evaluate relative efficacy of the study formulas and breast milk, significant difference between groups for folate status and weight gain will be evaluated. Equivalence testing will be done for major parameters where an adequate equivalence margin can be appropriately defined for the preparation. The relevant margins will be defined before unblinding and initiation of data analysis. The same adjusting covariates as in the primary analysis will be used.

10 SAFETY

10.1 Definitions and Standards

Adverse Event (AE):

Any untoward medical occurrence in a subject involved in a biomedical research project administered an investigational product whether or not related to this product.

An AE can therefore be any unfavourable and unintended sign (including an abnormal laboratory finding for example), subjective and objective symptom, or disease temporally associated with the use of a product, accidents, whether or not considered related to the product or study-related procedure and reported by the subject or observed by the investigator.

Adverse Reaction (AR):

All noxious and unintended responses to a product or clinical trial material related to any dose should be considered as adverse reactions. Adverse reactions are a subset of

all suspected AEs for which there is reason to conclude that the product caused the event.

Suspected Adverse Reaction (SAR)
Suspected adverse reaction means any adverse event for which there is a reasonable possibility that the product or clinical trial material caused the adverse event. A suspected adverse reaction implies a lesser degree of certainty about causality than an adverse reaction, which means any adverse event caused by a product or by clinical trial material.

Serious Adverse Event (SAE):
Any AE that at any dose fulfils at least one of the following criteria:

- Is fatal (results in death)
  (*note*: death is an outcome, not an event)
- is life-threatening
  (*note*: the term "life-threatening" refers to an event in which the subject was at risk of death at the time of the event; it does not refer to an event which could hypothetically have caused death had it been more severe)
- requires inpatient hospitalization or prolongation of existing hospitalization
  (*note*: "inpatient hospitalization" refers to an unplanned, overnight hospitalization)
- results in persistent or significant disability/incapacity
  (*note:* the term "disability/incapacity " means substantial disruption of one's ability to conduct normal life function)
- is a congenital anomaly/birth defect
  (*note:* congenital anomaly/birth defect in offspring of subject taking the product regardless of time to diagnosis)
- is medically significant
- (*note*: Medical and scientific judgement should be exercised in deciding whether expedited reporting is appropriate in other situations, such as important medical events that may not be immediately life-threatening or result in death or hospitalisation but may jeopardise the subject or may require intervention to prevent one of the other outcomes listed in the definition above).

Suspected Unexpected Serious Adverse Reaction (SUSAR):
All suspected adverse reactions that are both unexpected and serious.

Unexpected Adverse Reaction:
An adverse reaction, the nature, or severity or frequency of which is not consistent with the applicable product information (e.g. Investigator's Brochure).

10.2 Adverse event assessment

Expectedness:
An unexpected AE is an event of which the nature or severity is not consistent with the applicable product information.

Causality Assessment:

The causality assessment of an AE to the investigational product will be rated as follows:

- **No (Not related):** The temporal relationship of the clinical event to investigational product administration makes a causal relationship unlikely, or other drugs, therapeutic interventions or underlying conditions provide a sufficient explanation for the observed event.
- **Yes (Related):** The temporal relationship of the clinical event to investigational product administration makes a causal relationship possible, and other drugs, therapeutic interventions or underlying conditions do not provide a sufficient explanation for the observed event.
- **Suspected** There is a reasonable possibility that the investigational product caused the observed event. Suspected implies a lesser degree of certainty about the causality of the observed event

Severity / Intensity:
The severity / intensity of AEs will be graded on a five-point-scale:

- Mild or Grade 1: discomfort noted, but no disruption to normal daily activities.
- Moderate or Grade 2: discomfort sufficient to reduce or affect normal daily activities.
- Severe or Grade 3: inability to work or perform normal daily activities.
- Life threatening or Grade 4
- Death or Grade 5

Outcome of event:
The outcome of an event will be classified as follows:

- Recovered
- Recovered with sequelae
- Ongoing
- Fatal
- Unknown / Lost to follow-up

10.3 Reporting procedures

All Adverse Events (AEs) occurring during biomedical research projects involving human subjects are recorded in the electronic Case Report Form (eCRF) utilizing an AE Form (Appendix1). A list of AEs has to be sent to UBC Pharmacovigilance as well (third-party vendor contracted by DSM) at the end of the study.

Serious adverse events (SAEs) are reported and processed according to the applicable laws and regulatory requirements governing the conduct of biomedical research projects involving human subjects.

Suspected unexpected serious adverse reactions (SUSARs) are reported and processed according to the applicable laws and regulatory requirements governing the conduct of biomedical research projects involving human subjects.

SAE and SUSARs are handled by a third-party vendor appointed by DSM: UBC Pharmacovigilance (UBC).

SAEs and SUSARs must be recorded on the SAE Form (Appendix 2). Following case processing, a blinded International CIOMS-1 Form or a blinded US FDA MedWatch Form 3500A may be generated from the safety database for further expedited reporting.

The directions for completing the SAE Form are detailed in the “Guidelines for completing the SAE Form” (Appendix 3).

For SUSARs, study treatment may be unblinded prior to reporting the case to the applicable regulatory authorities and IRB/IEC.

The handling, processing and reporting of SAEs and SUSARs are described in the study protocol and graphically presented in a flow-chart, attached to the study protocol (Appendix 4).

The Principal Site Investigator or designated study site personnel must notify all SAEs and SUSARs to UBC (see contact details below) by fax or e-mail within 24 hours of first awareness using the study specific SAE form.

CONTACT DETAILS for UBC Pharmacovigilance:

United Biosource Corporation (UBC)
16, Chemin des Coquelicots
1214 Vernier/Geneva
Switzerland
Attention: Mallorie Clement/ Francine Galibert

Fax number : +41 22 596 44 46 or +800 24 25 26 27 (from EU only)
e-mail: EUsafety@ubc.com

- If a subject is hospitalized or hospitalization is prolonged due to the SAE, a copy of the hospital discharge summary will be obtained, if possible.
- If a death occurs and an autopsy is performed, a copy of the autopsy report will be obtained, if possible.

During or at the end of each study, an SAE reconciliation between data entered in the study database and the data entered in the pharmacovigilance database has to be performed if any SAE has occurred in the study. All discrepancies found during the reconciliation are documented in an SAE reconciliation report and corresponding queries are sent to the investigators for clarification.

10.3.1 Responsibilities

- The Principal Site Investigator (PSI) ensures that SAE reporting procedures outlined in the study protocol are adhered to and that all required documentation is up-to-date and that regulatory and IRB/IEC SAE reporting procedures are followed.
- All study personnel at the study site (Study Coordinator, Study Nurse, PSI or designee) who are in contact with clinical trial subjects are responsible for collecting AE information from the subject at each scheduled site visit or during telephone calls with the subject. Therefore, DSM clinical research personnel or

a delegate (e.g. CRO) who initiate and monitor the study are responsible for explaining the procedures for reporting and evaluating AEs to the PSI and all study personnel who come in contact with the subjects/patients.

- During the course of the study, complete reports of all AEs should be entered in the subject's/patient's site source documents, and on the appropriate study case report forms (CRFs).
- A physician is responsible for: identifying and evaluating the severity (mild, moderate or severe) and clinical importance of the AE, taking appropriate medical action(s), and for notifying UBC immediately of an SAE as specified in the protocol (see above) and also for notifying the IRB/IEC. A copy of the source documents and related records should be supplied with the SAE report to UBC.
- Likewise, a physician indicates the causality (relationship) of the AE to the study product.
- For any laboratory abnormality, the PSI will make a judgement as to its clinical significance. If the laboratory value is thought to be clinically significant, the Sponsor or medical monitor may be consulted, and an assessment will be made by the PSI as to its relationship to clinical trial material administration and it will be documented on the AE page of the CRF.
- In case of emergency unblinding in order to manage the subject's medical condition, please refer to section "Unblinding procedure".
- All AEs will be followed until they have abated, or until a stable situation has been reached. Depending on the event, follow up may require additional tests or medical procedures as indicated, and/or referral to the general physician or a medical specialist.
- The PI should comply with applicable regulatory requirement(s) related to the reporting of SAEs to the regulatory authority(ies) and the IRB /IEC.
- At completion of the study, the final lists of AEs and SAEs may be MedDRA coded by UBC.
- The Monitor(s) should review all completed CRF data and should compare CRF entries with information recorded in the source documents. Any discrepancies or omissions in either data source should be discussed with the site personnel who should make the appropriate corrections to the documents. Any recorded SAE or SUSAR which has not been reported to UBC should be discussed with the PSI and should be reported immediately to UBC as specified in the protocol (see above) and also should be reported by the PI to the IRB/IEC.

11 ETHICAL CONSIDERATIONS

11.1 Local Regulations and Declaration of Helsinki

The investigators will ensure that this biomedical research project is conducted in conformance with the principles of the "Declaration of Helsinki" and/or with the laws and regulations of the country in which the research is conducted, whichever affords the greater protection to the individual.
According to the ICH E9 Guideline on Statistical Principles for Clinical Trials, an Equivalence Trial is: "A trial with the primary objective of showing that the response to two or more treatments differs by an amount which is clinically unimportant. This is usually demonstrated by showing that the true treatment difference is likely to lie between a lower and an upper equivalence margin of clinically acceptable

differences.” Therefore this study is considered as an equivalence trial. The above document refers to bioequivalence trials as having pharmacodynamic or pharmacokinetic endpoints, therefore this study is not considered as a bioequivalence trial.

11.2 DNP’s GCP Management Directive

The investigators will ensure that this biomedical research project is conducted in conformance with the principles of GCP, as outlined below:

1. Biomedical research projects involving human subjects should be conducted in accordance with the ethical principles that have their origin in the Declaration of Helsinki, and that are consistent with the principles of GCP (where applicable) and the applicable laws and regulatory requirements governing the conduct of human studies;
2. Foreseeable risks and inconveniences should be weighed against the anticipated benefit for the individual trial subject and the society;
3. The rights, safety, and well-being of the subjects involved in biomedical research are the most important considerations and should prevail over interests of science and society;
4. The available nonclinical and clinical information on an investigational product should be adequate to support the proposed clinical trial;
5. Biomedical research projects involving human subjects should be scientifically sound, and described in a clear, detailed protocol;
6. Biomedical research projects involving human subjects should be conducted in compliance with the protocol that has received prior institutional review board (IRB)/independent ethics committee (IEC) approval/favourable opinion;
7. The medical care given to, and medical decisions made on behalf of, subjects involved in biomedical research should always be the responsibility of a qualified physician;
8. Each individual involved in conducting biomedical research projects involving human subjects should be qualified by education, training, and experience to perform his or her respective task(s);
9. Freely given written informed consent should be obtained from every subject prior to participation in a biomedical research project;
10. All information from biomedical research projects involving human subjects should be recorded, handled, and stored in a way that allows its accurate reporting, interpretation and verification;
11. The confidentiality of records that could identify subjects should be protected, respecting the privacy and confidentiality rules in accordance with the applicable laws and regulatory requirements;
12. Investigational products should be manufactured, handled, and stored in accordance with DNP quality standards, or if applicable, good manufacturing practice (GMP); investigational product(s) should be used in accordance with the approved protocol;
13. Systems with processes and procedures (such as SOPs) that assure the quality of every aspect of biomedical research projects involving human subjects should be implemented.

11.3 Informed Consent

It is the responsibility of the Principal Site Investigator, or a person designated by the Principal Site Investigator (if acceptable by local regulations), to obtain written informed consent from the parents/legal guardians of each subject participating in this biomedical research project, after adequate explanation - orally and in writing (Parent Information sheet) - of the aims, methods, anticipated benefits, and potential hazards of the study and prior to any study procedures. No study procedures may be performed prior to obtaining the informed consent.

In particular, the participant´s parents/legal guardians will be informed about the following:

- how personal and health-related data will be collected and used during the study
- that their identity and personal medical information will not be disclosed.

The investigator or designee must also explain that the participant´s parents/legal guardians are completely free to refuse to enter the subject in the study or to withdraw the subject from it at any time, for any reason without any resulting disadvantage.

All participants will receive a copy of the Parent Information sheet and a copy of the Informed Consent form signed and dated by the Investigator and the participant´s parents/legal guardians (Appendix 5).

11.4 Ethical Committee approval

This protocol and any accompanying material provided to the subject (such as subject information sheets or descriptions of the study used to obtain informed consent) as well as any advertising or compensation given to the subjects, will be submitted by the Principal Site Investigator to the competent Ethical Committee in Belgrade, Serbia. Approval from the competent Ethical Committee will be obtained before starting the study, and should be documented in a letter to the investigator specifying the date on which the Ethical Committee met and granted the approval.

Any modifications made to the protocol after receipt of the Ethical Committee approval must also be submitted by the Principal Site Investigator to the Ethical Committee in accordance with local procedures and regulatory requirements.

11.4.1 Risk-benefit assessment

The risks involved in participating in this study are minimal. The use of L-5-MTHF in this infant growth and tolerance study is not of concern from a safety point of view. Advice will be provided on appropriate preparation of the infant formula with particular emphasis on provision of the correct concentrations. The risk of the blood collection is merely that of a small local hematoma, or an infection.
It appears potentially beneficial to provide L-5MTHF rather than folic acid with infant formula to achieve a supply of the physiological form of folate and to avoid high plasma concentrations of free folic acid which have been associated with potential adverse effects. Thus, the plasma MTHF levels of bottle fed infants are expected to become more similar to those of the breast fed population.

11.5 Confidentiality

11.5.1 Data

All information regarding the nature of the proposed investigation provided to the Investigator (with the exception of information required by law or regulations to be disclosed to the IRB/IEC, the subject, or the appropriate regulatory authority) must be kept in confidence by the Investigator in accordance with all applicable laws and regulations as specified in the Clinical Study Agreement.

11.5.2 Subject Anonymity

The anonymity of participating subjects must be maintained. Subjects will be identified by an assigned subject number on CRFs and other documents submitted to the Sponsor or its designee. Documents that will not be submitted to the Sponsor or its designee and that identify the subject (e.g., the signed informed consent document) must be maintained in strict confidence by the Investigator, except to the extent necessary to allow auditing by the appropriate regulatory authority, the Sponsor or its designee and in accordance with applicable regulatory requirements.

11.6 Subjects' compensation / remuneration

Travel expenses for participation in study visits may be reimbursed to participating families, but no payment is offered for study participation. After the intervention phase, vouchers will be offered for parents of all study groups which can be exchanged for e.g. food supplements or hygienic products.
Any modifications made to the protocol after receipt of the Ethical Committee approval must also be submitted.

11.7 Registration of study in a public clinical trial database

The study will be registered in http://clinicaltrials.gov/ prior to inclusion of the first subject.

12 STUDY DOCUMENTATION AND RECORD KEEPING

12.1 Protocol amendments

Any modification to the agreed protocol must be approved in writing by the Principal Investigator, the Principal Site Investigator, Hipp and DNP. Amendments will be submitted to the Ethical Committee as required. These procedures must be fulfilled before any modification is put into effect. The Protocol Amendments Tracking Form Log shall be updated for each amendment.

12.2 Investigator site file

The Principal Site Investigator must maintain adequate and accurate records to enable the conduct of the study to be fully documented and the study data to be subsequently verified. These documents should be classified into two different separate categories (1) Investigator's Site File (ISF), and (2) source documents.

1. The ISF will contain the protocol/amendments, CRFs and Query Forms, IRB/IEC and governmental approval with correspondence, sample informed consent,

drug records, staff curriculum vitae and authorization forms and other appropriate documents/correspondence etc.

2. Source documents (usually defined by the project in advance to record key efficacy/safety parameters independent of the CRFs) would include subjects' records, physician's, nurse's and research assistant's notes, appointment book, original laboratory reports, ECG, EEG, X-ray, pathology and special assessment reports, signed informed consent forms, consultant letters, and subject screening and enrolment logs.

The Principal Site Investigator, should retain the study documents of the ISF for the retention time specified in the contract with DNP.

Should the Principal Site Investigator, wish to assign the study records to another party or move them to another location, DNP must be notified in advance.

If the Principal Site Investigator, cannot guarantee this archiving requirement at the investigational site for any or all of the documents, special arrangements must be made between the investigator and DNP to store these in a sealed container(s) outside of the site so that they can be returned sealed to the investigator in case of a regulatory audit. Where source documents are required for the continued care of the subject, appropriate copies should be made for storing outside of the site.

12.3 Case Report Form

12.3.1 Electronic Data Capture

<u>Data entry</u>
The Coordination Centre of Clinical Trials (KKS Charité) uses secuTrial® - a Remote Data Entry (RDE) software solution of interActive Systems (iAS) - to capture trial data and transfer them to a central database. SecuTrial® is in compliance with the regulatory standards (FDA CRF 21 Part 11 and GCP) and contains the required features like Audit Trail, roles and rights management concept and electronic signature.
The system provides the electronic case report forms (eCRF) which allows the documentation of trial data online at any time by using a standard internet browser (e.g. Internet Explorer, Firefox, Opera, etc.). Furthermore the eCRF contains functions to perform plausibility, consistency and range checks of the entered data to get a high level of data quality.
The access to the eCRF requires the authentication by the trial participants. All access rights (read or enter data) will be defined depending on their function in the trial (Principle or clinical investigator, CRA, etc.).
The communication between the client-PC and the trial database on the server is based on the secure data-transfer-protocol (SSL - Secure Sockets Layer). This will prevent any illegal access to the trial data by unauthorized parties.

<u>Query Management</u>
There is a Query management tool as an integrated part of the RDE system. This tool allows the communication between the CRA and the clinical investigator at the site. In case of errors or improper data the CRA can set queries to clarify the affected question. The responsible trial participant at the site should correct the values and answer the queries set by the CRA.

Pseudonymization
Due to data safety reasons and to comply with the data privacy protection, the personal data of every patient will be pseudonymized. This ensures the strictly split between the personal data and patient-related dataset (trial data).
The RDE system generates automatically a pseudonym for every new patient. The pseudonym will be a combination of six alphanumeric characters. All trial data of the patient will be linked with this pseudonym. Personal data of the patient will not be saved in the trial database at any time.

12.4 Data management

At the end of the trial or in case of an interim analysis the data will be exported from the trial database. The trial data will be prepared for the statistical analysis.
This DM process contains the plausibility, consistency and range checks of the data. In case of faulty or missing data the data manager will generate DM queries and send them to the site. The data correction will be performed with scripts in SAS or directly in the trial database.
After all data management steps are finished, the cleaned data will be ready for the statistical analysis.

12.5 Source document and source data verification

According to the standards of the data protection law, all data obtained in the course of a human biomedical research project must be treated with discretion in order to guarantee the rights of the subject's privacy. The investigator should agree to allow the monitor/auditor/inspector to have access to any or all the biomedical research project materials needed for source data verification and proper review of the biomedical research project progress.

12.6 Insurance

DNP’s liabilities in connection with the biomedical research project will be covered by an insurance policy in the event of a subject suffering any significant injury, which is proven as being as a direct result of study participation (as specified in the contract). A copy of the insurance contract will be shared with the competent Ethical Committee.

12.7 Monitoring

It is understood that the responsible monitor will contact and visit the investigator regularly and will be allowed, on request, to review the various records of the trial (eCRFs and other pertinent data) provided that subject confidentiality is maintained in accordance with local requirements and as specified in the contract.

The Local Project Coordinator or Study Nurse at the site of the investigation will be instructed by the monitor to ensure that the following responsibilities and tasks are carried out:

- Monitoring the study supplies and, if requested, returning all undispensed supplies to DNP at completion of the study.
- Maintaining all records of the study.
- Checking source documents for legibility and completion at the time they are received from the investigator. After review of study data with the investigator,

the Local Project Coordinator or Study Nurse will complete the formal source documents / eCRFs.

- If required by the study, removing tear-off labels on receipt of double-blind labelled study material prior to its being dispensed to the subject/patient.
- Contacting patients to remind them of their scheduled visits and obtaining a final disposition for every subject/patient who is entered in the study.
- Checking for reasonableness and completeness of source documents and of recorded CRF data before CRFs are reviewed by a CRO (Sermon) monitor at each routine monitoring visit.

It will be the monitor's responsibility to review the study documents (e.g. eCRFs) at regular intervals throughout the study, to verify the adherence to the protocol and the completeness, consistency and accuracy of the data being entered on them. The monitor must have access to laboratory test reports and other subject records needed to verify the entries on the eCRF.

The investigator (or his/her deputy) facilitates the monitoring tasks including the source document verification and agrees to cooperate with the monitor to ensure that any issues detected in the course of these monitoring visits are resolved.

12.8 Quality assurance and quality control

All material used in clinical studies are subjected to quality control. Quality assurance audits may be performed by DNP (or any health authority) during the course of the biomedical research project or after its completion (see Archiving).

The investigator agrees to comply with DNP and regulatory requirements in terms of auditing of the biomedical research project. This includes access to the source documents for source data verification.

12.9 Final Study Report

When all completed eCRFs have been collected and data have been analysed, the results of this biomedical research project are to be documented in a comprehensive study report prepared by the Principal Investigator.

12.10 Archiving

The study documents will be retained by the Hipp Clinical Study Centre Belgrade for at least 15 years after the completion of a biomedical research project involving human subjects. A biomedical research project involving human subjects is completed with the acceptance of the Final Study Report.

The Trial master file (TMF) shall be retained for a longer period, where so required by other applicable legal or regulatory requirements and/or as specified in the contract between DNP and the investigator.

The ISF will be retained according to Section 12.2

12.11 Publication

The results of this biomedical research project shall be published and/or presented at scientific meetings under the leadership of the Principal Investigator. The Principal Investigator agrees to submit all manuscripts or abstracts to DNP and to Hipp prior to submission and to give them four weeks to review and submit comments. This should allow all partners to protect proprietary information and to provide comments based on information from other studies that may not yet be available to the investigator (as specified in the contract).

In accord with standard editorial and ethical practice, DNP and Hipp will generally support publication in their entirety. Authorship will be determined by mutual agreement. If consensus is not achieved, the Principal Investigator takes final responsibility for the publication and authorship.

13 CONDITIONS FOR TERMINATING THE STUDY

The Sponsor, the Principal Investigator and the Principal Site Investigator each reserve the right to terminate the biomedical research project at any time. Should this be necessary, all parties will arrange the procedures on an individual project basis after review and consultation. In terminating the project, DNP and the investigators will assure that adequate consideration is given to the protection of the subject's interests (as specified in the contract).

14 REFERENCES

Bailey, S. W. and J. E. Ayling (2009). "The extremely slow and variable activity of dihydrofolate reductase in human liver and its implications for high folic acid intake." Proc Natl Acad Sci U S A **106**(36): 15424-15429.

Blom, H. J. and Y. Smulders (2011). "Overview of homocysteine and folate metabolism. With special references to cardiovascular disease and neural tube defects." J Inherit Metab Dis **34**(1): 75-81.

Boilson, A., A. Staines, C. C. Kelleher, L. Daly, I. Shirley, A. Shrivastava, S. W. Bailey, P. B. Alverson, J. E. Ayling, A. P. McDermott, A. MacCooey, J. M. Scott and M. R. Sweeney (2012). "Unmetabolized folic acid prevalence is widespread in the older Irish population despite the lack of a mandatory fortification program." Am J Clin Nutr **96**(3): 613-621.

Dominguez-Salas, P., S. E. Cox, A. M. Prentice, B. J. Hennig and S. E. Moore (2012). "Maternal nutritional status, C(1) metabolism and offspring DNA methylation: a review of current evidence in human subjects." Proc Nutr Soc **71**(1): 154-165.

European-Commission (2006). "COMMISSION DIRECTIVE 2006/141/EC of 22 December 2006 on infant formulae and follow-on formulae and amending Directive 1999/21/EC." Official Journal of the European Union(L 401/1).

Fekete, K., C. Berti, I. Cetin, M. Hermoso, B. V. Koletzko and T. Decsi (2010). "Perinatal folate supply: relevance in health outcome parameters." Matern Child Nutr **6 Suppl 2**: 23-38.

Fleddermann, M., H. Demmelmair, V. Grote, T. Nikolic, B. Trisic and B. Koletzko (2013). "Infant formula composition affects energetic efficiency for growth: The BeMIM study, a randomized controlled trial." Clin Nutr.

Hannisdal, R., P. M. Ueland and A. Svardal (2009). "Liquid chromatography-tandem mass spectrometry analysis of folate and folate catabolites in human serum." Clin Chem **55**(6): 1147-1154.

Hartman, B. A., Z. Fazili, C. M. Pfeiffer and D. L. O'Connor (2014). "Neither Folic Acid Supplementation nor Pregnancy Affects the Distribution of Folate Forms in the Red Blood Cells of Women." J Nutr.

Hay, G., C. Johnston, A. Whitelaw, K. Trygg and H. Refsum (2008). "Folate and cobalamin status in relation to breastfeeding and weaning in healthy infants." Am J Clin Nutr **88**(1): 105-114.

Houghton, L. A., K. L. Sherwood, R. Pawlosky, S. Ito and D. L. O'Connor (2006). "[6S]-5-Methyltetrahydrofolate is at least as effective as folic acid in preventing a decline in blood folate concentrations during lactation." Am J Clin Nutr **83**(4): 842-850.

Houghton, L. A., J. Yang and D. L. O'Connor (2009). "Unmetabolized folic acid and total folate concentrations in breast milk are unaffected by low-dose folate supplements." Am J Clin Nutr **89**(1): 216-220.

Hustad, S., O. Midttun, J. Schneede, S. E. Vollset, T. Grotmol and P. M. Ueland (2007). "The methylenetetrahydrofolate reductase 677C-->T polymorphism as a modulator of a B vitamin network with major effects on homocysteine metabolism." Am J Hum Genet **80**(5): 846-855.

Jacques, P. F., A. G. Bostom, R. R. Williams, R. C. Ellison, J. H. Eckfeldt, I. H. Rosenberg, J. Selhub and R. Rozen (1996). "Relation between folate status, a common mutation in methylenetetrahydrofolate reductase, and plasma homocysteine concentrations." Circulation **93**(1): 7-9.

Kalmbach, R. D., S. F. Choumenkovitch, A. M. Troen, R. D'Agostino, P. F. Jacques and J. Selhub (2008). "Circulating folic acid in plasma: relation to folic acid fortification." Am J Clin Nutr **88**(3): 763-768.

Kalmbach, R. D., S. F. Choumenkovitch, A. P. Troen, P. F. Jacques, R. D'Agostino and J. Selhub (2008). "A 19-base pair deletion polymorphism in dihydrofolate reductase is associated with increased unmetabolized folic acid in plasma and decreased red blood cell folate." J Nutr **138**(12): 2323-2327.

Kennedy, D. A., S. J. Stern, I. Matok, M. E. Moretti, M. Sarkar, T. Adams-Webber and G. Koren (2012). "Folate Intake, MTHFR Polymorphisms, and the Risk of Colorectal Cancer: A Systematic Review and Meta-Analysis." J Cancer Epidemiol **2012**: 952508.

Kim, T. H., J. Yang, P. B. Darling and D. L. O'Connor (2004). "A large pool of available folate exists in the large intestine of human infants and piglets." J Nutr **134**(6): 1389-1394.

Koletzko, B., C. P. Bauer, A. Brönstrup, M. Cremer, M. Flothkötter, C. Hellmers, M. Kersting, M. Krawinkel, H. Przyrembel, T. Schäfer, K. Vetter, U. Wahn and A. Weißenborn (2013). "Säuglingsernährung und Ernährung der stillenden Mutter. Aktualisierte Handlungsempfehlungen des Netzwerks Junge Familie, ein Projekt von IN FORM." Monatsschr Kinderheilkd **161**: 237-246.

Lamers, Y., R. Prinz-Langenohl, S. Bramswig and K. Pietrzik (2006). "Red blood cell folate concentrations increase more after supplementation with [6S]-5-methyltetrahydrofolate than with folic acid in women of childbearing age." Am J Clin Nutr **84**(1): 156-161.

Liu, J., L. B. Hesson, A. P. Meagher, M. J. Bourke, N. J. Hawkins, K. N. Rand, P. L. Molloy, J. E. Pimanda and R. L. Ward (2012). "Relative distribution of folate species is associated with global DNA methylation in human colorectal mucosa." Cancer Prev Res (Phila) **5**(7): 921-929.

Matthews, R. G. and C. M. Baugh (1980). "Interactions of pig liver methylenetetrahydrofolate reductase with methylenetetrahydropteroylpolyglutamate substrates and with dihydropteroylpolyglutamate inhibitors." Biochemistry **19**(10): 2040-2045.

Molloy, A. M. and J. M. Scott (1997). "Microbiological assay for serum, plasma, and red cell folate using cryopreserved, microtiter plate method." Methods Enzymol **281**: 43-53.

Niesser, M., H. Demmelmair, T. Weith, D. Moretti, A. Rauh-Pfeiffer, M. van Lipzig, W. Vaes, B. Koletzko and W. Peissner (2013). "Folate Catabolites in Spot Urine as Non-Invasive Biomarkers of Folate Status during Habitual Intake and Folic Acid Supplementation." Plos One **8**(2).

Obeid, R., M. Kasoha, S. H. Kirsch, W. Munz and W. Herrmann (2010). "Concentrations of unmetabolized folic acid and primary folate forms in pregnant women at delivery and in umbilical cord blood." Am J Clin Nutr **92**(6): 1416-1422.

Ohrvik, V. E., B. E. Buttner, M. Rychlik, E. Lundin and C. M. Witthoft (2010). "Folate bioavailability from breads and a meal assessed with a human stable-isotope area under the curve and ileostomy model." Am J Clin Nutr **92**(3): 532-538.

Patanwala, I., M. J. King, D. A. Barrett, J. Rose, R. Jackson, M. Hudson, M. Philo, J. R. Dainty, A. J. Wright, P. M. Finglas and D. E. Jones (2014). "Folic acid handling by the human gut: implications for food fortification and supplementation." Am J Clin Nutr **100**(2): 593-599.

Pentieva, K., H. McNulty, R. Reichert, M. Ward, J. J. Strain, D. J. McKillop, J. M. McPartlin, E. Connolly, A. Molloy, K. Kramer and J. M. Scott (2004). "The short-term bioavailabilities of [6S]-5-methyltetrahydrofolate and folic acid are equivalent in men." J Nutr **134**(3): 580-585.

Perez-Conesa, D., J. F. Haro-Vicente, F. R. Braquehais and G. Ros (2009). "[6S]-5-Methyltetrahydrofolate enhances folate status in rats fed growing-up milk." Eur J Nutr **48**(6): 365-371.

Prinz-Langenohl, R., S. Bramswig, O. Tobolski, Y. M. Smulders, D. E. Smith, P. M. Finglas and K. Pietrzik (2009). "[6S]-5-methyltetrahydrofolate increases plasma folate more effectively than folic acid in women with the homozygous or wild-type 677C-->T polymorphism of methylenetetrahydrofolate reductase." Br J Pharmacol **158**(8): 2014-2021.

Salmenpera, L., J. Perheentupa and M. A. Siimes (1986). "Folate nutrition is optimal in exclusively breast-fed infants but inadequate in some of their mothers and in formula-fed infants." J Pediatr Gastroenterol Nutr **5**(2): 283-289.

Scientific Committee on Food, E. C., prepared by Koletzko, B., Saris, W., Flynn, A., Palou, A., Wal, J.M., Hernell, O., Jackson, A., Przyrembel, H., Turck, D. (2003). Report of the Scientific Committee on Food on the Revision of Essential Requirements of Infant Formulae and Follow-on Formulae. Brussels, European Commission.

Troen, A. M., B. Mitchell, B. Sorensen, M. H. Wener, A. Johnston, B. Wood, J. Selhub, A. McTiernan, Y. Yasui, E. Oral, J. D. Potter and C. M. Ulrich (2006). "Unmetabolized folic acid in plasma is associated with reduced natural killer cell cytotoxicity among postmenopausal women." J Nutr **136**(1): 189-194.

Vanhees, K., I. G. Vonhogen, F. J. van Schooten and R. W. Godschalk (2014). "You are what you eat, and so are your children: the impact of micronutrients on the epigenetic programming of offspring." Cell Mol Life Sci **71**(2): 271-285.

West, A. A., J. Yan, C. A. Perry, X. Jiang, O. V. Malysheva and M. A. Caudill (2012). "Folate-status response to a controlled folate intake in nonpregnant, pregnant, and lactating women." Am J Clin Nutr **96**(4): 789-800.

APPENDICES

1. AE Form
2. SAE Form
3. SAE Form Completion Guideline
4. SAE reporting flow-chart
5. Informed Consent Form & Parent Information
6. Questionnaires
7. SOP infant baseline and V4
8. SOP mother
9. SOP infant V5

Adverse Event (AE) — Running Log

Study ID N°: 2014-05-06-MTHF

Investigational Product: HiPP Fol-SN

Protocol Name: Suitability of an infant formula with L-5-Methyltetra-hydrofolate for the particular nutritional use in infants

NOTE: Do not report SAEs in this AE Running Log; enter SAEs on separate SAE Form.

Site Name/N°	Enrollment N°/Subject ID	Subject Initials	Running Pagination
	☐☐☐☐☐☐	☐☐☐	Page: ___________ of ___________

No Adverse Event Reported ☐

Adverse Events	Onset Date	Stop Date	If < 24 hours duration provide time:	Intensity	Frequency	Relationship	Action Taken	Outcome
	___/____/___ _ dd mmm yyyy	___/____/___ _ dd mmm yyyy	___:___ hr min	1 Mild<br>2 Moderate<br>3 Severe<br>4 Life-threatening<br>5 Death	1 Once<br>2 Several Times<br>3 Ongoing	1 Not Related<br>2 Related<br>3 Suspected	1, 2, 3, 4, 5+	1, 2, 3, 4, 5*

____

Signature Primary Investigator / Designate Date

+Action Taken: 1 No Action Taken; 2 Regimen Adjusted / Interrupted; 3 Regimen Discontinued; 4 Concomitant Medication; 5 Non-Drug Therapy
*Outcome: 1 Recovered; 2 Recovered with sequelae; 3 Ongoing; 4 Fatal; 5 Unknown / Lost to Follow-up

SERIOUS ADVERSE EVENT (SAE) FORM (PAGE 1 OF 2)

STUDY NUMBER	INVESTIGATIONAL SITE N° / NAME	SUBJECT NUMBER	SUBJECT INITIALS

REPORT TYPE
☐ Initial ☐ Follow-up N°______ ☐ Final/Closed

COUNTRY

DATE OF BIRTH (dd mmm yyyy)

SEX
☐ Male
☐ Female

HEIGHT
☐ cm
☐ ft/in

WEIGHT
☐ kg
☐ lb

SERIOUS ADVERSE EVENT (DIAGNOSIS IF POSSIBLE)

ONSET DATE (dd mmm yyyy)

END DATE (dd mmm yyyy)

DESCRIPTION OF EVENT
(brief chronological summary of signs, symptoms, treatment of SAE, relevant investigational findings, etc.)

(Please continue on second page)

INTENSITY
☐ Mild (Grade 1)
☐ Moderate (Grade 2)
☐ Severe (Grade 3)
☐ Life-threatening (Grade 4)
☐ Death (Grade 5)

RELATION TO PRODUCT
☐ Related
☐ Not Related
☐ Suspected

SERIOUSNESS CRITERIA
☐ Death
☐ Life-threatening
☐ Involved or prolonged in-patient hospitalisation

Admission date: |_|_| |_|_|_| |_|_|_|_|
dd mmm yyyy

Discharge date: |_|_| |_|_|_| |_|_|_|_|
dd mmm yyyy

☐ Congenital anomaly / birth defect
☐ Persistent or significant disability / incapacity
☐ Medically significant

OUTCOME OF EVENT
☐ Recovered
☐ Recovered with sequelae
☐ Ongoing
☐ Fatal (date of death)
|_|_| |_|_|_| |_|_|_|_|
dd mmm yyyy
-> Autopsy performed:
☐ Yes ☐ No
☐ Unknown / Lost to follow-up

ACTION TAKEN
☐ None
☐ Regimen Adjusted / Interrupted:
☐ Dose changed:_______
☐ Temporarily interrupted
|_|_| |_|_|_| |_|_|_|_|
dd mmm yyyy
☐ Reintroduced
|_|_| |_|_|_| |_|_|_|_|
dd mmm yyyy
☐ Permanently discontinued
☐ Drug-therapy given
If yes, list in section 'Description of the event'.
☐ Non-drug therapy given
If yes, list in section 'Description of the event'.

INVESTIGATIONAL PRODUCT

BATCH / LOT NUMBER

BREAKING OF CODE
☐ Yes ☐ No ☐ N/A
If Yes: Investigational product:....

FIRST ADMINISTRATION (dd mmm yyyy)	LAST ADMINISTRATION (dd mmm yyyy)	DAILY DOSE	ROUTE	INVESTIGATIONAL PRODUCT APPLICATION FIELD

TIME ELAPSED BETWEEN LAST PRODUCT ADMINISTRATION AND ONSET OF FIRST SIGNS / SYMPTOMS OF SAE: |_|_| minutes |_|_| hours |_|_| days |_|_| months

DID EVENT ABATE AFTER DISCONTINUATION OF INVESTIGATIONAL PRODUCT?
☐ Yes ☐ No ☐ N/A

DID EVENT REAPPEAR AFTER REINTRODUCTION?
☐ Yes ☐ No ☐ N/A

SERIOUS ADVERSE EVENT (SAE) FORM (PAGE 2 OF 2)

STUDY NUMBER	INVESTIGATIONAL SITE N^{O} / NAME	SUBJECT NUMBER	SUBJECT INITIALS
REPORT TYPE<br>☐ Initial ☐ Follow-up N^{o}______ ☐ Final/Closed		COUNTRY	

STUDY NUMBER	INVESTIGATIONAL SITE N^{O} / NAME	SUBJECT NUMBER	SUBJECT INITIALS

CONCOMITANT DRUGS OR PRODUCTS

	DRUG / PRODUCT	TOTAL DAILY DOSE	ROUTE	INDICATION	START (dd mmm yyyy)	END (dd mmm yyyy)
1						
2						
3						
4						
5						
6						

DESCRIPTION OF EVENT (CONTINUED):

CONCOMITANT DISEASES / RELEVANT MEDICAL HISTORY

RELEVANT TESTS AND LABORATORY FINDINGS (RELEVANT FOR SAE DIAGNOSIS, INCLUDE REFERENCE UNITS AND RANGES)

ADDITIONAL INFORMATION

REPORTER'S NAME:<br><br>DATE (dd mmm yyyy)	ADDRESS:<br><br>TELEPHONE:	RECIPIENT'S NAME:

SAE Form Completion Guideline

SAE FORM COMPLETION GUIDELINE

GENERAL INSTRUCTIONS

- Complete an SAE Form for any Serious Adverse Event — whether or not the SAE is deemed product-related, occurring during the study period from the date of signature of the informed consent form up to and including the protocol-specified follow-up period after the last dose of investigational product.
  (note: Protocols should define AEs, SAEs, and SUSARs and also the SAE follow-up period)
- The SAE Form should include the following minimum information: subject initials, subject number, serious adverse event, seriousness criteria, investigational product, SAE relationship to investigational product and Reporter's information.
- The SAE Form will be completed and reported to United BioSource Corporation (UBC) Geneva by fax (+800 24 25 26 27 (from EU only or +1 877 200 2922 (from USA only) or +41 22 596 44 46) or email (EUsafety@ubc.com) within 24 hours regardless of whether all information is known.
- Any additional information that becomes available after the initial SAE Form has been submitted should be reported in a Follow-up SAE Form as soon as possible. The SAE Follow-up report must include the minimum information described above, but only changed or new information needs to be specified.
- Information provided in the Follow-up SAE Form supersedes the information initially reported in the previous version(s) of the SAE Report Form.
- Complete all sections legibly using a blue or black ink pen.
- Leave no section blank. If information is unknown at the time of the report, write "Unknown (Unk);" otherwise use "Not applicable (N/A)," as appropriate.
- Complete all sections in English; avoid using slang or non-medical terminology.
- Use only universally accepted medical abbreviations, acronyms and/or symbols.
- All dates should be completed using the international date format: day/month/year (dd-mmm-yyyy) e.g. 18-SEP-2007.
- Use the 24-hour clock for time, e.g., 23:29.
- When making a correction, strike-through the incorrect information with one line and initial and date the correction. Do not use correction fluid or scratch out the information.

DEFINITIONS

- **Serious Adverse Event (SAE):**

Any adverse event that at any dose fulfills at least one of the following criteria:

- Is fatal (results in death)
  (*note*: death is an outcome, not an event)
- is life-threatening
  (*note*: the term "life-threatening" refers to an event in which the patient was at risk of death at the time of the event; it does not refer to an event which could hypothetically have caused death had it been more severe)
- required inpatient hospitalisation or prolongation of existing hospitalisation

(*note*: "inpatient hospitalisation" refers to an unplanned, overnight hospitalisation)

- results in persistent or significant disability/incapacity
(*note:* the term means substantial disruption of one's ability to conduct normal life function)
- is a congenital anomaly/birth defect
(*note:* congenital anomaly/birth defect in offspring of subject taking the product regardless of time to diagnosis)
- is medically significant
(*note*: Medical and scientific judgment should be exercised in deciding whether expedited reporting is appropriate in other situations, such as important medical events that may not be immediately life-threatening or result in death or hospitalisation but may jeopardize the patient or may require intervention to prevent one of the other outcomes listed in the definition above).

REMINDER: Intensity (*mild, moderate, severe*) is different from Seriousness!

- **Causal relationship:**

**Not Related:**	The temporal relationship of the clinical event to the investigational product makes a causal relationship unlikely, or other drugs, therapeutic interventions or underlying conditions provide a sufficient explanation for the observed suspected event.
**Related:**	The temporal relationship of the clinical event to investigational product makes a causal relationship possible, and other drugs, therapeutic interventions or underlying conditions do not provide a sufficient explanation for the observed event.
**Suspected:**	There is a reasonable possibility that the product caused the observed event. Suspected implies a lesser degree of certainty about the causality of the observed event.

INSTRUCTIONS FOR COMPLETING FORM

General information

- Enter the Study number, Investigational site number or name, Subject number, Subject initials on all pages of the SAE form.
- Report Type: Tick the appropriate box to indicate the type of report being submitted, i.e., initial, follow-up, final/closed.
- Enter the country of occurrence

Subject information

- Complete all subject information including date of birth, age, sex, height and weight (specify the unit for height and weight by ticking the appropriate box).

SAE FORM COMPLETION GUIDELINE

Adverse Event information

- Provide the Serious Adverse Event term. The investigators should make every effort to provide a diagnosis if possible. The SAE term should describe the symptom(s) or diagnosis, not a procedure. For example, if the subject was hospitalised for a surgical procedure, such as a cholecystectomy, the reason for the surgery, “pain due to Gall bladder,” should be reported as the SAE term. Another example is hospitalisation due to “severe stomach pain.” The term “severe stomach pain” should be recorded until a diagnosis, such as “duodenal ulcer,” is determined.
- Enter SAE onset date (date of first occurrence of the SAE sign(s) and symptom(s)) and end date (date of last occurrence of the SAE sign(s) and symptom(s)).

Description of Event

- Provide a clear description of the sequence, nature and course of events, including details of treatment(s) (drug and non-drug), action taken and the outcome of the SAE. If the subject was hospitalised or hospitalisation was prolonged, state the reasons clearly. Include any information which helps in understanding the SAE.
- This section continues on page 2 of the SAE report.

Intensity of SAE

- Tick the appropriate box for the degree of severity
- If there is more than one symptom/diagnosis with different degrees of severity, enter the worst degree of intensity that occurred for that symptom/diagnosis.
- Mild (Grade 1): easily tolerated and does not interfere with daily activity.
- Moderate (Grade 2): interferes with daily activity, but the subject is still able to function.
- Severe: (Grade 3) incapacitating and requires medical intervention.
- Life-threatening (Grade 4)
- Death (Grade 5)

Relationship to Product

- **Not related:** There is no reasonable possibility that the investigational product may have caused or contributed to the occurrence of the adverse event.
- **Related:** There is a reasonable possibility that the investigational product may have caused or contributed to the occurrence of the adverse event.
- **Suspected:** There is a reasonable possibility that the investigational product caused the observed event. Suspected implies a lesser degree of certainty about the causality of the observed event.

Seriousness criteria

- Indicate seriousness criteria by checking the appropriate box. If there is more than one seriousness criterion, tick all the appropriate boxes (e.g. for hospitalization and death, tick both boxes).
- Provide the hospital admission and discharge dates if applicable.

SAE FORM COMPLETION GUIDELINE

Outcome

- Tick the appropriate outcome.
- If the patient recovered with sequelae, specify the sequelae in the description of the event.
- Enter the date of death if the outcome was fatal.
- If an autopsy was performed, check the Yes box and describe results in the "additional information" section.
- If the outcome is unknown, or the subject is lost to follow-up, tick the appropriate box.

Action Taken

- Tick the appropriate box for the action taken.
- If the investigational product dosage was adjusted, provide details of the new dosage.
- If the investigational product was temporarily interrupted, enter the date the investigational product was stopped and then the date it was restarted.
- If drug therapy was given to treat the SAE, specify it in the section Description of the event.
- If non-drug therapy was given to treat the SAE, specify it in the section Description of the event.

Investigational product

- Complete the product information including product name (open study) and batch or lot number if applicable.
- Enter the start and stop dates, dose, route of administration and indication of the investigational drug.
- Enter the time elapsed between the last product administration and the onset of the first signs/symptoms of the SAE in months, days, minutes and/or hours.
- In a double-blind study, indicate if the code was broken or not by ticking the appropriate box. If the code was broken, enter the name of the investigational product. Note: Please only break the blind in case of a medical emergency.
- Did the event abate after product discontinuation?: Tick "YES", "NO" or "N/A".
- Did the event reappear after reintroduction?: Tick "YES", "NO" or "N/A".

Concomitant Drugs or Products

- Provide a list of concomitant medications taken prior or at the time of the SAE, including name of the drug/product (generic/brand name), total daily dose, route of administration, indication and start and end dates of administration.
- Medications used to treat the SAE should be reported in the section "Description of event".

Concomitant diseases/Relevant Medical History

- Provide a clear description of the subject's medical history.

Relevant Tests and Laboratory Findings

- Provide a list of tests which are relevant for SAE diagnosis, e.g., laboratory findings.
- Include reference units and normal ranges

SAE FORM COMPLETION GUIDELINE

Additional information

- Provide any additional information which could help in the understanding of the case, e.g., patient's family history, results of autopsy, non-drug therapy, etc.

Investigator Information

- Provide Reporter's information including name, complete address and telephone number.
- Enter the date of the report and the recipient's name.

Fax or scan/email the completed SAE Form incl. all pages to:

UBC Safety Europe

**Fax number**	**+41 22 596 44 46**
**Toll free fax number**	**+800 24 25 26 27 (from EU only)**
**Toll free fax number**	**+1 877 200 2922 (from USA only)**

E-mail: EUsafety@ubc.com

SAE FLOW-CHART

Product: L-5-Methyltetrahydrofolate
Protocol Title: Suitability of an infant formula with L-5-Methyltetrahydrofolate for the particular nutritional use in infants.
Protocol Number: 2014-05-06-MTHF

Mono-centric, randomized, double-blind, parallel-group, controlled prospective intervention study in healthy infants.

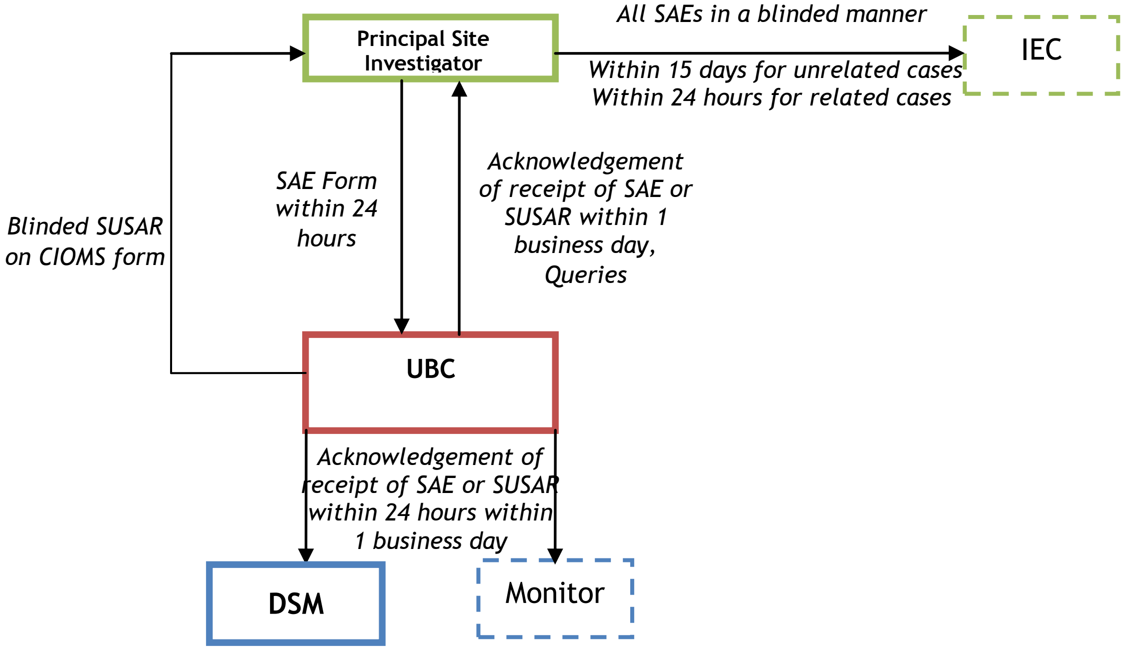

The Principal Site Investigator must report all SAEs to PV vendor (UBC) within 24 hours of becoming aware of the event, whether or not the SAE is considered to be related to the study supplementation.
Every SAE will be reported to the IEC in a fully blinded manner:

- **within 15 days if not related to the study;**
- **within 24 hours if related.**

No SUSAR reporting is required to the IEC. AE annual line listing to the IEC needed.

<u>**Contact Persons:**</u>

United BioSource Corporation **Safety Fax:+41 22 596 44 46**
+800 24 25 26 27 (from EU only)
EUsafety@ubc.com

Mallorie Clement Phone: **+ 41 22 596 44 22**
Associate Director, Pharmacovigilance Email: mallorie.clement@ubc.com

SAE FLOW-CHART

Francine Galibert
Senior Safety Scientist

Phone: +41 22 939 41 57
Email: francine.galibert@ubc.com

DSM

Dr Szabolcs Péter, MD, PhD
Study Director

Phone: +41 61 815 8966
Email: Szabolcs.peter@dsm.com

Dr Stephanie Krammer-Lukas
Project Manager

Phone: +41 61 815 8437
Email: Stephanie.krammer-lukas@dsm.com

Investigator's Team

Prof. Berthold Koletzko
Univ.- Professor
Principal Investigator

Fax: +49 89 8396 4044
Phone: +49 895160 3967
E-mail: Berthold.koletzko@med.lmu.de

Prim. Dr. Milica Vusurovic
Principal Site Investigator

Fax: -
Phone: +381 (0) 63 312 171
E-mail: milica.vusurovic@gmail.com

Monitor

Milana Papović
CRA
Sermon CRO
Resavska 12/4
11000 Belgrade, Serbia

Fax: +381 11 33 44 255
Phone: +381 11 33 44 255, +381 11 6303 684
Mobile: +381 62 256 630
E-mail: milana.papovic@sermoncro.com

Ana Gasparovic
Project Manager
Sermon CRO
Donje Svetice 46 C/V
10000 Zagreb, Croatia

Fax: +385 1 23 34 616
Phone: +385 1 550 92 47
E-mail: ana.gasparovic@sermoncro.com

Reference document
Investigator's Brochure.

Unblinding procedure
- The site will receive sealed code envelopes
- Code envelopes will be provided by DSM/Investigator to DSM PV Vendor (UBC).

Date: 15-Jan-15

Informed Consent

Title of study:	Suitability of an infant formula with L-5-Methyltetra-hydrofolate for the particular nutritional use in infants
Study code (short name, acronym):	2014-05-06-MTHF, MEFOLIN
Version / Date:	Version 1.0 of 11.12.2014
Head of Study:	Prof. Dr. Berthold Koletzko
Local Head of Study:	Prim. Dr. Milica Vusurovic

Please read the Parents Information and the Informed Consent very carefully.
Please do not hesitate to ask if anything is unclear or if you would like to have further information.

I/we hereby declare my/our consent to allowing my/our child

____
name of mother

____________________________________ □□/□□/□□□□
name of child date of birth (day / month / year)

to take part in the aforementioned study.

I/we have received the Parents Information about the Study, read it and understood it and am/are therefore informed about the study. I/we have no further questions.

I/we have particularly been informed
- that the study involves research
- that participation is voluntary and not linked to the provision of other medical care at this or other care providers
- that this study aims to compare growth and development of healthy infants fed with two different infant formula (containing a B-vitamin in one of two forms, 5-methyltetrahydrofolate [L-5MTHF] or folic acid, respectively) and breast milk as reference
- that it is potentially beneficial to provide L-5MTHF rather than folic acid with infant formula to achieve a supply more similar to those of the breast fed infants
- that in total approximately 360 subjects will be involved in the study
- that the expected duration of the subject's participation is from birth until 1 year of life
- that my child will attend 6 study visits for medical check ups
- that 2.6 ml blood will be taken from the infant at the beginning of the study, 2.6 ml at the infant age of 16 weeks and 2 ml at the infant age of 1 year
- that data about nutrition, well-being, sleeping and crying and digestion will be collected via diaries filled-in by the parents
- that from breastfeeding mothers a blood and a breast milk sample will be drawn at the infant age of 8 weeks
- that only for explorative research purposes blood cells will be stored for eventual later anonymous genetic analyses

To be filled in by the study staff:

Subject ID □□□□□

- that the risk of the blood collection is that of a small local hematoma, or an infection and the study intervention or procedure may involve risks to the subject which are currently unforeseeable
- that it is advised to preferably provide exclusive breastfeeding for infants during the first half of the first year of life
- that the data collected in the context of this study are intended and used exclusively for study purposes
- that records identifying the subject will be kept confidential and, to the extent permitted by the applicable law and regulations, will not be made publicly available
- that the monitor(s), the auditor(s), the Ethical Committee, and the regulatory authorities will be granted direct access to the subject's original medical records for verification of clinical trial procedures and/or data, without violating the confidentiality of the subject, to the extent permitted by the applicable laws and regulations
- that I/we will be informed in a timely manner if new information becomes available that may be relevant to the subject parents' willingness to continue participation in the study
- that I/we can contact Prim. Dr. Milica Vusurovic for answers to pertinent questions about the research, research subjects' rights, and in the event of a research-related injury to the subject
- that the investigator has the right to withdraw subjects from the study if this is in the best interest of the subject (e.g. erroneous inclusion, adverse event or concurrent illness, intake of prohibited concomitant medication/dietary supplements, parents / caregivers of subjects who do not follow the requirements of the investigator, request by regulatory agencies etc.)

I/we have been informed about the blood samples to be taken in the course of the study and agree to such procedure.

I/we have been informed that I/we can ask further questions at any time and that we can withdraw our consent to participate in the MEFOLIN Study at any time – without penalty and without having to state any reasons.

Place, date	Signature of 1st person having custody of child
Place, date	Signature of 2nd person having custody of child

Persons with single custody
I confirm that I have single custody and I consent to the participation of my child.

Place, date	Signature of person having custody of child

I have informed the participant verbally and in writing on the objective, duration, benefits, risks and side-effects of the study. I have answered any resulting questions comprehensibly and sufficiently. The person/s having custody of the child has/have given his/her/their consent voluntarily without compulsion. I have handed out a copy of the written Parents Information and this Declaration of Consent to the person/s having custody of the child.

Name of the explaining physician in block letters	Signature of the explaining physician	Date (by own hand)

Parent Information

Title of study:	Suitability of an infant formula with L-5-Methyltetra-hydrofolate for the particular nutritional use in infants
Study code (short name, acronym):	2014-05-06-MTHF, MEFOLIN
Version / Date:	Version 1.0 of 11.12.2014
Head of Study:	Prof. Dr. Berthold Koletzko
Local Head of Study:	Prim. Dr. Milica Vusurovic

You have been invited to take part in a research study comparing growth and development of healthy infants fed with two different infant formula and breast milk as reference. In total 360 subjects will be recruited to the study, the expected duration of the subject's participation is from birth until 1 year of life. The study was reviewed by the local Ethical Committee in Belgrade. Before you decide to participate please take time to read the following information carefully. Please ask us if there is anything that is not clear or if you would like more information.

It is up to you to decide whether to take part or not. If you decide to take part you will be given this information sheet to keep and asked to sign a consent form. You will be free to withdraw at any time and without giving a reason.

Purpose of the study

It appears potentially beneficial to provide L-5-Methyltetrahydrofolate (L-5MTHF, the form of folate found in human breast milk) rather than folic acid with infant and follow-on formula to achieve a supply of the natural form of folate and to avoid potential adverse effects, but no data from clinical studies in infants are available. This study aims to compare growth and development of healthy infants fed with two different infant formula (containing L-5MTHF and folic acid, respectively) and breast milk as reference.

This study is part of a dossier to be sent to the European Commission for admission of adding L-5MTHF to infant and follow on formula.

WHO CAN PARTICIPATE?

Eligible for participation in the project are healthy breastfed and non-breastfed newborn infants, whose parents have given their written consent to their participation and who

- were born healthy and mature (≥ 37 weeks of gestation)
- weighed between 2,500 and 4,500 grams at birth
- are not older than 27 days (approx. 4 weeks) at the beginning of the study
- are either fully breastfed or fully formula fed at day 27 after birth

Study products

Control product is an infant formula following current standards as laid down in the European legislation for infant formulae, which contains 10 µg of the B-vitamin folic acid per 100 ml reconstituted infant formula.

The intervention product is an infant formula with similar composition as the control product, except for including 13.0 µg of the B-vitamin L-5MTHF per 100 ml reconstituted infant formula (instead of folic acid).

The control product and the intervention product are as similar as technically feasible in all compositional aspects except for the source of folate. Breast milk will serve as reference.

Your baby will be allocated to one of the two formula groups by chance (randomisation), according to a list which is generated by a computer. The study formula will be packed in identical white 500g-boxes and named “HiPP Fol-SN” (infant formula). Until the end of the study neither you nor the paediatrician know which formula is given to which child (it’s called double-blind randomized study). This procedure is necessary to ensure that the results of the study are not influenced by anyone. Every single packet is provided with a code number. If necessary, this can be used to immediately identify which formula your child is receiving.

Mothers are being advised to preferably provide exclusive breastfeeding to their infant during the first half of the first year of life. In case you decide to stop exclusively breastfeeding within the first 4 weeks of life – due to any reason not related to this study – you can contact the study team and get study infant formula for free. Your infant will be randomized into one of the study formula groups.

HOW OUR PROJECT WILL PROCEED

Duration of participation: 12 months
The project starts for your child at age 0-4 weeks. Phase 1 lasts 12 weeks (4th to 16th week of life). This is followed directly by Phase 2 leading up to the end of your baby’s first year of life. In total, 360 children from Serbia will be taking part.

Nutrition during Phase 1 (4th to 16th week of life)
Breast milk: During the first months of life, breastfeeding is best for your baby! That is why we recommend that you exclusively breastfeed your child if you can.
Supplementary nutrition: Formula supplementation is only necessary if you have decided not to fully breastfeed your infant, if you are unable to breastfeed or if you are unable to produce a sufficient amount of breast milk. If this is the case with your child during the first four weeks of your baby’s life, we would be happy to invite you to join the formula arm of this study and provide formula free of charge. If you require infant milk formula, please let us know. You will find the contact details on the last page.
Jars, paps and beverages: In accordance with the local recommendations, the introduction of complementary foods (ie, solid foods and liquids other than breast milk or infant formula and follow-on formula) should not be before 17 weeks but should not be delayed beyond 26 weeks.

Nutrition during Phase 2 (17th to 52nd week of life)
From the 17th to 52nd week of life, you are encouraged to follow the local feeding recommendations. We recommend that you introduce solid food step by step starting when your baby is at least 17 weeks old but not older than 26 weeks, and stepwise reduce the amount of breast milk or infant milk formula, respectively.

Medical check-ups
As part of the project, we will monitor and observe your child’s development very carefully. Besides the appointment at the beginning of the study (Baseline Visit) for description of the procedure, assessment of growth dates, provision of infant milk formula and diaries, you will have a total of 5 appointments. They are scheduled in the 4th, 8th, 12th, 16th and 52nd week for medical check-ups lasting approx. 60 minutes each at the study centre. We will examine your child specifically regarding growth, normal thriving and well-being.

At 3 appointments (the Baseline Visit, Visit 1 at 16th and Visit 5 at 52nd week) blood samples will be taken (~ 2.6 ml, ~ 2.6 ml and ~ 2 ml, respectively). This helps us to assess your child's nutritional state optimally and to assess the status of folate.
In the breastfeeding group at Visit 2 the mothers` folate status will be evaluated by taking a blood sample and a breast milk sample from the breastfeeding mother.

The blood sample is taken by experienced paediatric nurses, and local anaesthetic ointment can be used to prevent pain in the infant.

You will not be charged for the medical examinations, they are free. Please note that these examinations do not in any way replace the routine which are conducted by your paediatrician.

During the project, you will be asked to fill in diaries in certain intervals (three days before each visit). In this diaries you will be asked about drinking amounts, well-being, crying and sleeping of your baby as well as about your baby´s digestion.
We would like to ask you to use the diaries to record information on nutrition etc. and anything unusual you may notice about your child's health.

Are there risks?

The risks involved in participating in this study are minimal. The use of L-5-MTHF in this infant growth and tolerance study is not of concern from a safety point of view. L-5-MTHF has been evaluated and considered safe in human adults, and it is used in drugs, supplements and food products in many countries around the world. The risk of the blood collection is merely that of a small local hematoma, or an infection. You will be informed in a timely manner if new information becomes available that may be relevant to your willingness to continue participation in the study. Examples of such new information include, but are not limited to, increase in the known risks to study subjects, additional safety information from other preclinical or clinical studies.

Benefits

The supplementation of L-5MTHF (the form of folate found in human breast milk instead of folic acid) to infants fed with infant formulas may help to increase L-5-MTHF and reduce folic acid blood levels which have been associated with potential adverse effects. Thus, the blood L-5MTHF levels of bottle fed infants may become more similar to those of the breast fed population.

Insurance

Sponsor`s liabilities in connection with the study will be covered by an insurance policy (Policy No. 07 00030993 4) in the event of a subject suffering any significant injury, which is proven as being as a direct result of study participation.

Compensation

Travel expenses for participation in study visits may be reimbursed to participating families, but no payment is offered for study participation. After the intervention phase, vouchers will be offered for parents of all study groups which can be exchanged for e.g. food supplements or hygienic products.

Participation

Participation in the study is voluntary and you may refuse to participate in or may withdraw from the study, any time, without penalty, and without need to state the withdrawal reason

Confidentiality

Records identifying the subject will be kept confidential and, to the extent permitted by the applicable law and regulations, will not be made publicly available; and if the results of the trial are published, the subjects identity will remain confidential. The monitor(s), the auditor(s), the Ethical Committee, and the regulatory authorities will be granted direct access to the subject's original medical records for verification of clinical trial procedures and/or data, without violating the confidentiality of the subject, to the extent permitted by the applicable laws and regulations.

Contact

For further information or for answers to questions about the study, subjects' rights, and in the event of a research-related injury to the subject you can contact:

Prim. Dr. Milica Vusurovic

KBC "Dr Dragiša Mišović-Dedinje"
Neonatology
Heroja Milana Tepića 1, 11000 Belgrade, Serbia
+381 (0) 63 312 171
milica.vusurovic@gmail.com

Termination of participation in the study

You have the right to withdraw from the study at any time for any reason, without being obliged to give reasons and without penalty or loss of benefits you are entitled to. The investigator also has the right to withdraw subjects from the study if this is in the best interest of the subject. Any of the following conditions leads to premature withdrawal:

- request by parents / caregivers to discontinue for any reason during the study
- erroneously included / randomized subject
- adverse event or concurrent illness that, in the opinion of the investigator, warrants the subject's withdrawal from intervention
- intake of prohibited concomitant medication/dietary supplements
- parents / caregivers of subjects who do not follow the requirements of the investigator, especially those concerning safety and/or if the parents / caregivers of subjects after the enrolment are not willing to comply with the protocol
- failure to return
- request by regulatory agencies for termination of supplementation of an individual subject or all subjects under this protocol

Thank you for reading this information sheet. If you decide to take part in this study you will be given a copy of the information sheet and a signed consent form to keep.

3-DAY DIARY FOR BREASTFEEDING MOTHERS

**3-day Diary for Breastfeeding Mothers**	*"Suitability of an infant formula with L-5-Methyltetra-hydrofolate for the particular nutritional use in infants"*	**2014-05-06-MTHF**
Period before: __ (e.g. V2)<br>*(visit every 4 weeks)*		**Subject ID: S _ _ _ _**

Contact Details:

Enrollment and clinical examinations:<br>**Hipp** GmbH und Co. Vertrieb KG	Recruitment:<br><br>**KBC "Dr Dragiša Mišović-Dedinje"**	CRO:<br><br>**Sermon**
**Clinical Study Center**, Belgrade<br>Kneza Milosa 51, Belgrade, Serbia<br><br>+381 (0) 11 3612 945<br>+381 (0) 60 51 50 530<br>hipp@technicom.net	Neonatology<br>Heroja Milana Tepića 1,<br>11000 Belgrade,<br>Serbia<br><br>+381 (0) 63 312 171<br>milica.vusurovic@gmail.com	Donje Svetice 46 C/V,<br>10000 Zagreb,<br>Croatia<br><br>+385 1 550 92 47<br>+385 1 23 34 616<br>ana.gasparovic@sermoncro.com

Dear Parents / Caregiver,

Enclosed you will find a **Diary,** please take notes **three days before the visit** what else, when, how often and how much besides breast milk your infant is drinking/eating (allowed are maximal 50 ml water or tea per day). Please also record when you are applying the other drinks.

In addition we ask you to record the palatability and tolerability ("reflux and digestion symptoms" and fecal assessment) of the breast milk.

For the study conduct, it is important that you keep this **Diary**. **Please also write down if you gave him/her any medication in the remarks / comments column.**

Please bring this 3-day Diary with you to your next appointment. On this date, you will receive a new 3-day Intake Diary.

Site: Clinical Study Center, Kneza Milosa 51, Belgrade

________	________	____________
Study Day	Date	Time

For inquiries to our team, please contact us by phone (+381 (0) 63 312 171) or by e-mail (milica.vusurovic@gmail.com).

Thank you for your cooperation!

Your study team

3-DAY DIARY FOR BREASTFEEDING MOTHERS

**3-day Diary for Breastfeeding Mothers**	*"Suitability of an infant formula with L-5-Methyltetra-hydrofolate for the particular nutritional use in infants"*	**2014-05-06-MTHF**
Period before: __ (e.g. V2) *(visit every 4 weeks)*		**Subject ID: S _ _ _ _**

3-day Diary Example

Day of Life	*Date*	*Time*	*Other drinks / Remarks*
e.g.51	*DD.MM.YYYY*	*10:00*	Fever (paracetamol suppository)
	(DAY 1)	*22:00*	***30 ml tea***
		...	
e.g.52	*DD.MM.YYYY*	...	
	(DAY 2)		
e.g.53	*DD.MM.YYYY*	...	
	(DAY 3)		

Will be filled in by study team!		
	**Total other drinks volume**	**Other drinks volume / Day**
/		

3-DAY DIARY FOR BREASTFEEDING MOTHERS

3-day Diary for Breastfeeding Mothers	*"Suitability of an infant formula with L-5-Methyltetra-hydrofolate for the particular nutritional use in infants"*	2014-05-06-MTHF
Period before: __ (e.g. V2) *(visit every 4 weeks)*		Subject ID: S _ _ _ _

3-day Diary

Day of Life	*Date*	*Time*	*Other drinks / Remarks*
	(DAY 1)		
	(DAY 2)		
	(DAY 3)		

Will be filled in by study team!		
	**Total other drinks volume**	**Other drinks volume / Day**
/		

3-DAY DIARY FOR BREASTFEEDING MOTHERS

**3-day Diary for Breastfeeding Mothers**	*“Suitability of an infant formula with L-5-Methyltetra-hydrofolate for the particular nutritional use in infants”*	**2014-05-06-MTHF**
Period before: __ (e.g. V2)<br>*(visit every 4 weeks)*		**Subject ID: S _ _ _ _**

Palatability & Tolerability

of Breast Milk

Please describe the consistency, colour and odour of each bowl movement your infant has during the 3-days recording phase! The calculations will be conducted by the study team.

BOWL MOVEMENT ASSESSMENT – DAY 1

Today no stool ↑

	**Stool consistency**				**Stool colour**					**Odour**	
No.	watery	soft	modelled	hard	yellow	green	brown	grey	olive-black	normal (sourish)	smelly
**1**	↑	↑	↑	↑	↑	↑	↑	↑	↑	↑	↑
**2**	↑	↑	↑	↑	↑	↑	↑	↑	↑	↑	↑
**3**	↑	↑	↑	↑	↑	↑	↑	↑	↑	↑	↑
**4**	↑	↑	↑	↑	↑	↑	↑	↑	↑	↑	↑
**5**	↑	↑	↑	↑	↑	↑	↑	↑	↑	↑	↑
**6**	↑	↑	↑	↑	↑	↑	↑	↑	↑	↑	↑
**7**	↑	↑	↑	↑	↑	↑	↑	↑	↑	↑	↑
**8**	↑	↑	↑	↑	↑	↑	↑	↑	↑	↑	↑
**9**	↑	↑	↑	↑	↑	↑	↑	↑	↑	↑	↑
**10**	↑	↑	↑	↑	↑	↑	↑	↑	↑	↑	↑
Will be filled in by study team!											
Sum / Day											

3-DAY DIARY FOR BREASTFEEDING MOTHERS

BOWL MOVEMENT ASSESSMENT – DAY 2

Today no stool ↑

	Stool consistency				Stool colour					Odour	
No.	watery	soft	modelled	hard	yellow	green	brown	grey	olive-black	normal (sourish)	smelly
1	↑	↑	↑	↑	↑	↑	↑	↑	↑	↑	↑
2	↑	↑	↑	↑	↑	↑	↑	↑	↑	↑	↑
3	↑	↑	↑	↑	↑	↑	↑	↑	↑	↑	↑
4	↑	↑	↑	↑	↑	↑	↑	↑	↑	↑	↑
5	↑	↑	↑	↑	↑	↑	↑	↑	↑	↑	↑
6	↑	↑	↑	↑	↑	↑	↑	↑	↑	↑	↑
7	↑	↑	↑	↑	↑	↑	↑	↑	↑	↑	↑
8	↑	↑	↑	↑	↑	↑	↑	↑	↑	↑	↑
9	↑	↑	↑	↑	↑	↑	↑	↑	↑	↑	↑
10	↑	↑	↑	↑	↑	↑	↑	↑	↑	↑	↑
Will be filled in by study team!											
Sum / Day											

BOWL MOVEMENT ASSESSMENT – DAY 3

Today no stool ↑

	Stool consistency				Stool colour					Odour	
No.	watery	soft	modelled	hard	yellow	green	brown	grey	olive-black	normal (sourish)	smelly
1	↑	↑	↑	↑	↑	↑	↑	↑	↑	↑	↑
2	↑	↑	↑	↑	↑	↑	↑	↑	↑	↑	↑
3	↑	↑	↑	↑	↑	↑	↑	↑	↑	↑	↑
4	↑	↑	↑	↑	↑	↑	↑	↑	↑	↑	↑
5	↑	↑	↑	↑	↑	↑	↑	↑	↑	↑	↑
6	↑	↑	↑	↑	↑	↑	↑	↑	↑	↑	↑
7	↑	↑	↑	↑	↑	↑	↑	↑	↑	↑	↑
8	↑	↑	↑	↑	↑	↑	↑	↑	↑	↑	↑
9	↑	↑	↑	↑	↑	↑	↑	↑	↑	↑	↑
10	↑	↑	↑	↑	↑	↑	↑	↑	↑	↑	↑
Will be filled in by study team!											
Sum / Day											

3-DAY DIARY FOR BREASTFEEDING MOTHERS

3-day Diary for Breastfeeding Mothers	*"Suitability of an infant formula with L-5-Methyltetra-hydrofolate for the particular nutritional use in infants"*	2014-05-06-MTHF
Period before: __ (e.g. V2) *(visit every 4 weeks)*		Subject ID: S _ _ _ _

Additionally some questions about possibly occurring events such as "reflux symptoms". If these events do not emerge, just make a tick in the NO column!

REFLUX SYMPTOMS – DAY 1			DIGESTION - DAY 1		
	NO	How often today?		NO	YES
**Belching (moderately)**	↑	___ ___	**Bloating (colic)**	↑	↑
**Posseting (easy, effortless)**	↑	___ ___			
**Vomiting (stronger, heftier)** choking	↑	___ ___			

CRYING - DAY 1			
	Ordinary	Conspicuous	Is conspicuous and related to the formula / milk intake
**The crying behaviour is…**	↑	↑	↑
**SLEEPING TIME - DAY 1**			
______________hours today			

REFLUX SYMPTOMS – DAY 2			DIGESTION - DAY 2		
	NO	How often today?		NO	YES
**Belching (moderately)**	↑	___ ___	**Bloating (colic)**	↑	↑
**Posseting (easy, effortless)**	↑	___ ___			
**Vomiting (stronger, heftier)** choking	↑	___ ___			

3-DAY DIARY FOR BREASTFEEDING MOTHERS

CRYING - DAY 2			
	Ordinary	Conspicuous	Is conspicuous and related to the formula / milk intake
**The crying behaviour is…**	↑	↑	↑
**SLEEPING TIME - DAY 2**			
_______________hours today			

REFLUX SYMPTOMS – DAY 3			DIGESTION - DAY 3		
	NO	How often today?		NO	YES
**Belching (moderately)**	↑	___ ___	**Bloating (colic)**	↑	↑
**Posseting (easy, effortless)**	↑	___ ___			
**Vomiting (stronger, heftier)** choking	↑	___ ___			

CRYING - DAY 3			
	Ordinary	Conspicuous	Is conspicuous and related to the formula / milk intake
**The crying behaviour is…**	↑	↑	↑
**SLEEPING TIME - DAY 3**			
_______________hours today			

3-DAY INFANT FORMULA INTAKE DIARY

**3-day Infant Formula Intake Diary**	*"Suitability of an infant formula with L-5-Methyltetra-hydrofolate for the particular nutritional use in infants"*	**2014-05-06-MTHF**
Period before: __ (e.g. V2)<br>*(visit every 4 weeks)*		**Random-No.: R _ _ _**<br>**Subject ID: S _ _ _ _**

Contact Details:

Enrollment and clinical examinations:<br>**Hipp** GmbH und Co. Vertrieb KG	Recruitment:<br>**KBC "Dr Dragiša Mišović-Dedinje"**	CRO:<br>**Sermon**
**Clinical Study Center**, Belgrade<br>Kneza Milosa 51, Belgrade, Serbia<br><br>+381 (0) 11 3612 945<br>+381 (0) 60 51 50 530<br>hipp@technicom.net	Neonatology<br>Heroja Milana Tepića 1,<br>11000 Belgrade,<br>Serbia<br><br>+381 (0) 63 312 171<br>milica.vusurovic@gmail.com	Donje Svetice 46 C/V,<br>10000 Zagreb,<br>Croatia<br><br>+385 1 550 92 47<br>+385 1 23 34 616<br>ana.gasparovic@sermoncro.com

Dear Parents / Caregiver,

You have received this infant formula (powder, HiPP Fol-SN) from us in connection with your infant's participation in the study, which we would ask you, to give to your baby in a standard infant feeding bottle within the next 16 weeks, as your child desires it (ad libitum). Please prepare the formula according to the instructions on the paper boxes.

Please take notes **three days before the visit** how many bottles and how much of the study infant formula your baby is consuming and what else your infant is drinking/eating (allowed are maximal 50 ml water or tea per day). Please also record when you are applying the formula and other drinks.

The study infant formula should be stored closed in a dry place at room temperature (max 25°C).

Enclosed you will find an Infant Formula **Intake Diary**, in which you should note when, how often and how much of the study formula you have given to your infant. In addition we ask you to record the palatability and toleration ("reflux and digestion symptoms" and fecal assessment) of the formula.

3-DAY INFANT FORMULA INTAKE DIARY

**3-day Infant Formula Intake Diary**	*"Suitability of an infant formula with L-5-Methyltetra-hydrofolate for the particular nutritional use in infants"*	**2014-05-06-MTHF**
Period before: __ (e.g. V2)<br>*(visit every 4 weeks)*		**Random-No.: R _ _ _**<br>**Subject ID: S _ _ _ _**

For the study conduct, it is important that you keep this Infant Formula **Intake Diary** and specify exactly when you have fed your baby and if there were left overs in the bottle. **Please also write down if you gave him/her any medication in the remarks / comments column.** You will get a supply of the study formula for next 4 weeks and also an ***3-day*** Infant Formula **Intake Diary**. Please return the diary at your next appointment at our study site.

We kindly ask you not to dispose the empty 500g-inner-bags of the study formula but to keep it until your next appointment. You have also received from us a bag in which to collect the empty inner-bags of the study formula. We ask you to bring the empty inner bags to your next appointment with us at:

Site: Clinical Study Center, Kneza Milosa 51, Belgrade

On:

______ ________ ____________
Study Day Date Time

Please bring all empty inner-bags, and this 3-day Intake Diary with you to your next appointment. On this date, you will receive the next 4 weeks supply and a new 3-day Intake Diary.

For inquiries to our team, please contact us by phone (+381 (0) 63 312 171) or by e-mail (milica.vusurovic@gmail.com).

Thank you for your cooperation!

Your study team

3-DAY INFANT FORMULA INTAKE DIARY

3-day Infant Formula Intake Diary	*"Suitability of an infant formula with L-5-Methyltetra-hydrofolate for the particular nutritional use in infants"*	2014-05-06-MTHF
Period before: __ (e.g. V2) *(visit every 4 weeks)*		Random-No.: R _ _ _<br>Subject ID: S _ _ _ _

Infant-Formula 3-day Intake Diary Example

Day of Life	*Date*	*Time*	*Bottle No.*	*Water (ml)*	*Spoons of formula*	*Formula left (ml)*	*Other drinks / Remarks*
e.g.51	*DD.MM.YYYY*	*6:00*	*1*	*200ml*	*6*	***20***	
	(DAY 1)	*10:00*	*2*	*150ml*	*4*	-	Fever (paracetamol suppository)
		14:00	*3*	*200ml*	*6*	-	
		18:00	*4*	*100ml*	*3*	-	
		22:00	*5*	*200ml*	*6*	***40***	***30 ml tea***
		...	...	...	...	...	
e.g.52	*DD.MM.YYYY*	*6:00*	*1*	*200ml*	*6*	-	
	(DAY 2)	*10:00*	*2*	*100ml*	*3*	-	
		14:00	*3*	*200ml*	*6*	***20***	
		18:00	*4*	*200ml*	*6*	-	
		22:00	*5*	*150ml*	*4*	-	
		02:00	*6*	*200ml*	*6*	***60***	
		...	...	...	...	...	
e.g.53	*DD.MM.YYYY*	*6:00*	*1*	*200ml*	*6*	-	
	(DAY 3)	*10:00*	*2*	*100ml*	*3*	-	
		14:00	*3*	*200ml*	*6*	***20***	
		18:00	*4*	*200ml*	*6*	-	
		22:00	*5*	*150ml*	*4*	-	
		02:00	*6*	*200ml*	*6*	***60***	
		...	...	...	...	...	

Will be filled in by study team!

	Sum Volume / Total No of Bottles	Formula Volume / Day	Total other drinks volume	Other drinks volume / Day
/				

3-DAY INFANT FORMULA INTAKE DIARY

**3-day Infant Formula Intake Diary**	*"Suitability of an infant formula with L-5-Methyltetra-hydrofolate for the particular nutritional use in infants"*	**2014-05-06-MTHF**
Period before: __ (e.g. V2)<br>*(visit every 4 weeks)*		**Random-No.: R _ _ _**<br>**Subject ID: S _ _ _ _**

Infant-Formula 3-day Intake Diary

Day of Life	*Date*	*Time*	*Bottle No.*	*Water (ml)*	*Spoons of formula*	*Formula left (ml)*	*Other drinks / Remarks*
	(DAY 1)						
	(DAY 2)						
	(DAY 3)						

Will be filled in by study team!

	Sum Volume / Total No of Bottles	Formula Volume / Day	Total other drinks volume	Other drinks volume / Day
/				

3-DAY INFANT FORMULA INTAKE DIARY

**3-day Infant Formula Intake Diary**	*"Suitability of an infant formula with L-5-Methyltetra-hydrofolate for the particular nutritional use in infants"*	**2014-05-06-MTHF**
Period before: __ (e.g. V2)<br>*(visit every 4 weeks)*		**Random-No.: R _ _ _**<br>**Subject ID: S _ _ _ _**

Palatability & Tolerability

of HiPP **Instant Study Formula**

Please describe the consistency, colour and odour of each bowl movement your infant has during the 3-days intake recording phase! The calculations will be conducted by the study team.

**BOWL MOVEMENT ASSESSMENT – DAY 1**											
**Today no stool** ↑											
	**Stool consistency**				**Stool colour**					**Odour**	
No.	watery	soft	modelled	hard	yellow	green	brown	grey	olive-black	normal (sourish)	smelly
**1**	↑	↑	↑	↑	↑	↑	↑	↑	↑	↑	↑
**2**	↑	↑	↑	↑	↑	↑	↑	↑	↑	↑	↑
**3**	↑	↑	↑	↑	↑	↑	↑	↑	↑	↑	↑
**4**	↑	↑	↑	↑	↑	↑	↑	↑	↑	↑	↑
**5**	↑	↑	↑	↑	↑	↑	↑	↑	↑	↑	↑
**6**	↑	↑	↑	↑	↑	↑	↑	↑	↑	↑	↑
**7**	↑	↑	↑	↑	↑	↑	↑	↑	↑	↑	↑
**8**	↑	↑	↑	↑	↑	↑	↑	↑	↑	↑	↑
**9**	↑	↑	↑	↑	↑	↑	↑	↑	↑	↑	↑
**10**	↑	↑	↑	↑	↑	↑	↑	↑	↑	↑	↑
Will be filled in by study team!											
Sum / Day											

3-DAY INFANT FORMULA INTAKE DIARY

BOWL MOVEMENT ASSESSMENT – DAY 2

Today no stool ↑

	Stool consistency				Stool colour					Odour	
No.	watery	soft	modelled	hard	yellow	green	brown	grey	olive-black	normal (sourish)	smelly
**1**	↑	↑	↑	↑	↑	↑	↑	↑	↑	↑	↑
**2**	↑	↑	↑	↑	↑	↑	↑	↑	↑	↑	↑
**3**	↑	↑	↑	↑	↑	↑	↑	↑	↑	↑	↑
**4**	↑	↑	↑	↑	↑	↑	↑	↑	↑	↑	↑
**5**	↑	↑	↑	↑	↑	↑	↑	↑	↑	↑	↑
**6**	↑	↑	↑	↑	↑	↑	↑	↑	↑	↑	↑
**7**	↑	↑	↑	↑	↑	↑	↑	↑	↑	↑	↑
**8**	↑	↑	↑	↑	↑	↑	↑	↑	↑	↑	↑
**9**	↑	↑	↑	↑	↑	↑	↑	↑	↑	↑	↑
**10**	↑	↑	↑	↑	↑	↑	↑	↑	↑	↑	↑
Will be filled in by study team!											
Sum / Day											

BOWL MOVEMENT ASSESSMENT – DAY 3

Today no stool ↑

	Stool consistency				Stool colour					Odour	
No.	watery	soft	modelled	hard	yellow	green	brown	grey	olive-black	normal (sourish)	smelly
**1**	↑	↑	↑	↑	↑	↑	↑	↑	↑	↑	↑
**2**	↑	↑	↑	↑	↑	↑	↑	↑	↑	↑	↑
**3**	↑	↑	↑	↑	↑	↑	↑	↑	↑	↑	↑
**4**	↑	↑	↑	↑	↑	↑	↑	↑	↑	↑	↑
**5**	↑	↑	↑	↑	↑	↑	↑	↑	↑	↑	↑
**6**	↑	↑	↑	↑	↑	↑	↑	↑	↑	↑	↑
**7**	↑	↑	↑	↑	↑	↑	↑	↑	↑	↑	↑
**8**	↑	↑	↑	↑	↑	↑	↑	↑	↑	↑	↑
**9**	↑	↑	↑	↑	↑	↑	↑	↑	↑	↑	↑
**10**	↑	↑	↑	↑	↑	↑	↑	↑	↑	↑	↑
Will be filled in by study team!											
Sum / Day											

3-DAY INFANT FORMULA INTAKE DIARY

**3-day Infant Formula Intake Diary**	*"Suitability of an infant formula with L-5-Methyltetra-hydrofolate for the particular nutritional use in infants"*	**2014-05-06-MTHF**
Period before: __ (e.g. V2)<br>*(visit every 4 weeks)*		**Random-No.: R _ _ _**<br>**Subject ID: S _ _ _ _**

Additionally some questions about possibly occurring events such as "reflux symptoms". If these events do not emerge, just make a tick in the NO column!

**REFLUX SYMPTOMS – DAY 1**			**DIGESTION - DAY 1**		
	NO	How often today?		NO	YES
**Belching (moderately)**	↑	___ ___	**Bloating (colic)**	↑	↑
**Posseting (easy, effortless)**	↑	___ ___			
**Vomiting (stronger, heftier)** choking	↑	___ ___			

**CRYING - DAY 1**			
	Ordinary	Conspicuous	Is conspicuous and related to the formula / milk intake
**The crying behaviour is…**	↑	↑	↑

**SLEEPING TIME - DAY 1**
______________hours today

**REFLUX SYMPTOMS – DAY 2**			**DIGESTION - DAY 2**		
	NO	How often today?		NO	YES
**Belching (moderately)**	↑	___ ___	**Bloating (colic)**	↑	↑
**Posseting (easy, effortless)**	↑	___ ___			
**Vomiting (stronger, heftier)** choking	↑	___ ___			

CRYING - DAY 2			
	Ordinary	Conspicuous	Is conspicuous and related to the formula / milk intake
**The crying behaviour is…**	↑	↑	↑
**SLEEPING TIME - DAY 2**			
_______________hours today			

REFLUX SYMPTOMS – DAY 3		
	NO	How often today?
**Belching (moderately)**	↑	___ ___
**Posseting (easy, effortless)**	↑	___ ___
**Vomiting (stronger, heftier)** choking	↑	___ ___

DIGESTION - DAY 3		
	NO	YES
**Bloating (colic)**	↑	↑

CRYING - DAY 3			
	Ordinary	Conspicuous	Is conspicuous and related to the formula / milk intake
**The crying behaviour is…**	↑	↑	↑
**SLEEPING TIME - DAY 3**			
_______________hours today			

Questions about the study formula:

Please answer the following questions if your infant consumes the study formula!
Use the following ranking: 1 = very much / 5 = not at all.

	**1**	**2**	**3**	**4**	**5**
Does your child like to drink the study formula?					
Is the study formula easy to handle?					

MEFOLIN study: blood collection, sample handling, storage and shipment procedures for baseline visit and visit 4 in infants

Procedure agreed by: Bevital, Konzilijum, DSM ARC, HIPP study center

Blood is collected from study infants for several analyses, which are done in different laboratories. In the infants sampling should be performed at least three hours after the last breast feeding or formula feeding.

	laboratory	parameters	sample	vol (µl)	tube
1	Konzilijum	full blood count	full blood	200 µl	2 ml BD Vacu-tainer
2	Bevital	red cell folate	full blood	50	
3	Bevital	red cell folate	derived from Nr 2.		
4	Bevital	plasma 5-MTHF/oxidized folic acid	plasma	200	
5	Bevital	plasma 5-MTHF/oxidized folic acid	plasma	200	
6	LMU	back up 1	plasma	200	
7	LMU	back up 2	plasma	200	
8	DSM ARC	buffy coat	cell sediment		
9	Konzilijum	ALAT, creatinine	native blood	BD Vacutainer with separation gel in the amount of 600 µl (yellow cork)	

<u>Ad 1, 2, 3, 4, 5, 6, 7, 8:</u>

In the infants according to the routine in the study center a plain needle is used for blood sampling and the blood is allowed to drop into a K_3-EDTA containing collection tube (BD Vacutainer)

After sampling the closed tubes should be turned up-side down 10 times (not vigorously).

<u>Ad 1:</u> Transfer 200 µl of the full EDTA blood into a suitable tube for hematological analysis, label according to the lab requirements and forward to the hematology lab for analysis

Immediately after sample extraction of the blood for hematology put the BD Vacutainer on crushed ice (+4°C for 10 to 15 minutes to achieve fast cooling of the

sample) and protected from light. The tube is transferred on crushed ice to a refrigerator (temperature between +2 and +4°C), where it can be stored for up to 4 hours until further processing of the tube. All further transfers shall be performed at +4°C and with protection from light.

It is advisable to visually examine the aliquot for hematology prior to the start of the testing.

REMARK: Sample must not contain coagulum or have visible threads

No additional refinement of the sample is needed for hematology. Blood count is performed at the day of blood sampling.

Sample stability: 48hrs on room temperature;

Transport: on room temperature during the first 24hrs. Never freeze the sample

Ad 2, 3:

a. Pipette 450 µl of the ascorbic acid solution (detailed preparation instructions below*) into cryotube 1 (Thermo Nunc Cryo Tubes, Cat. No. 377224, 1ml).

b. Add 50 µl of the EDTA blood to this cryotube and mix with the vortex to obtain a homogenous solution (at least 30s of mixing).

c. Pipette 250 µl of the blood and ascorbic acid solution from cryotube 1 into cryotube 2 (Thermo Nunc Cryo Tubes, Cat. No. 377224) to obtain two equal aliquots of the sample.

d. Close the cryotubes and keep them at room temperature for 30 minutes under light protection.

e. At the end of the 30 minutes transfer the cryotubes immediately to the -80ºC freezer.

*ascorbic acid solution: The ascorbic acid solution must be freshly prepared on each day of sample collection. Add 0.25 g of ascorbic acid (PANREAC: 131013, Serbian distributor: PROANALYTICA d.o.o., Beograd) to a 25 ml volumetric flask and fill with doubly distilled water to the 25ml line (the bottom of the meniscus should be on the line). Close the flask and mix by inversion until completely dissolved. Cover with foil to protect from the light.
required equipment: Precision weighing balance, spatula, 25 ml volumetric flask

Ad 4, 5, 6. 7: After withdrawal of blood for samples 1 and 3, respectively, plasma and cells remaining in the collection tube are separated by refrigerated centrifugation at 1500g for 10 min. Immediately after centrifugation transfer the aliquots of the supernatant (plasma) each into the corresponding storage tubes 4 to 7 (Thermo Nunc Cryo Tubes, Cat. No. 377224, 1 ml). Label and freeze the plasma samples at -80°C.

Make sure that there is a little volume (2-3 mm, see pictures below) of plasma left on the cells (volume for tube 7 is flexible)

Ad 8: The buffy coat with the cells builds a thin whitish layer on top of the red blood cells. It will not be removed from the EDTA tube used for blood sampling. After

labeling freeze the EDTA-tube containing the buffy coat and the red blood cells at -80ºC, while standing vertically.

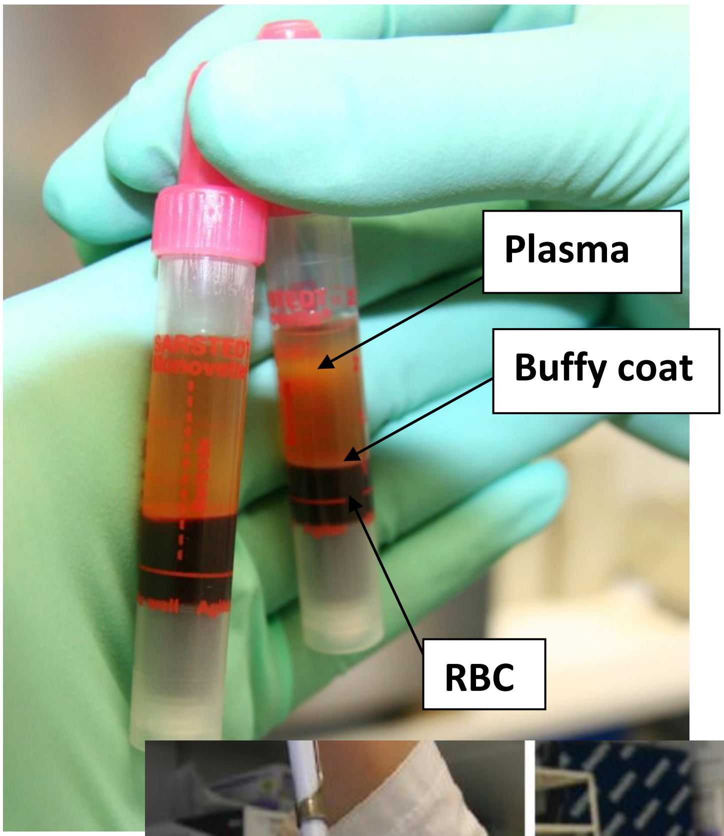

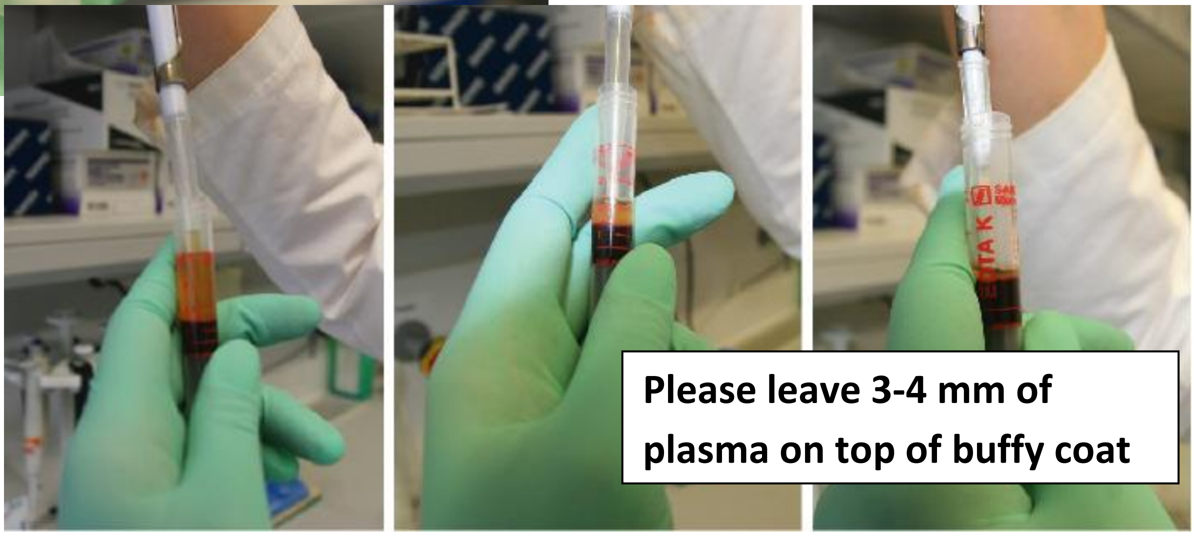

<u>Ad 9:</u>

In the infants after filling the EDTA tube a further volume of 600 µl is dropped into a collection tube without anticoagulant (BD vacutainer)

After allowing 30 – 60 minutes for coagulation at room temperature serum and cells are separated by refrigerated centrifugation at 1500g for 10 min. Immediately after centrifugation the supernatant is transferred into a vial compatible with the measurement procedures. Samples are kept at 4 – 8° until analysis. Analyses are performed at the day of blood sampling.

Lab tracking and documentation

The blood sampling and the further processing of the samples is documented in the MEFOLIN database and in the Biological Sampling Log. Sample lists are sent together with the samples, which the receiving lab can use for checking completeness of the shipment and analyses, respectively. After receipt of samples the receiving lab documents this via email to the project manager (Dr. Regina Goralczyk, DSM Nutritional Products Ltd., regina.goralczyk@dsm.com).

For each blood sampling it has to be documented in the study database:

- Time of blood sample taking
- Time of sample reception in the laboratory for preparation of aliquots 2-7
- Time of completion of blood - ascorbic acid solution reaction
- Time of placing aliquots 2 and 3, as well as 4 to 7 in the -80°C freezer
- Time since last breast/formula feeding
- Volume of last formula feeding (infants in formula groups only)
- A comment on sample quality, in case of deviations from the norm (e.g. haemolysis, lipaemia)

Labeling and transport instructions

- for each subject printed labels (including barcodes) with the corresponding material/analyte identifications (1-9) are provided by LMU München.
- the labels will show MEFOLIN, Subject code, Inf for infant and Mo for mother, Visit number, Aliquot number
- labels will show this information printed and as barcode
- a field will be provided to insert by hand writing the date of sampling
- the aliquots 2 to 7 will be stored at -80°C and shipped after completion of sample collection to the corresponding labs on dry ice (arrange transfer with WORLD Courier).
- aliquot 8 will be stored at -80°C and shipped to the corresponding lab on dry ice in two batches (arrange transfer with WORLD Courier).
- samples will be shipped in 100 sample boxes sorted according to aliquot number
- shipment of samples should preferably be done on Mondays and must be proceeded by an e-mail to the receiving laboratory with information on the date of shipment and the tracking number of the parcel. After arrival receipt is confirmed via email by the laboratory.

Shipment addresses and contact Email:

Aliquots 2, 3, 4, 5:
Bevital AS
Jonas Lies vei 87, 5021 Bergen, Norway
attn: Gry Kvalheim
+47 55974694, +47 90874191
gry@bevital.no

Aliquots 6, 7:
Stoffwechsellabor, Dr von Haunersches Kinderspital
Raum D1.02
Lindwurmstrasse 4, D-80337 München, Germany
attn: Hans Demmelmair
+49 89 4400 53692
Hans.demmelmair@med.uni-muenchen.de

Aliquot 8
DSM Nutritional Products
R&D, Analytical Research Centre (ARC)
Sample Registration Office,
c/o Warenannahme, Bldg. 214
Für: Frau FUCHS Pascale 205/219
Wurmisweg 576
CH-4303 Kaiseraugst / Switzerland
Phone: + 41 (0) 61 815 8629
Email: sample-reg-arc.kaiseraugst@dsm.com

MEFOLIN study: blood and milk collection, sample handling, storage and shipment procedures for visit 2 in breast feeding mothers

Procedure agreed by: Bevital, Konzilijum, HIPP study center

Blood

Blood is collected from breast feeding mothers for analyzing folate related parameters and a blood count, which are done in different laboratories. In the mothers fasted samples in the morning should be obtained.

<table>
<tr><th></th><th>laboratory</th><th>parameters</th><th>sample</th><th>vol (µl)</th><th>tube</th></tr>
<tr><td>1</td><td>Konzilijum</td><td>full blood count</td><td>full blood</td><td>200 µl</td><td rowspan="7">2 ml BD Vacu-tainer</td></tr>
<tr><td>2</td><td>Bevital</td><td>red cell folate</td><td>full blood</td><td>50</td></tr>
<tr><td>3</td><td>Bevital</td><td>red cell folate</td><td colspan="2">derived from Nr 2.</td></tr>
<tr><td>4</td><td>Bevital</td><td>plasma 5-MTHF/oxidized folic acid</td><td>plasma</td><td>200</td></tr>
<tr><td>5</td><td>Bevital</td><td>plasma 5-MTHF/oxidized folic acid</td><td>plasma</td><td>200</td></tr>
<tr><td>6</td><td>LMU</td><td>back up 1</td><td>plasma</td><td>200</td></tr>
<tr><td>7</td><td>LMU</td><td>back up 2</td><td>plasma</td><td>200</td></tr>
</table>

Ad 1, 2, 3, 4, 5, 6, 7:

In the mothers the blood sampling is done according to the clinical routine using one or more BD Vacutainer for collection of 2-3 ml whole blood on EDTA.

After sampling the closed tubes should be turned up-side down 10 times (not vigorously).

Ad 1: Transfer 200 µl of the full EDTA blood into a suitable tube for hematological analysis, label according to the lab requirements and forward to the hematology lab for analysis (unless a separate EDTA tube has been filled for blood count)

Immediately after sample extraction of the blood for hematology put the BD Vacutainer on crushed ice (+4°C for 10 to 15 minutes to achieve fast cooling of the sample) and protected from light. The tube is transferred on crushed ice to a refrigerator (temperature between +2 and +4°C), where it can be stored for up to 4 hours until further processing of the tube. All further transfers shall be performed at +4°C and with protection from light.

It is advisable to visually examine the aliquot for hematology prior to the start of the testing.

REMARK: Sample must not contain coagulum or have visible threads

No additional refinement of the sample is needed for hematology. Blood count is performed at the day of blood sampling.

Sample stability: 48hrs on room temperature;

Transport: on room temperature during the first 24hrs. Never freeze the sample

Ad 2, 3:

a. Pipette 450 µl of the ascorbic acid solution (detailed preparation instructions below*) into cryotube 1 (Thermo Nunc Cryo Tubes, Cat. No. 377224, 1ml).

b. Add 50 µl of the EDTA blood to this cryotube and mix with the vortex to obtain a homogenous solution (at least 30s of mixing).

c. Pipette 250 µl of the blood and ascorbic acid solution from cryotube 1 into cryotube 2 (Thermo Nunc Cryo Tubes, Cat. No. 377224) to obtain two equal aliquots of the sample.

d. Close the cryotubes and keep them at room temperature for 30 minutes under light protection.

e. At the end of the 30 minutes transfer the cryotubes immediately to the -80ºC freezer.

*ascorbic acid solution: The ascorbic acid solution must be freshly prepared on each day of sample collection. Add 0.25 g of ascorbic acid (PANREAC: 131013, Serbian distributor: PROANALYTICA d.o.o., Beograd) to a 25 ml volumetric flask and fill with doubly distilled water to the 25ml line (the bottom of the meniscus should be on the line). Close the flask and mix by inversion until completely dissolved. Cover with foil to protect from the light.
required equipment: Precision weighing balance, spatula, 25 ml volumetric flask

Ad 4, 5, 6. 7: After withdrawal of blood for samples 1 to 3, respectively, plasma and cells remaining in the collection tube are separated by refrigerated centrifugation at 1500g for 10 min. Immediately after centrifugation transfer the aliquots of the supernatant (plasma) each into the corresponding storage tubes 4 to 7 (Thermo Nunc Cryo Tubes, Cat. No. 377224, 1 ml). Label and freeze the plasma samples immediately at -80°C.

Lab tracking and documentation

The blood sampling and the further processing of the samples is documented in the MEFOLIN database and in the Biological Sampling Log. Sample lists are sent together with the samples, which the receiving lab can use for checking completeness of the shipment and analyses, respectively. After receipt of samples the receiving lab documents this via email to the project manager (Dr. Regina Goralczyk, DSM Nutritional Products Ltd., regina.goralczyk@dsm.com).

For each blood sampling it has to be documented in the study database:

- Time of blood sample taking
- Time of sample reception in the laboratory for preparation of aliquots 2-7
- Time of completion of blood - ascorbic acid solution reaction
- Time of placing aliquots 2 and 3, as well as 4 to 7 in the -80°C freezer
- Date and time of last meal
- A comment on sample quality, in case of deviations from the norm (e.g. haemolysis, lipaemia)

Labeling and transport instructions

- for each subject printed labels (including barcodes) with the corresponding material/analyte identifications (1-7) are provided by LMU München.
- the labels will show MEFOLIN, Subject code, Inf for infant and Mo for mother, Visit number, Aliquot number
- labels will show this information printed and as barcode
- a field will be provided to insert by hand writing the date of sampling
- the aliquots 2 to 7 will be stored at -80°C and shipped after completion of sample collection to the corresponding labs on dry ice (arrange transfer with WORLD Courier).
- samples will be shipped in 100 sample boxes sorted according to aliquot number
- shipment of samples should preferably be done on Mondays and must be proceeded by an e-mail to the receiving laboratory with information on the date of shipment and the tracking number of the parcel. After arrival receipt is confirmed via email by the laboratory.

Milk

Breast milk in the MEFOLIN study is collected for the determination of the concentrations of 5-MTHF and oxidized folic acid at visit 2 from breast feeding mothers (reference group).

	laboratory	parameters	sample	vol (ml)
10	DNP R&D Analytics	5-MTHF/oxidized folic acid	milk	1.5
11	DNP R&D Analytics	5-MTHF/oxidized folic acid	milk	1.5
12	LMU	back up	milk	1.5

Sampling of the breast milk is performed at the study center during the visit. The mothers express at least 5 ml of foremilk into a beaker of adequate size. Immediately after sampling study personal transfers 3 portions of 1.5 ml each into 3 Thermo Nunc Cryo Tubes, Cat. No. 368632, 1.8 ml. The tubes are closed, labeled, transferred refrigerated to Konsilium Lab and frozen at -80°C. If immediate freezing of the aliquots at -80°C is not possible, samples can be kept at +4° or lower temperatures for up to 4 hours. All further transfers shall be performed at +4°C or lower temperatures and with protection from light. Samples are stored at -80°C until shipment to the analysing laboratories.

Lab tracking and documentation

The milk sampling and the further processing of the samples is documented in the MEFOLIN database and in the Biological Sampling Log. Sample lists are sent together with the samples, which the receiving lab can use for checking completeness of the shipment and analyses, respectively. After receipt of samples the receiving lab documents this via email to the project manager (Dr. Regina Goralczyk, DSM Nutritional Products Ltd., regina.goralczyk@dsm.com).

For each milk sampling it has to be documented in the study database:

- Time of maternal milk sample collection
- Time of storage at -80°
- Time point of last meal of the mother
- A comment on sample quality, in case of deviations from the norm (e.g. not foremilk, intermittent thawing)

Labeling and transport instructions

- for each subject printed labels (including barcodes) with the corresponding material/analyte identifications (10, 11, 12) are provided by LMU München.
- the labels will show MEFOLIN, Subject code, Milk, Visit number, Aliquot number
- labels will show this information printed and as barcode
- a field will be provided to insert by hand writing the date of sampling

- the aliquots 10 to 12 will be stored at -80°C and shipped after completion of sample collection to the corresponding labs on dry ice (arrange transfer with WORLD Courier).
- samples will be shipped in 100 sample boxes sorted according to aliquot number
- shipment of samples should preferably be done on Mondays and must be proceeded by an e-mail to the receiving laboratory with information on the date of shipment and the tracking number of the parcel. After arrival receipt is confirmed via email by the laboratory.

- **Shipment addresses and contact Email:**

- Aliquots 2, 3, 4, 5:
Bevital AS
Jonas Lies vei 87, 5021 Bergen, Norway
attn: Gry Kvalheim
+47 55974694, +47 90874191
gry@bevital.no

- Aliquots 10, 11:
DSM Nutritional Products
DNP R&D Analytics, NIC-RD/A
Sample Registration Office,
c/o Warenannahme, Bldg. 214
attn: Stephane Etheve
Wurmisweg 576
CH-4303 Kaiseraugst / Switzerland
Phone: + 41 (0) 61 815 8629
Fax: + 41 (0) 61 815 7441
Email: sample-reg-arc.kaiseraugst@dsm.com

- Aliquots 6, 7, 12:
Stoffwechsellabor, Dr von Haunersches Kinderspital
Raum D1.02
Lindwurmstrasse 4, D-80337 München, Germany
attn: Hans Demmelmair
+49 89 4400 53692
Hans.demmelmair@med.uni-muenchen.de

MEFOLIN study: blood collection, sample handling, storage and shipment procedures for visit 5 in infants

Procedure agreed by: Konzilijum, HIPP study center

Blood is collected from study infants for a full blood count at Konzilijum

	laboratory	parameters	sample	vol (µl)	tube
1	Konzilijum	full blood count	full blood	200 µl	Microvette 200

Ad 1:

In the infants according to the routine in the study center a Sarstedt safety lancet (85.1017) is used to penetrate the skin and blood is transferred via the capillary (provided with the Microvette) into a potassium EDTA containing Sarstedt Microvette 200 (20.1288). The Microvette is closed and the sample mixed by inverting.

Ad 1: Transfer the full EDTA blood to the hematology lab for analysis

It is advisable to visually examine the aliquot for hematology prior to the start of the testing.

REMARK: Sample must not contain coagulum or have visible threads

No additional refinement of the sample is needed for hematology. Blood count is performed at the day of blood sampling.

Transport: on room temperature during the first 24hrs. Never freeze the sample

Lab tracking and documentation

The blood sampling and the further processing of the samples is documented in the MEFOLIN database. Immediately after the visit, the obtained sample is ticked in the database.

For each blood sampling it has to be documented in the study database:

- Time of blood sample taking
- A comment on sample quality, in case of deviations from the norm (e.g. haemolysis, lipaemia)

Labeling and transport instructions

- for each subject printed labels (including barcodes) with the corresponding material/analyte identifications are provided by LMU München

- the labels will show MEFOLIN, Subject code, Inf for infant and Mo for mother, Visit number, Aliquot number
- labels will show this information printed and as barcode
- a field will be provided to insert by hand writing the date of sampling

The blood count is performed from fresh material by Konzilijum, thus no sample storage or shipment will be performed.
