# Supplementary material for: Suitability and safety of L-5-methyltetrahydrofolate as a folate source in infant formula: A randomized-controlled trial
Source: PLoS One. 2019 Aug 19;14(8):e0216790. doi: 10.1371/journal.pone.0216790 (PMC6699731; doi:10.1371/journal.pone.0216790)
Supplement: S1 Table — (PDF) [file pone.0216790.s003.pdf]

S1 Table: Formulation of infant formula powder for intervention and control group

	Unit	Per 100 g	Per 100 kcal[a]	Per 100 ml
Energy	kJ	2134		277
	Kcal	510		66
Protein	g	9.6	1.9	1.25
Carbohydrates, of which	g	56.1	11.1	7.3
Lactose	g	54.6	10.8	7.1
Fat, of which	g	27.0	5.3	3.5
Saturated fatty acids	g	8.9	1.8	1.2
Monosaturated fatty acids	g	12.3	2.4	1.6
Polyunsaturated fatty acids, of which	g	5.8	1.1	0.7
Linoleic acid (n-6)	g	5.0	0.9	0.6
Linolenic acid (n-3)	g	0.55	0.1	0.07
Arachidonic acid	mg	95	18.2	12
Docosahexaenoic acid	mg	54	10.6	7.0
Dietary fiber (Galactooligosaccharide)	g	2.3	0.5	0.3
Sodium	g	0.15	0.03	0.02
Potassium	mg	535	106.1	70
Calcium	mg	385	75.8	50
Phosphorus	mg	210	40.9	27
Chloride	mg	345	68.2	45

Magnesium	mg	38	7.6	5.0
Iron	mg	4.0	0.8	0.5
Zinc	mg	4.0	0.8	0.5
Copper	μg	346	68.2	45
Iodine	μg	115	22.7	15
Manganese	μg	58	11.4	7.5
Selenium	μg	10	2.0	1.3
Fluoride	μg	<40	<7.6	<5
Vitamin C	mg	78	15.2	10
Vitamin A	μg	540	106.1	70
Vitamin D	μg	9.0	1.8	1.2
Vitamin E	mg	6.6	1.4	0.90
Vitamin B1	mg	0.45	0.1	0.06
Vitamin B2	mg	0.80	0.2	0.10
Vitamin B6	mg	0.30	0.1	0.04
Vitamin B12	μg	1.15	0.2	0.15
Folic acid[b]	μg	78	15.2	10
Pantothenic acid	mg	3.80	0.8	0.50
Vitamin K	μg	38	7.6	5.0
Biotin	μg	12	2.3	1.5
Niacin	mg	3.1	0.6	0.40

Choline	mg	85	16.7	11
Inositol	mg	30	5.9	3.9

[a] The nutrient contents per 100 kcal as listed above were calculated on basis of the nutrient contents as well as the energy content per 100ml ready-made formula; [b]or equimolar amounts of MTHF, respectively; MTHF: L-5-methyltetrahydrofolate
