# Supplementary material for: Suitability and safety of L-5-methyltetrahydrofolate as a folate source in infant formula: A randomized-controlled trial
Source: PLoS One. 2019 Aug 19;14(8):e0216790. doi: 10.1371/journal.pone.0216790 (PMC6699731; doi:10.1371/journal.pone.0216790)
Supplement: S2 Table — (PDF) [file pone.0216790.s004.pdf]

S2 Table: Baseline characteristics in the modified intention-to-treat population: age, sex and MTHFR polymorphisms C677T (rs1801133) and A1289C (rs1801131)

Parameter	n	Intervention group	Control group	*p* [1]	Reference group
Age [d]	315	21.0 ±3.7	19.7 ±3.6	0.0173	19.6 ±2.6
Sex [% females]	315	43.4%	46.5%	0.6595	52.2%
C677T	292				
CC		33.7%	32.3%		36.4%
CT		53.9%	59.1%		43.6%
TT		12.4%	8.6%	0.6499	20.0%
A1289C	238				
AA		50.6%	44.1%		63.6%
AC		30.3%	50.5%		27.3%
CC		19.1%	5.4%	0.0024	9.1%

Data presented as mean ± SD or percentage; MTHFR: methyltetrahydrofolate reductase n: number of subjects

[1] Intervention and control group was compared, $p < 0.05$ was considered significant, t-test for unequal variance used for age, Chi square test used for sex and genotype
