# Supplementary material for: Suitability and safety of L-5-methyltetrahydrofolate as a folate source in infant formula: A randomized-controlled trial
Source: PLoS One. 2019 Aug 19;14(8):e0216790. doi: 10.1371/journal.pone.0216790 (PMC6699731; doi:10.1371/journal.pone.0216790)
Supplement: S3 Table — (PDF) [file pone.0216790.s005.pdf]

S3 Table: Comparison of parameters of acceptability and tolerance of the intervention formula in the per-protocol population

**Parameter**	**Statistic**	**DF**	***p***
Crying V1 - Control vs. Intervention	0.7674	1	0.3810
Crying V1 - Control vs. Reference	0.0146	1	0.9039
Crying V1 - Intervention vs. Reference	1.0066	1	0.3157
Crying V2 - Control vs. Intervention	2.0226	1	0.1550
Crying V2 - Control vs. Reference	0.0410	1	0.8395
Crying V2 - Intervention vs. Reference	1.6794	1	0.1950
Crying V3 - Control vs. Intervention	0.0379	1	0.8457
Crying V3 - Control vs. Reference	0.3021	1	0.5825
Crying V3 - Intervention vs. Reference	0.5341	1	0.4649
Crying V4 - Control vs. Intervention	2.3534	1	0.1250
Crying V4 - Control vs. Reference	0.9222	1	0.3369
Crying V4 - Intervention vs. Reference	0.6276	1	0.4282
Sleeping hours V1 - Control vs. Intervention	0.6269	1	0.4285
Sleeping hours V1 - Control vs. Reference	0.2571	1	0.6122
Sleeping hours V1 - Intervention vs. Reference	0.0663	1	0.7967
Sleeping hours V2 - Control vs. Intervention	0.0524	1	0.8189

Sleeping hours V2 - Control vs. Reference	0.2479	1	0.6186
Sleeping hours V2 - Intervention vs. Reference	0.3566	1	0.5504
Sleeping hours V3 - Control vs. Intervention	0.0013	1	0.9710
Sleeping hours V3 - Control vs. Reference	1.2635	1	0.2610
Sleeping hours V3 - Intervention vs. Reference	1.0164	1	0.3134
Sleeping hours V4 - Control vs. Intervention	0.010	1	0.9204
Sleeping hours V4 - Control vs. Reference	1.2865	1	0.2567
Sleeping hours V4 - Intervention vs. Reference	1.6424	1	0.2000
Formula Acceptance V1 - Control vs. Intervention	3.9023	1	0.0482
Formula Acceptance V2 - Control vs. Intervention	0.5892	1	0.4427
Formula Acceptance V3 - Control vs. Intervention	5.7817	1	0.0162
Formula Acceptance V4 - Control vs. Intervention	0.3841	1	0.5354
Belching V1 - Control vs. Intervention	1.0278	1	0.3107
Belching V1 - Control vs. Reference	4.8142	1	0.0282
Belching V1 - Intervention vs. Reference	0.6545	1	0.4185
Belching V2 - Control vs. Intervention	4.0248	1	0.0448
Belching V2 - Control vs. Reference	7.1298	1	0.0076
Belching V2 - Intervention vs. Reference	0.2458	1	0.6200

Belching V3 - Control vs. Intervention	0.0482	1	0.8263
Belching V3 - Control vs. Reference	2.2853	1	0.1306
Belching V3 - Intervention vs. Reference	2.5332	1	0.1115
Belching V4 - Control vs. Intervention	1.0030	1	0.3166
Belching V4 - Control vs. Reference	0.6205	1	0.4309
Belching V4 - Intervention vs. Reference	3.5709	1	0.0588
Posseting V1 - Control vs. Intervention	0.1552	1	0.6936
Posseting V1 - Control vs. Reference	1.4193	1	0.2335
Posseting V1 - Intervention vs. Reference	2.5729	1	0.1087
Posseting V2 - Control vs. Intervention	0.2916	1	0.5892
Posseting V2 - Control vs. Reference	0.1003	1	0.7514
Posseting V2 - Intervention vs. Reference	0.0419	1	0.8378
Posseting V3 - Control vs. Intervention	2.1393	1	0.1436
Posseting V3 - Control vs. Reference	0.2372	1	0.6263
Posseting V3 - Intervention vs. Reference	3.8285	1	0.0504
Posseting V4 - Control vs. Intervention	0.0495	1	0.8239
Posseting V4 - Control vs. Reference	1.1148	1	0.2910
Posseting V4 - Intervention vs. Reference	1.3522	1	0.2449

Vomiting V1 - Control vs. Intervention	0.5007	1	0.4792
Vomiting V1 - Control vs. Reference	0.0088	1	0.9253
Vomiting V1 - Intervention vs. Reference	0.3962	1	0.5291
Vomiting V2 - Control vs. Intervention	0.7908	1	0.3738
Vomiting V2 - Control vs. Reference	0.0009	1	0.9759
Vomiting V2 - Intervention vs. Reference	0.6951	1	0.4044
Vomiting V3 - Control vs. Intervention	1.5131	1	0.2187
Vomiting V3 - Control vs. Reference	0.6646	1	0.4150
Vomiting V3 - Intervention vs. Reference	0.2746	1	0.6003
Vomiting V4 - Control vs. Intervention	0.0184	1	0.8921
Vomiting V4 - Control vs. Reference	0.010	1	0.9202
Vomiting V4 - Intervention vs. Reference	0.0019	1	0.9654
Bloating V1 - Control vs. Intervention	0.0399	1	0.8417
Bloating V1 - Control vs. Reference	0.0889	1	0.7656
Bloating V1 - Intervention vs. Reference	0.0122	1	0.9119
Bloating V2 - Control vs. Intervention	0.0459	1	0.8303
Bloating V2 - Control vs. Reference	0.1722	1	0.6781
Bloating V2 - Intervention vs. Reference	0.0342	1	0.8533

Bloating V3 - Control vs. Intervention	3.7096	1	0.0541
Bloating V3 - Control vs. Reference	1.8304	1	0.1761
Bloating V3 - Intervention vs. Reference	0.7144	1	0.3980
Bloating V4 - Control vs. Intervention	0.0931	1	0.7603
Bloating V4 - Control vs. Reference	0.0463	1	0.8296
Bloating V4 - Intervention vs. Reference	0.0218	1	0.8827
Bowel movements V1 - Control vs. Intervention	0.0485	1	0.8258
Bowel movements V1 - Control vs. Reference	14.976	1	0.0001
Bowel movements V1 - Intervention vs. Reference	9.8233	1	0.0017
Bowel movements V2 - Control vs. Intervention	0.5970	1	0.4397
Bowel movements V2 - Control vs. Reference	0.4315	1	0.5112
Bowel movements V2 - Intervention vs. Reference	1.7298	1	0.1884
Bowel movements V3 - Control vs. Intervention	0.2707	1	0.6028
Bowel movements V3 - Control vs. Reference	0.5664	1	0.4517
Bowel movements V3 - Intervention vs. Reference	1.7547	1	0.1853
Bowel movements V4 - Control vs. Intervention	0.9142	1	0.3390
Bowel movements V4 - Control vs. Reference	0.6627	1	0.4156
Bowel movements V4 - Intervention vs. Reference	0.0266	1	0.8704

$p < 0.05$ was considered significant, Mann-Whitney-U was used to compare the groups; DF: degrees of freedom
