# Supplementary material for: Suitability and safety of L-5-methyltetrahydrofolate as a folate source in infant formula: A randomized-controlled trial
Source: PLoS One. 2019 Aug 19;14(8):e0216790. doi: 10.1371/journal.pone.0216790 (PMC6699731; doi:10.1371/journal.pone.0216790)
Supplement: S4 Table — (PDF) [file pone.0216790.s006.pdf]

S5 Table: Summary of adverse events by category and group

Category	Intervention group	Control group	Reference group
Anaemia	1 (33.3%)	1 (33.3%)	1 (33.3%)
Candida infection	1 (33.3%)	0 (0.0%)	2 (66.6%)
Common cold	4 (16.0%)	10 (40.0%)	11 (44.0%)
Ear infection	0 (0.0%)	1 (50.0%)	1 (50.0%)
Fever	0 (0.0%)	0 (0.0%)	1 (100.0%)
Gastrointestinal disorder	1 (33.3%)	1 (33.3%)	1 (33.3%)
Haematoma	1 (100.0%)	0 (0.0%)	0 (0.0%)
Hypertonia	1 (25.0%)	3 (75.0%)	0 (0.0%)
Overweight	1 (33.3%)	1 (33.3%)	1 (33.3%)
Poor weight gain or growth	6 (31.6%)	3 (15.8%)	10 (52.6%)
Prolongued jaundice	1 (33.3%)	0 (0.0%)	2 (66.6%)
Rash, eczma or dry skin	4 (50.0%)	1 (12.5%)	3 (37.5%)
Respiratory tract infection	3 (37.5%)	2 (25.0%)	3 (37.5%)
Torticollis	1 (50.0%)	1 (50.0%)	0 (0.0%)
Umbilical granuloma	0 (0.0%)	0 (0.0%)	1 8100.0%)
Varicella	3 (100.0%)	0 (0.0%)	0 (0.0%)

Note: Denominator is the total number of AE in the respective category; AE: Adverse event
