# Supplementary material for: Suitability and safety of L-5-methyltetrahydrofolate as a folate source in infant formula: A randomized-controlled trial
Source: PLoS One. 2019 Aug 19;14(8):e0216790. doi: 10.1371/journal.pone.0216790 (PMC6699731; doi:10.1371/journal.pone.0216790)
Supplement: S5 Table — (PDF) [file pone.0216790.s007.pdf]

S5 Table: Summary of blood chemistry and hematology parameters

Parameter		Intervention group	Control group	Reference group
**Serum creatinine**				
Baseline	n	114	116	116
	Mean ±SD [μmol/L]	24.7 ±5.4	25.6 ±5.6	27.5 ±6.6
	Normal range[1]	78.9%	84.5%	87.9%
Visit 4	n	89	92	110
	Mean ±SD [μmol/L]	18.9 ±4.3	19.4 ±4.4	18.0 ±4.6
	Normal range[1]	91.0%	91.3%	85.5%
**Alanine aminotransferase**				
Baseline	n	115	116	116
	Mean ±SD [U/L]	26.6 ±11.5	27.6 ±12.1	27.1 ±11.5
	Normal range[2]	90.4%	88.8%	90.5%
Visit 4	n	89	92	110
	Mean ±SD [U/L]	28.7 ±10.6	29.3±8.9	35.4 ±13.6
	Normal range[2]	92.1%	91.3%	70.0%
**Hemoglobin**				
Baseline	n	118	118	117
	Mean ±SD [L/L]	150.9 ±18.3	151.0 ±17.1	150.8 ±17.6
	Normal range[3]	19.5%	17.8%	18.8%
Visit 4	n	90	92	110
	Mean ±SD [g/L]	118.2 ±7.9	119.6 ±7.9	117.1 ±7.7
	Normal range[3]	94.4%	93.5%	94.5%
**Hematocrit**				
Baseline	n	118	118	117

	Mean ±SD [L/L]	0.43 ±0.05	0.34 ±0.05	0.43 ±0.05
	Normal range[4]	53.4%	54.2%	53.0%
Visit 4	n	90	92	110
	Mean ±SD [g/L]	0.34 ±0.02	0.35 ±0.02	0.34 ±0.02
	Normal range[4]	53.3%	63.0%	57.3%
**Mean corpuscular volume**				
Baseline	n	118	118	117
	Mean ±SD [fL]	96.1 ±3.9	96.5 ±4.1	96.0 ±3.9
	Normal range[5]	97.5%	97.5%	98.3%
Visit 4	n	90	92	110
	Mean ±SD [fL]	80.5 ±3.8	81.0 ±3.3	80.3 ±3.6
	Normal range[5]	90.0%	91.3%	95.5%

n: number of subjects; SD: Standard deviation; normal range: [1]serum creatinine: <30 days: 21-75 µmol/L, 30 days to 1 year: 15-37 µmol/L, [2]alanine aminotransferase: ≤1 year: 8-41 U/L; [3]haemoglobin: <30 days: 95-135 g/L, 30 days to 1 year: 105-135 g/L; [4]haematocrit: <30days: 0.32-0.44 L/L, 30 days to 1 year: 0.34-0.44 L/L; [5]mean corpuscular volume: <30 days: 86-104 fL, 30 days to 1 year: 70-86 fL
