# Supplementary material for: Suitability and safety of L-5-methyltetrahydrofolate as a folate source in infant formula: A randomized-controlled trial
Source: PLoS One. 2019 Aug 19;14(8):e0216790. doi: 10.1371/journal.pone.0216790 (PMC6699731; doi:10.1371/journal.pone.0216790)
Supplement: S6 Table — (PDF) [file pone.0216790.s008.pdf]

S6 Table: Gain in body weight, recumbent length and head circumference as well as daily calorie intake per kilo body weight for the modified intention-to-treat and the per-protocol population

Weight gain per day [g/ day]

Modified intention-to-treat population

**Interval**	**Parameter**	**Control group**	**Intervention group**	**Reference group**
Visit 1 to 2	n	96	95	114
	Mean	39.37	37.13	35.36
	SD	9.68	8.94	9.97
	SE	0.99	0.92	0.93
	Median	37.05	38.52	34.81
	P25	33.37	30.49	28.1
	P75	44.73	42.96	40.89
	Min	24.11	8.13	7.41
	Max	83.39	57.14	62.6
Visit 1 to 3	n	95	91	112
	Mean	34.32	33.5	30.8
	SD	7.06	7.15	8.05
	SE	0.72	0.75	0.76
	Median	33.43	33.75	30.24
	P25	28.93	27.86	25.17
	P75	39.39	38.87	35.38
	Min	20.63	17.68	11.61
	Max	55.54	52.77	50.55
Visit 1 to 4	n	95	91	112
	Mean	31.2	30.74	27.68
	SD	5.88	6.07	6.38
	SE	0.6	0.64	0.6
	Median	30.51	30.76	27.63

	P25	26.99	26.31	23.38
	P75	34.35	34.58	31.23
	Min	21.53	15.49	13.96
	Max	51.96	45.99	47.41

Per-protocol population

**Interval**	**Parameter**	**Control group**	**Intervention group**	**Reference group**
Visit 1 to 2	n	83	71	90
	Mean	39.45	36.91	35.44
	SD	9.6	9.46	10.24
	SE	1.05	1.12	1.08
	Median	37.14	37.4	34.64
	P25	33.52	30.77	28.1
	P75	44.29	41.43	40.71
	Min	24.11	8.13	7.41
	Max	83.39	57.14	62.6
Visit 1 to 3	n	82	71	90
	Mean	34.24	33.31	30.81
	SD	6.89	7.28	8.39
	SE	0.76	0.86	0.88
	Median	33.47	33.25	30.31
	P25	29.33	27.86	25.18
	P75	38.75	39.2	35.88
	Min	20.63	17.68	11.61
	Max	55.54	52.77	50.55
Visit 1 to 4	n	83	71	90
	Mean	31.22	30.85	27.64
	SD	5.73	6.14	6.7
	SE	0.63	0.73	0.71

	Median	30.62	30.65	27.63
	P25	27.18	26.31	23.15
	P75	34.05	34.88	31.27
	Min	21.53	19.05	13.96
	Max	51.96	45.99	47.41

Recumbent length gain per day [cm/ day]

Modified intention-to-treat population

Interval	Parameter	Control group	Intervention group	Reference group
Visit 1 to 2	n	96	95	114
	Mean	0.13	0.12	0.12
	SD	0.05	0.04	0.05
	SE	0	0	0
	Median	0.13	0.13	0.12
	P25	0.1	0.1	0.09
	P75	0.16	0.15	0.15
	Min	0.02	0	0
	Max	0.29	0.22	0.24
Visit 1 to 3	n	95	91	112
	Mean	0.12	0.12	0.12
	SD	0.02	0.03	0.03
	SE	0	0	0
	Median	0.12	0.11	0.12
	P25	0.11	0.1	0.1
	P75	0.13	0.13	0.13
	Min	0.06	0.05	0.06
	Max	0.17	0.18	0.25
Visit 1 to 4	n	95	91	112
	Mean	0.11	0.11	0.11

	SD	0.02	0.02	0.02
	SE	0.0	0.0	0.0
	Median	0.11	0.11	0.11
	P25	0.1	0.1	0.09
	P75	0.12	0.12	0.12
	Min	0.07	0.06	0.05
	Max	0.16	0.15	0.16

Per-protocol population

Interval	Parameter	Control group	Intervention group	Reference group
Visit 1 to 2	n	83	71	90
	Mean	0.13	0.12	0.12
	SD	0.05	0.04	0.05
	SE	0.01	0.00	0.01
	Median	0.13	0.13	0.12
	P25	0.11	0.1	0.09
	P75	0.16	0.15	0.15
	Min	0.02	0.00	0.01
	Max	0.29	0.22	0.23
Visit 1 to 3	n	82	71	90
	Mean	0.12	0.11	0.12
	SD	0.02	0.02	0.03
	SE	0.00	0.00	0.00
	Median	0.12	0.11	0.12
	P25	0.11	0.1	0.1
	P75	0.13	0.13	0.13
	Min	0.06	0.05	0.07
	Max	0.17	0.18	0.25
Visit 1 to 4	n	83	71	90

	Mean	0.11	0.11	0.11
	SD	0.02	0.02	0.02
	SE	0.00	0.00	0.00
	Median	0.11	0.11	0.11
	P25	0.1	0.1	0.1
	P75	0.12	0.12	0.12
	Min	0.07	0.06	0.05
	Max	0.16	0.15	0.16

Head circumference gain per day [cm/ day]

Modified intention-to-treat population

Interval	Parameter	Control group	Intervention group	Reference group
Visit 1 to 2	n	96	95	114
	Mean	0.07	0.07	0.07
	SD	0.03	0.03	0.03
	SE	0	0	0
	Median	0.07	0.07	0.07
	P25	0.05	0.05	0.05
	P75	0.09	0.09	0.08
	Min	0	0.02	-0.02
	Max	0.2	0.15	0.14
Visit 1 to 3	n	95	91	112
	Mean	0.06	0.06	0.06
	SD	0.02	0.02	0.01
	SE	0	0	0
	Median	0.06	0.05	0.05
	P25	0.05	0.05	0.05
	P75	0.07	0.07	0.06
	Min	0.01	0.03	0.02

	Max	0.11	0.11	0.09
Visit 1 to 4	n	95	91	112
	Mean	0.05	0.05	0.05
	SD	0.01	0.01	0.01
	SE	0	0	0
	Median	0.05	0.05	0.05
	P25	0.05	0.05	0.05
	P75	0.06	0.06	0.06
	Min	0.02	0.03	0.03
	Max	0.09	0.1	0.08

Per-protocol population

Interval	Parameter	Control group	Intervention group	Reference group
Visit 1 to 2	n	83	71	90
	Mean	0.07	0.07	0.07
	SD	0.03	0.02	0.03
	SE	0	0	0
	Median	0.07	0.07	0.07
	P25	0.05	0.05	0.05
	P75	0.08	0.09	0.08
	Min	0	0.02	0
	Max	0.2	0.15	0.14
Visit 1 to 3	n	82	71	90
	Mean	0.06	0.06	0.06
	SD	0.02	0.02	0.01
	SE	0	0	0
	Median	0.06	0.05	0.05
	P25	0.05	0.05	0.05
	P75	0.07	0.06	0.06

	Min	0.01	0.03	0.02
	Max	0.11	0.11	0.09
Visit 1 to 4	n	83	71	90
	Mean	0.05	0.05	0.05
	SD	0.01	0.01	0.01
	SE	0	0	0
	Median	0.05	0.05	0.05
	P25	0.05	0.05	0.05
	P75	0.06	0.06	0.06
	Min	0.02	0.04	0.03
	Max	0.09	0.1	0.08

Daily calorie intake per kilo body weight [kcal/ day]

Modified intention-to-treat population

**Interval**	**Parameter**	**Control group**	**Intervention group**	**Reference group**
Visit 1	n	99	97	na
	Mean	118.26	116.58	na
	SD	23.21	25.71	na
	SE	2.33	2.61	na
	Median	120.25	112.45	na
	P25	101.35	99.49	na
	P75	132.19	127.01	na
	Min	65.4	56.95	na
	Max	183.13	196.41	na
Visit 2	n	93	91	na
	Mean	111.08	111.62	na
	SD	19.33	20.25	na
	SE	2	2.12	na
	Median	108.29	111.41	na

	P25	97.62	97.33	na
	P75	122.18	124.18	na
	Min	64.67	68.59	na
	Max	181.99	177.73	na
Visit 3	n	93	88	na
	Mean	100	102.97	na
	SD	16.93	19	na
	SE	1.76	2.03	na
	Median	97.78	101.3	na
	P25	89.08	90.03	na
	P75	108.58	110.69	na
	Min	66.93	62.74	na
	Max	167.66	169.45	na
Visit 4	n	94	88	na
	Mean	91.79	96.43	na
	SD	14.4	15.3	na
	SE	1.49	1.63	na
	Median	89.64	94.32	na
	P25	82.13	87.62	na
	P75	98.85	103.73	na
	Min	65.18	56.58	na
	Max	139.7	140.75	na

Per-protocol population

Interval	Parameter	Control group	Intervention group	Reference group
Visit 1	n	81	71	na
	Mean	122.13	117.88	na
	SD	22.2	26.25	na
	SE	2.47	3.12	na

	Median	121.97	112.03	na
	P25	109.29	99.49	na
	P75	133.34	135.83	na
	Min	65.4	56.95	na
	Max	183.13	181.06	na
Visit 2	n	81	69	na
	Mean	112.71	112.67	na
	SD	19.23	18.45	na
	SE	2.14	2.22	na
	Median	113.65	113.2	na
	P25	100.08	100.4	na
	P75	122.59	125.17	na
	Min	64.67	69.02	na
	Max	181.99	156.81	na
Visit 3	n	81	69	na
	Mean	101.5	103.37	na
	SD	16.72	17.43	na
	SE	1.86	2.1	na
	Median	101.14	101.38	na
	P25	92.4	92.32	na
	P75	110.08	111.01	na
	Min	66.93	62.74	na
	Max	167.66	157.6	na
Visit 4	n	82	69	na
	Mean	92.59	97.11	na
	SD	14.81	14.4	na
	SE	1.64	1.73	na
	Median	90.05	94.21	na

	P25	83.48	87.65	na
	P75	98.88	103.74	na
	Min	65.18	59.42	na
	Max	139.7	140.75	na

Max: maximum; Min: minimum; n: number of subjects; P25: 25th percentile; P75: 75th percentile; SD: Standard deviation; SE: Standard error
