# Supplementary material for: Suitability and safety of L-5-methyltetrahydrofolate as a folate source in infant formula: A randomized-controlled trial
Source: PLoS One. 2019 Aug 19;14(8):e0216790. doi: 10.1371/journal.pone.0216790 (PMC6699731; doi:10.1371/journal.pone.0216790)
Supplement: S7 Table — (PDF) [file pone.0216790.s009.pdf]

S7 Table: Fixed effects solution for the model for the gain in weight, recumbent length, head circumference and calorie intake in the modified intention-to-treat and per-protocol population

Weight gain (Primary outcome)

Modified intention-to-treat population

**Parameter**		**Estimate**	**SE**	**DF**	***t***	***p***	**95% CI**	
							**Lower limit**	**Upper limit**
Intercept		-102.02	158.47	196	-0.64	0.5205	-414.53	210.50
Group	Intervention*	-26.0253	41.9917	371	-0.62	0.5358	-108.60	56.5463
Age		47.7945	1.0173	189	46.98	<.0001	45.7877	49.8013
Age x group	Intervention*	-0.3224	0.8701	371	-0.37	0.7112	-2.0333	1.3885
Age x age		-0.1180	0.005748	371	-20.53	<.0001	-0.1293	-0.1067
Gender	Male**	94.9163	35.4450	371	2.68	0.0077	25.2180	164.61
Birth weight		0.8615	0.04439	371	19.41	<.0001	0.7742	0.9488
$\tau^2_{Intercept}$		61574	9360.66		6.58#	<.0001		
τ^2_{Age}		31.7698	3.6998		8.59#	<.0001		

Per-protocol population

Parameter		Estimate	SE	DF	*t*	*p*	95% CI	
							Lower limit	Upper limit
Intercept		-36.6595	176.98	151	-0.21	0.8362	-386.34	313.02
group	Intervention*	-25.8946	45.7137	305	-0.57	0.5715	-115.85	64.0595
age		47.1965	1.1344	152	41.60	<.0001	44.9553	49.4378
age*group	Intervention*	-0.2161	0.9626	305	-0.22	0.8225	-2.1102	1.6780
age*age		-0.1144	0.006555	305	-17.45	<.0001	-0.1273	-0.1015
gender	Male**	50.0266	39.7719	305	1.26	0.2094	-28.2355	128.29
birth weight		0.8515	0.04945	305	17.22	<.0001	0.7542	0.9488
$\tau^2_{Intercept}$		54809	9651.31		5.68#	<.0001		
τ^2_{Age}		31.3540	4.0785		7.69#	<.0001		

Gain in recumbent length

Modified intention-to-treat population

Parameter		Estimate	SE	DF	*t*	*p*	95% CI	
							Lower limit	Upper limit
Intercept		41.5931	0.7799	196	53.33	<.0001	40.0550	43.1312
Ggroup	Intervention*	0.09410	0.2093	372	0.45	0.6532	-0.3174	0.5056
Age		0.1118	0.001738	189	64.29	<.0001	0.1083	0.1152
Age x group	Intervention*	-0.00339	0.002487	372	-1.37	0.1730	-0.00828	0.001495

Age x age		0.7336	0.1763	372	4.16	<.0001	0.3870	1.0803
Gender	Male**	0.002903	0.000221	372	13.15	<.0001	0.002469	0.003337
Birth weight		41.5931	0.7799	196	53.33	<.0001	40.0550	43.1312
**$\tau^2_{Intercept}$**		0.9987	0.2449		4.08#	<.0001		
**τ^2_{Age}**		0.000099	0.000033		2.98#	0.0015		

Per-protocol population

Parameter		Estimate	SE	DF	*t*	*p*	95% CI	
							Lower limit	Upper limit
Intercept		41.2100	0.8905	151	46.28	<.0001	39.4505	42.9695
group	Intervention*	0.3135	0.2351	306	1.33	0.1834	-0.1492	0.7761
age		0.1116	0.001807	152	61.74	<.0001	0.1080	0.1152
age*group	Intervention*	-0.00402	0.002665	306	-1.51	0.1320	-0.00927	0.001219
age*age		0.7438	0.2023	306	3.68	0.0003	0.3457	1.1419
gender	Male**	0.002999	0.000252	306	11.92	<.0001	0.002504	0.003494
birth weight		41.2100	0.8905	151	46.28	<.0001	39.4505	42.9695
**$\tau^2_{Intercept}$**		0.9665	0.2714		3.56#	0.0002		
**τ^2_{Age}**		0.000082	0.000035		2.37#	0.0090		

**Gain in head circumference**								
**Modified intention-to-treat population**								
**Parameter**		**Estimate**	**SE**	**DF**	***t***	***p***	**95% CI**	
							**Lower limit**	**Upper limit**
Intercept		30.9782	0.5265	196	58.83	<.0001	29.9397	32.0166
Ggroup	Intervention*	0.1718	0.1478	372	1.16	0.2458	-0.1188	0.4624
Age		0.05418	0.001159	189	46.73	<.0001	0.05190	0.05647
Age x group	Intervention*	-0.00069	0.001658	372	-0.41	0.6789	-0.00395	0.002574
Age x age		0.5446	0.1188	372	4.58	<.0001	0.3109	0.7782
Gender	Male**	0.001209	0.000149	372	8.13	<.0001	0.000917	0.001502
Birth weight		30.9782	0.5265	196	58.83	<.0001	29.9397	32.0166
$\tau^2_{Intercept}$		0.5773	0.1194		4.84#	<.0001		
τ^2_{Age}		0.000047	0.000015		3.13#	0.0009		
**Per-protocol population**								
**Parameter**		**Estimate**	**SE**	**DF**	***t***	***p***	**95% CI**	
							**Lower limit**	**Upper limit**
Intercept		30.8458	0.5717	151	53.95	<.0001	29.7162	31.9754
group	Intervention*	0.1887	0.1630	306	1.16	0.2479	-0.1321	0.5095
age		0.05333	0.001223	152	43.59	<.0001	0.05091	0.05575
age*group	Intervention*	-0.00023	0.001804	306	-0.13	0.8984	-0.00378	0.003319

age*age		0.5572	0.1295	306	4.30	<.0001	0.3024	0.8120
gender	Male**	0.001246	0.000161	306	7.74	<.0001	0.000929	0.001563
birth weight		30.8458	0.5717	151	53.95	<.0001	29.7162	31.9754
$\tau^2_{Intercept}$		0.5477	0.1247		4.39#	<.0001		
τ^2_{Age}		0.000047	0.000016		3.01#	0.0013		

Increase in calorie intake

Modified intention-to-treat population

Parameter		Estimate	SE	DF	*t*	*p*	95% CI	
							Lower limit	Upper limit
Intercept		479.45	47.5774	194	10.08	<.0001	385.62	573.29
Ggroup	Intervention*	-32.3493	17.4380	358	-1.86	0.0644	-66.6431	1.9445
Age		1.4235	0.1494	185	9.53	<.0001	1.1288	1.7182
Age x group	Intervention*	0.4788	0.2138	358	2.24	0.0257	0.05837	0.8993
Age x age		-48.0932	10.3980	358	-4.63	<.0001	-68.5421	-27.6443
Gender	Male**	0.005214	0.01294	358	0.40	0.6872	-0.02023	0.03066
Birth weight		30.9782	0.5265	196	58.83	<.0001	29.9397	32.0166
$\tau^2_{Intercept}$		7902.68	1609.62		4.91#	<.0001		
τ^2_{Age}		1.0092	0.2423		4.17#	<.0001		

Per-protocol population

Parameter		Estimate	SE	DF	*t*	*p*	95% CI	
							Lower limit	Upper limit
Intercept		478.77	49.1662	149	9.74	<.0001	381.62	575.93
group	Intervention*	-44.4813	19.4842	298	-2.28	0.0231	-82.8253	-6.1372
age		1.3185	0.1618	150	8.15	<.0001	0.9989	1.6382
age*group	Intervention*	0.5745	0.2383	298	2.41	0.0165	0.1055	1.0434
age*age		-44.8800	10.8439	298	-4.14	<.0001	-66.2203	-23.5398
gender	Male**	0.009624	0.01344	298	0.72	0.4744	-0.01682	0.03607
birth weight		478.77	49.1662	149	9.74	<.0001	381.62	575.93
$\tau^2_{Intercept}$		7509.68	1754.22		4.28#	<.0001		
τ^2_{Age}		1.0020	0.2654		3.78#	<.0001		

95% CI: Confidence interval; DF: Degree of freedom; SE: Standard error; * Control is the reference category; ** Female is the reference category; # z value
