# Supplementary material for: Suitability and safety of L-5-methyltetrahydrofolate as a folate source in infant formula: A randomized-controlled trial
Source: PLoS One. 2019 Aug 19;14(8):e0216790. doi: 10.1371/journal.pone.0216790 (PMC6699731; doi:10.1371/journal.pone.0216790)
Supplement: S8 Table — (PDF) [file pone.0216790.s010.pdf]

S8 Table: Least square means for body weight, recumbent length, head circumference and calorie intake at visit 4 for the modified intention-to-treat and the per protocol population

**Body weight**									
**Modified intention-to-treat population**									
Parameter	Age [days]	Birth weight [g]	Estimate	SE	DF	*t*	*p*	95% CI	
								Lower limit	Upper limit
Intervention	112	3448.6	6727.03	58.8608	371	114.29	<.0001	6611.29	6842.77
Control	112	3448.6	6789.16	58.0967	371	116.86	<.0001	6674.92	6903.40
Intervention - Control	112	3448.6	-62.13	82.5163	371	-0.75	0.4520	-224.39	100.13
**Per-protocol population**									
Parameter	Age [days]	Birth weight [g]	Estimate	SE	DF	*t*	*p*	95% CI	
								Lower limit	Upper limit
Intervention	112	3448.6	6717.90	69.2637	305	96.99	<.0001	6581.60	6854.19
Control	112	3448.6	6767.99	64.0847	305	105.61	<.0001	6641.89	6894.10
Intervention - Control	112	3448.6	-50.0934	94.1236	305	-0.53	0.5950	-235.31	135.12
**Recumbent length**									
**Modified intention-to-treat population**									
Parameter	Age [days]	Birth weight [g]	Estimate	SE	DF	*t*	*p*	95% CI	
								Lower limit	Upper limit

Intervention	112	3448.6	64.2020	0.1713	372	374.82	<.0001	63.8652	64.5388
Control	112	3448.6	64.4881	0.1683	372	383.15	<.0001	64.1572	64.8191
Intervention - Control	112	3448.6	-0.2861	0.2401	372	-1.19	0.2341	-0.7582	0.1860
**Per-protocol population**									
Parameter	Age [days]	Birth weight [g]	Estimate	SE	DF	*t*	*p*	95% CI	
								Lower limit	Upper limit
Intervention	112	3439.0	64.2569	0.1983	306	324.02	<.0001	63.8667	64.6471
Control	112	3439.0	64.3942	0.1834	306	351.08	<.0001	64.0333	64.7551
Intervention - Control	112	3439.0	-0.1373	0.2700	306	-0.51	0.6115	-0.1373	0.2700
**Head circumference**									
**Modified intention-to-treat population**									
Parameter	Age [days]	Birth weight [g]	Estimate	SE	DF	*t*	*p*	95% CI	
								Lower limit	Upper limit
Intervention	112	3448.6	41.5843	0.1090	372	381.37	<.0001	41.3699	41.7988
Control	112	3448.6	41.4895	0.1070	372	387.58	<.0001	41.2790	41.7000
Intervention - Control	112	3448.6	0.09484	0.1527	372	0.62	0.5350	-0.2055	0.3952
**Per-protocol population**									
Parameter	Age [days]	Birth weight [g]	Estimate	SE	DF	*t*	*p*	95% CI	
								Lower limit	Upper limit

Intervention	112	3439.0	41.5454	0.1216	306	341.75	<.0001	41.3062	41.7846
Control	112	3439.0	41.3825	0.1124	306	368.06	<.0001	41.1612	41.6037
Intervention - Control	112	3439.0	0.1629	0.1655	306	0.98	0.3258	-0.1628	0.4886

Calorie intake

Modified intention-to-treat population

Parameter	Age [days]	Birth weight [g]	Estimate	SE	DF	*t*	*p*	95% CI	
								Lower limit	Upper limit
Intervention	28	3448.6	494.30	9.3310	358	52.97	<.0001	475.95	512.65
Control	28	3448.6	513.24	9.1923	358	55.83	<.0001	495.17	531.32
Intervention - Control	28	3448.6	-18.9424	13.1052	358	-1.45	0.1492	-44.7153	6.8305
Intervention	56	3448.6	547.57	7.5074	358	72.94	<.0001	532.80	562.33
Control	56	3448.6	553.10	7.3906	358	74.84	<.0001	538.57	567.64
Intervention - Control	56	3448.6	-5.5354	10.5394	358	-0.53	0.5998	-26.2624	15.1915
Intervention	84	3448.6	600.83	7.8957	358	76.10	<.0001	585.30	616.36
Control	84	3448.6	592.96	7.7285	358	76.72	<.0001	577.76	608.16
Intervention - Control	84	3448.6	7.8715	11.0490	358	0.71	0.4767	-13.8576	29.6006
Intervention	112	3448.6	654.09	10.2475	358	63.83	<.0001	633.94	674.25
Control	112	3448.6	632.81	9.9912	358	63.34	<.0001	613.17	652.46
Intervention - Control	112	3448.6	21.2784	14.3091	358	1.49	0.1379	-6.8621	49.4189

Per-protocol population

Parameter	Age [days]	Birth weight [g]	Estimate	SE	DF	*t*	*p*	95% CI	
								Lower limit	Upper limit
Intervention	28	3439.0	497.95	10.5694	298	47.11	<.0001	477.15	518.75
Control	28	3439.0	526.35	9.8257	298	53.57	<.0001	507.01	545.69
Intervention - Control	28	3439.0	-28.3963	14.4333	298	-1.97	0.0501	-56.8005	0.007772
Intervention	56	3439.0	550.96	8.2270	298	66.97	<.0001	534.77	567.15
Control	56	3439.0	563.27	7.6341	298	73.78	<.0001	548.24	578.29
Intervention - Control	56	3439.0	-12.3114	11.2263	298	-1.10	0.2737	-34.4044	9.7816
Intervention	84	3439.0	603.96	8.4640	298	71.36	<.0001	587.30	620.62
Control	84	3439.0	600.19	7.8136	298	76.81	<.0001	584.81	615.56
Intervention - Control	84	3439.0	3.7735	11.5224	298	0.33	0.7435	-18.9020	26.4491
Intervention	112	3439.0	656.96	11.1166	298	59.10	<.0001	635.09	678.84
Control	112	3439.0	637.10	10.2403	298	62.22	<.0001	616.95	657.26
Intervention - Control	112	3439.0	19.8585	15.1170	298	1.31	0.1900	-9.8912	49.6081

95% CI: Confidence interval; DF: Degree of freedom; SE: Standard error
