# Supplementary material for: Suitability and safety of L-5-methyltetrahydrofolate as a folate source in infant formula: A randomized-controlled trial
Source: PLoS One. 2019 Aug 19;14(8):e0216790. doi: 10.1371/journal.pone.0216790 (PMC6699731; doi:10.1371/journal.pone.0216790)
Supplement: S9 Table — (PDF) [file pone.0216790.s011.pdf]

S9 Table: Least square means for body weight at visit 4 according to treatment-gender and -genotype interaction model for the modified intention-to-treat and the per protocol population

**Body weight according to treatment-gender interaction model**									
**Modified intention-to-treat population**									
Parameter	Age [days]	Birth weight [g]	Estimate	SE	DF	*t*	*p*	95% CI	
								Lower limit	Upper limit
Intervention	112	3448.6	6691.96	56.8628	371	117.69	<.0001	6580.15	6803.78
Control	112	3448.6	6776.19	55.5929	371	121.89	<.0001	6666.87	6885.51
Intervention - Control	112	3448.6	-84.2263	79.3589	371	-1.06	0.2892	-240.28	71.8233
**Per-protocol population**									
Parameter	Age [days]	Birth weight [g]	Estimate	SE	DF	*t*	*p*	95% CI	
								Lower limit	Upper limit
Intervention	112	3439.0	6682.33	65.1069	306	102.64	<.0001	6554.22	6810.45
Control	112	3439.0	6752.41	60.1220	306	112.31	<.0001	6634.11	6870.72

Intervention - Control	112	3439.0	-70.0824	88.4235	306	-0.79	0.4286	-244.08	103.91

Body weight according to treatment-genotype (C677T) interaction model

Modified intention-to-treat population

Parameter	Age [days]	Birth weight [g]	Estimate	SE	DF	*t*	*p*	95% CI	
								Lower limit	Upper limit
Intervention	112	3449.3	6698.11	74.8298	360	89.51	<.0001	6550.95	6845.27
Control	112	3449.3	6736.65	82.3551	360	81.80	<.0001	6574.69	6898.61
Intervention - Control	112	3449.3	-38.5358	111.07	360	-0.35	0.7288	-256.96	179.89

Per-protocol population

Parameter	Age [days]	Birth weight [g]	Estimate	SE	DF	*t*	*p*	95% CI	
								Lower limit	Upper limit
Intervention	112	3435.7	6679.94	84.5427	298	79.01	<.0001	6513.56	6846.31
Control	112	3435.7	6726.22	84.9905	298	79.14	<.0001	6558.96	6893.48

Intervention - Control	112	3435.7	-46.2821	119.64	298	-0.39	0.6992	-281.73	189.17

Body weight according to treatment-genotype (A1289C) interaction model

Modified intention-to-treat population

Parameter	Age [days]	Birth weight [g]	Estimate	SE	DF	*t*	*p*	95% CI	
								Lower limit	Upper limit
Intervention	112	3449.3	6742.42	67.4710	360	99.93	<.0001	6609.74	6875.11
Control	112	3449.3	6917.19	96.6451	360	71.57	<.0001	6727.13	7107.25
Intervention - Control	112	3449.3	-174.77	117.64	360	-1.49	0.1383	-406.12	56.5823

Per-protocol population

Parameter	Age [days]	Birth weight [g]	Estimate	SE	DF	*t*	*p*	95% CI	
								Lower limit	Upper limit
Intervention	112	3435.7	6717.13	75.3604	298	89.13	<.0001	6568.82	6865.44
Control	112	3435.7	6899.66	98.9184	298	69.75	<.0001	6704.99	7094.32

Intervention - Control	112	3435.7	-182.53	124.08	298	-1.47	0.1423	-426.71	61.6551

95% CI: Confidence interval; DF: Degree of freedom; SE: Standard error
