# Supplementary figures and images for: Suitability and safety of L-5-methyltetrahydrofolate as a folate source in infant formula: A randomized-controlled trial
Source: PLoS One. 2019 Aug 19;14(8):e0216790. doi: 10.1371/journal.pone.0216790 (PMC6699731; doi:10.1371/journal.pone.0216790)

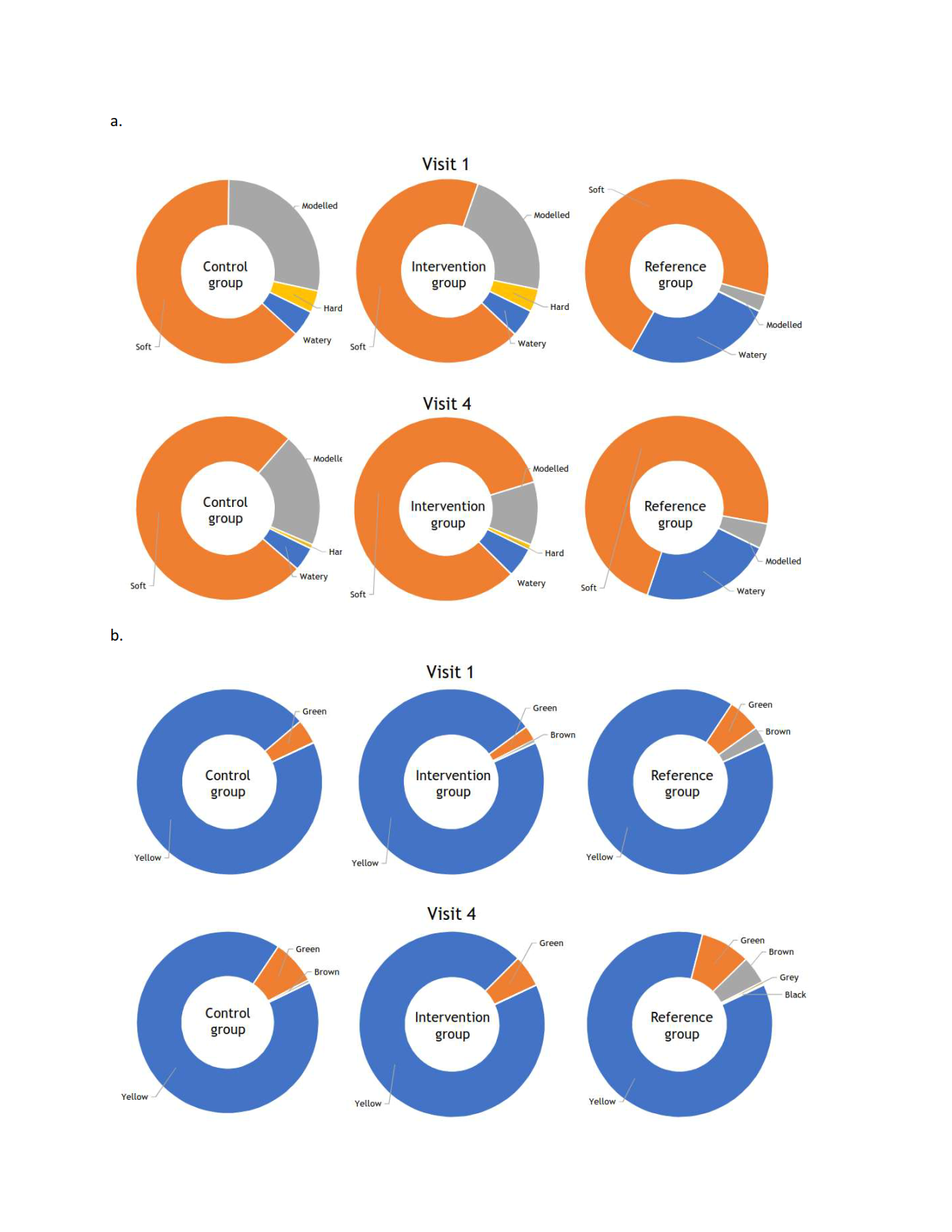

Supplement: S1 Fig — Consistency (a.), color (b.) and smelliness of stool at visits 1 and 4 in the per protocol population. (TIF) [file pone.0216790.s012.tif]
